# The E3 ligase TRIM29 drives renal ischemia-reperfusion injury by targeting DUSP10 for proteasomal degradation

**DOI:** 10.1038/s41419-026-08990-w

**Published:** 2026-06-13

**Authors:** Jinshan Cui, Jinjin Feng, Yunlong Liu, Yafeng Fan, Yonghao Zhan, Jin Tao, Xuepei Zhang, Hao Liu

**Affiliations:** Department of Urology, The First Affiliated Hospital of Zhengzhou University, Zhengzhou, China

**Keywords:** Acute kidney injury, Acute inflammation

## Abstract

Renal ischemia-reperfusion injury (RIRI) represents a leading cause of acute kidney injury (AKI), yet the molecular determinants governing stress-induced signaling remain incompletely understood. Here, we identify TRIM29, an E3 ubiquitin ligase significantly upregulated in human and murine AKI, as a pivotal regulator of ischemic kidney injury. Using integrative transcriptomic and proteomic profiling, we show that TRIM29 directly interacts with and ubiquitinates DUSP10, a MAPK-specific phosphatase that restrains JNK/p38 activity. TRIM29 catalyzes K48-linked ubiquitination of DUSP10 at lysine 231, promoting its proteasomal degradation. The consequent depletion of DUSP10 sustains MAPK activation, which subsequently amplifies NF-κB-mediated inflammation and tubular epithelial cell apoptosis. Conversely, genetic ablation of TRIM29 in mice confers marked protection against renal dysfunction, structural damage, and inflammatory infiltration. Mechanistically, the TRIM29–DUSP10–MAPK axis defines a complete stress-responsive cascade that links ischemic stress to maladaptive tubular injury. Our findings reveal a previously unrecognized ubiquitin-dependent pathway governing post-ischemic renal pathology and identify the TRIM29–DUSP10 interface as a promising therapeutic target for AKI.

**Figure:**
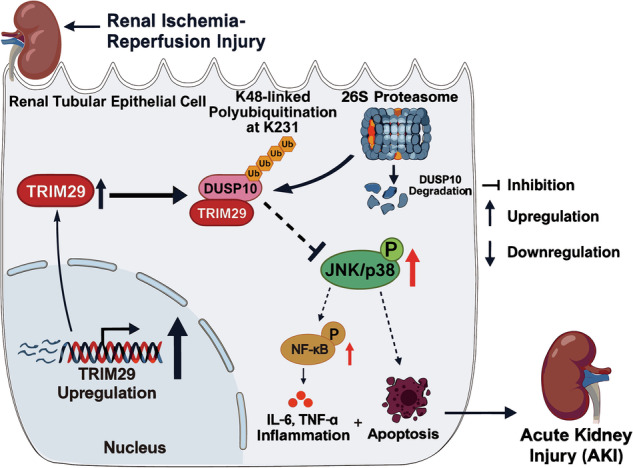
**The TRIM29–DUSP10–MAPK signaling axis in renal ischemia-reperfusion injury**. TRIM29 targets DUSP10 for K48-linked polyubiquitination at lysine 231, promoting its proteasomal degradation and thereby sustaining JNK/p38 MAPK activation to drive maladaptive injury.

**The TRIM29–DUSP10–MAPK signaling axis in renal ischemia-reperfusion injury**. TRIM29 targets DUSP10 for K48-linked polyubiquitination at lysine 231, promoting its proteasomal degradation and thereby sustaining JNK/p38 MAPK activation to drive maladaptive injury.

## Introduction

Acute kidney injury (AKI) remains a major global health burden, characterized by abrupt loss of renal function and high morbidity and mortality rates [1]. Among its diverse etiologies, renal ischemia-reperfusion injury (RIRI) represents the predominant cause, occurring frequently in cardiac surgery, transplantation, and sepsis [2]. Ischemic insult initiates a complex cascade of oxidative stress, inflammation, and apoptosis, leading to tubular epithelial dysfunction and microvascular collapse [3]. Despite decades of investigation, the molecular mechanisms determining whether injured kidneys undergo adaptive recovery or progress to chronic fibrosis remain incompletely understood [4]. Deciphering these mechanisms is essential for developing therapeutic strategies to mitigate post-ischemic renal injury.

The mitogen-activated protein kinase (MAPK) pathways, including JNK and p38, are central mediators of cellular stress, inflammation, and apoptosis, and their activation is a well-established hallmark of ischemia-reperfusion injury across multiple organs [5–7]. In hepatic IRI, JNK activation promotes hepatocyte apoptosis, while in cerebral ischemia, p38 MAPK drives neuronal inflammation and death [8]. Mirroring these observations, sustained activation of the JNK/p38 MAPK pathway is a hallmark of renal IRI that amplifies tubular damage [9]. The duration and magnitude of MAPK activation are tightly modulated by phosphorylation–dephosphorylation dynamics, primarily orchestrated by the dual-specificity phosphatases (DUSPs) [10]. Among them, DUSP10 (MKP5) serves as a key negative regulator of JNK/p38 activity and has been shown to confer protection against inflammatory and ischemic injury in several tissues [8, 11, 12]. Dysregulation of this balance contributes to sustained kinase activation and maladaptive injury responses. However, the upstream molecular mechanisms that control DUSP10 stability during renal ischemia remain largely unknown.

Mounting evidence underscores the critical role of post-translational modifications in regulating cellular stress responses during AKI [13]. Ubiquitination, a key post-translational modification, plays a fundamental role in regulating cellular responses to stress [14]. This process is primarily mediated by E3 ubiquitin ligases, which confer substrate specificity and dictate the outcome of downstream signaling [15]. Among them, members of the tripartite motif (TRIM) family have emerged as critical regulators of protein stability and stress adaptation [16]. Several TRIM members have been implicated in kidney injury: TRIM65 promotes mitochondrial dysfunction through VDAC1 ubiquitination [17], whereas TRIM21 facilitates AKI-to-CKD transition by suppressing p62-mediated autophagy [18]. In contrast, the function of TRIM29—a multifunctional E3 ligase known to orchestrate DNA repair and antiviral responses [19]—has not been explored in the context of renal pathology.

Herein, we identify TRIM29 as a previously unrecognized pathogenic regulator of ischemic AKI. Genetic ablation of TRIM29 confers robust protection against tubular apoptosis, inflammation, and renal dysfunction. Mechanistically, as illustrated in the graphical abstract, integrative transcriptomic, proteomic, and biochemical analyses reveal that TRIM29 targets DUSP10 for K48-linked polyubiquitination at lysine 231, promoting its proteasomal degradation and thereby sustaining JNK/p38 MAPK activation to drive maladaptive injury. These findings establish the TRIM29–DUSP10–MAPK axis as a promising therapeutic target for AKI and related renal disorders.

## Results

### TRIM29 is upregulated in renal tissues and tubular cells under ischemic stress

**Fig. 1:**
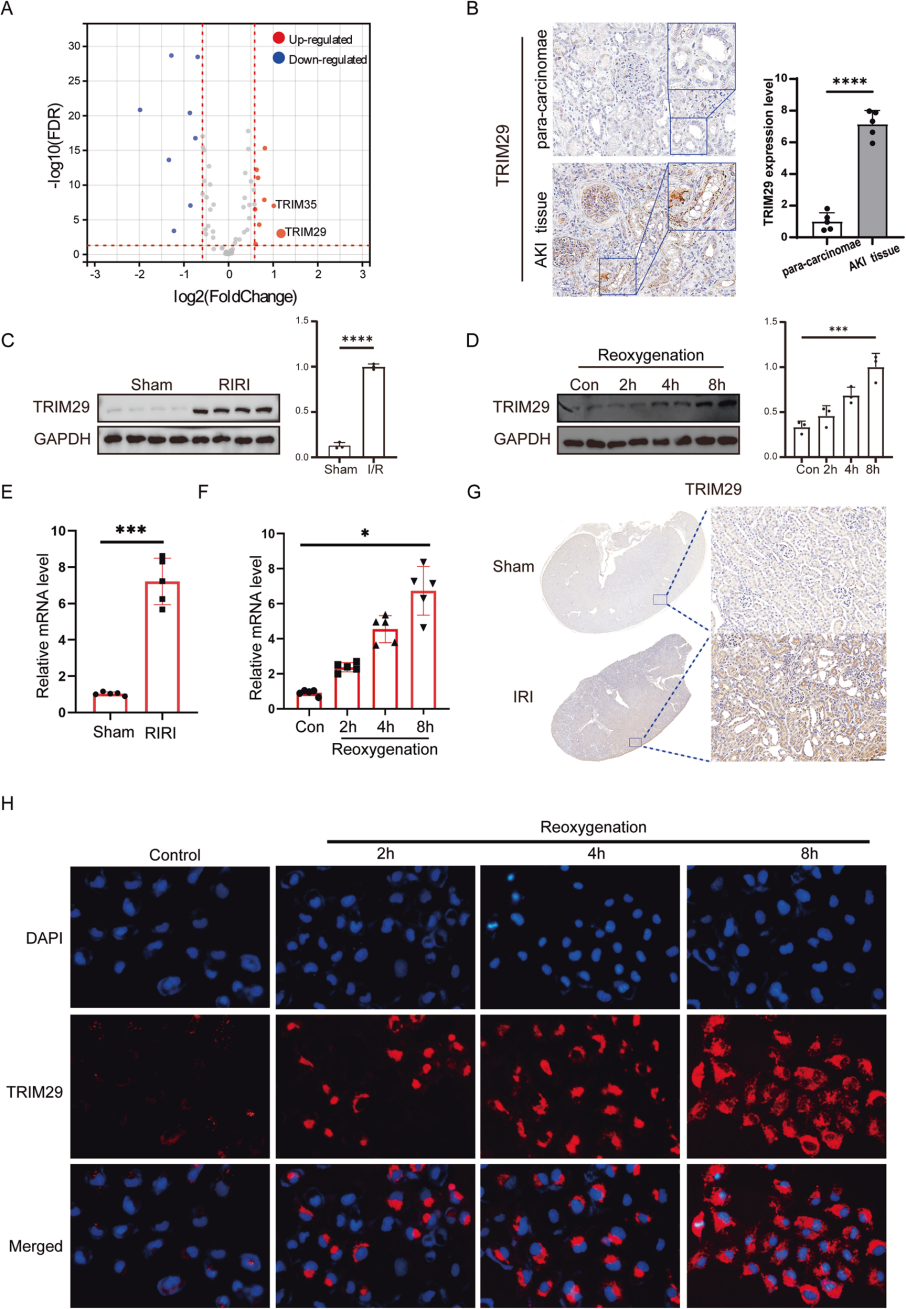
TRIM29 is upregulated in renal tissues and tubular cells under ischemic stress. **A** Differential expression analysis of ubiquitination-related genes in the GSE192883 dataset comparing Sham and RIRI kidney tissues. **B** Representative immunohistochemistry (IHC) staining and quantification confirming significant TRIM29 upregulation in human kidney biopsies from AKI patients compared to normal controls (*n* = 5 per group). **C** Representative western blot analysis and quantification of TRIM29 protein levels in kidney tissues from Sham and RIRI mice (*n* = 3 per group). **D** Western blot analysis and quantification of TRIM29 expression in HK-2 cells subjected to hypoxia/reoxygenation (H/R) for the indicated reperfusion times (*n* = 3 independent experiments). qRT-PCR analysis of TRIM29 mRNA levels in mouse kidney tissues (**E**, *n* = 5 per group) and HK-2 cells (**F**, *n* = 5 independent experiments). **G** Representative immunohistochemistry (IHC) staining of TRIM29 in kidney sections from Sham and RIRI mice. **H** Representative immunofluorescence (IF) images showing TRIM29 intensity in HK-2 cells following H/R treatment. Data are presented as mean ± SEM. Differences between two groups were determined using Student’s t-test. Comparisons among multiple groups were analyzed using one-way ANOVA followed by Tukey’s post-hoc test. *P < 0.05, ***P < 0.001. Each experiment was repeated at least three times independently.

To identify key regulators of RIRI at the transcriptomic level, we first analyzed data from a public database. Using “AKI” or “RIRI-AKI” as keywords, we screened and obtained the GEO dataset GSE192883. Differential expression analysis focusing on ubiquitination-related genes revealed that TRIM29 mRNA was markedly upregulated in RIRI tissues compared to Sham controls (Fig. 1A). Clinical validation via immunohistochemistry (IHC) staining of human kidney biopsies confirmed significant TRIM29 upregulation in AKI patients compared to normal controls (Fig. 1B). We further validated these findings in experimental models, where TRIM29 protein levels were significantly elevated in RIRI mouse kidneys (Fig. 1C) and H/R-treated HK-2 cells in a time-dependent manner (Fig. 1D). qRT-PCR confirmed this transcript-level upregulation (Fig. 1E, F), which is mechanistically driven by the transcription factor KLF10 through its direct binding to and transactivation of the *TRIM29* promoter (Fig. S1). Furthermore, IHC staining of mouse kidney sections revealed that TRIM29 expression was significantly higher in the RIRI group than in the Sham group (Fig. 1G). Correspondingly, cellular immunofluorescence results indicated that the fluorescence intensity of TRIM29 in HK-2 cells also progressively increased with prolonged reoxygenation time after H/R treatment (Fig. 1H).

### TRIM29 deficiency protects against renal RIRI and cellular H/R injury by mitigating inflammation and apoptosis

**Fig. 2:**
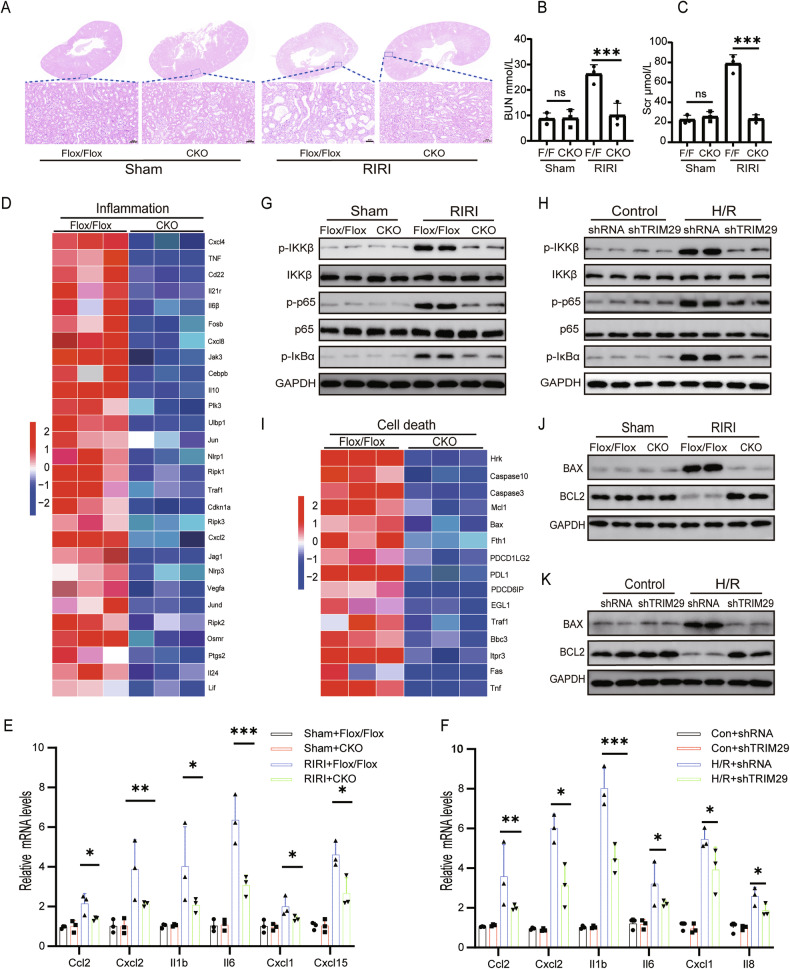
TRIM29 deficiency protects against renal RIRI and cellular H/R injury by mitigating inflammation and apoptosis. **A** Representative H&E staining of kidney tissue sections from TRIM29 ^*Flox/Flox*^ and TRIM29 CKO mice in the Sham group and 24 h post-reperfusion. Assessment of renal function by measuring serum creatinine (SCr) (**B**) and blood urea nitrogen (BUN) (**C**) levels in TRIM29 ^*Flox/Flox*^ and TRIM29 CKO mice in the Sham group and 24 h post-reperfusion (*n* = 3 per group). **D** Heatmap showing differentially expressed inflammatory genes in kidney tissues from TRIM29 ^*Flox/Flox*^ and TRIM29 CKO mice following RIRI treatment. **E** qRT-PCR analysis of mRNA expression of inflammatory cytokines, including *Ccl2*, *Cxcl2*, *Il1b*, *Il6*, and *Cxcl15*, in kidney tissues. **F** qRT-PCR analysis of inflammatory cytokines in HK-2 cells transfected with shNC or shTRIM29 under hypoxia/reoxygenation (H/R) conditions. **G** Western blot analysis of NF-κB pathway activation (p-IKKβ, p-65, and p-IκBα) in kidney tissue lysates. **H** Western blot analysis of NF-κB pathway activation (p-IKKβ, p-65, and p-IκBα) in HK-2 cells with TRIM29 knockdown. **I** Heatmap displaying the transcriptomic profile of apoptosis-related genes in kidney tissues from TRIM29 ^*Flox/Flox*^ and TRIM29 CKO mice following RIRI treatment. **J** Western blot analysis of BAX and BCL-2 protein levels in kidney tissues. **K** Western blot analysis of apoptosis markers in HK-2 cells. Data are presented as mean ± SEM. Two-way ANOVA was applied. *P < 0.05, **P < 0.01, ***P < 0.001. Each experiment was repeated at least three times independently.

To determine the functional role of TRIM29 in the pathogenesis of RIRI, we generated renal tubule-specific TRIM29 knockout (CKO) mice (Fig. S2A–D). Compared with TRIM29 ^*Flox/Flox*^ littermates, genetic ablation of TRIM29 conferred marked nephroprotection against RIRI. Histologically, TRIM29 deficiency significantly ameliorated renal tissue damage, as evidenced by reduced tubular dilation, brush border loss, and cast formation (Fig. 2A), which was confirmed by a significant reduction in the quantified tubular injury score (Fig. S2E). Accompanying these structural improvements, renal functional impairment was attenuated, indicated by the substantial reduction in serum creatinine and BUN levels (Fig. 2B, C).

To explore the underlying molecular mechanisms, we performed transcriptomic profiling on kidney tissues. The analysis unveiled a broad suppression of inflammatory pathways in CKO mice, characterized by the significant downregulation of cytokines such as Cxcl4, Tnf, and Il6 (Fig. 2D). This was corroborated by qPCR, which showed that the RIRI-induced upregulation of Ccl2 and Cxcl2 was reversed in CKO mice (Fig. 2E). Parallelly, in HK-2 cells, shRNA-mediated knockdown of TRIM29 efficiently blunted the H/R-induced inflammatory response at the mRNA level (Fig. 2F).

Substantiating these findings at the protein level, TRIM29 deletion significantly suppressed RIRI-triggered NF-κB activation in vivo, as evidenced by notably decreased phosphorylation of IKKβ, p65, and IκBα (Figs. 2G and S2F). Consistent with the in vivo results, silencing TRIM29 in vitro abrogated the H/R-induced phosphorylation of the NF-κB cascade (Figs. 2H and S2G), confirming TRIM29 as a critical modulator of the inflammatory response.

Additionally, transcriptomic analysis indicated that TRIM29 deletion downregulated a cluster of pro-apoptotic genes, including Hrk and Caspase-3 (Fig. 2I). Immunoblotting further demonstrated that TRIM29 deficiency attenuated the RIRI-induced upregulation of BAX and restored BCL-2 expression (Figs. 2J and S2H). Of note, consistent results were obtained in vitro: TRIM29 knockdown in HK-2 cells reversed the H/R-induced aberrant expression of BAX and BCL-2 (Figs. 2K and S2I). Collectively, these data indicate that TRIM29 deficiency affords robust protection against renal RIRI by suppressing inflammation and apoptosis.

### TRIM29 promotes ischemic renal injury through activation of the JNK/p38 MAPK signaling pathway

**Fig. 3:**
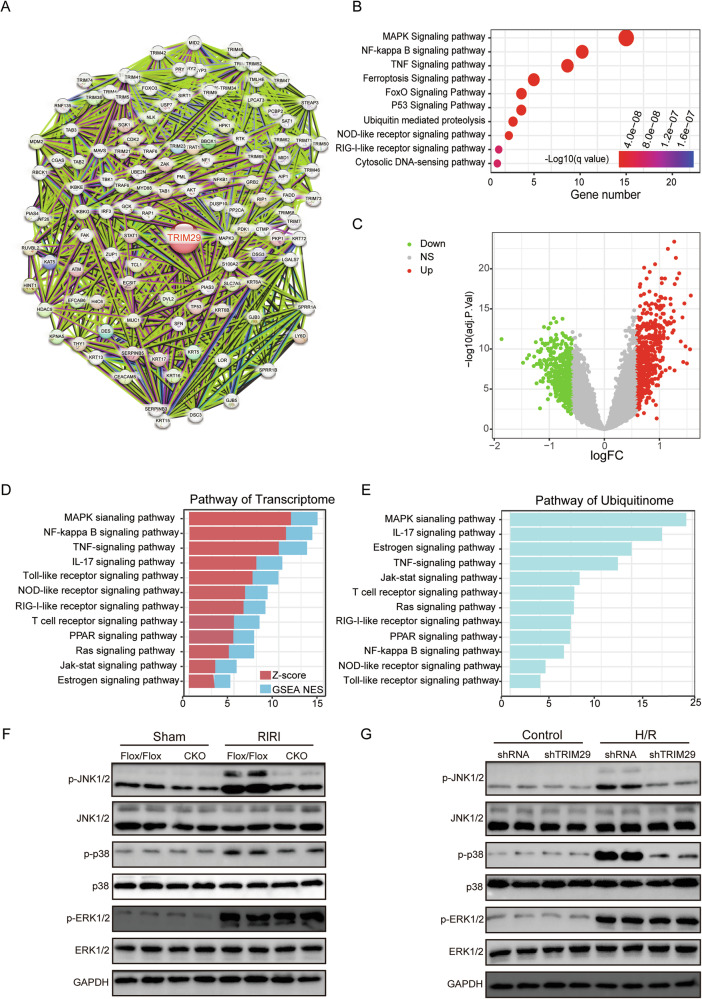
TRIM29 promotes ischemic renal injury through activation of the JNK/p38 MAPK signaling pathway. **A** Protein-protein interaction (PPI) network analysis based on public database (GSE192883) data, identifying TRIM29 as a hub gene among differentially expressed genes. **B** KEGG pathway enrichment analysis of upregulated genes from the public dataset, highlighting the significant enrichment of MAPK and NF-κB signaling pathways. **C** Volcano plot showing differentially expressed genes (DEGs) in kidney tissues between TRIM29 ^*Flox/Flox*^ and TRIM29 CKO mice subjected to RIRI. **D** Gene Set Enrichment Analysis (GSEA) revealing the significantly enriched signaling pathways between TRIM29 ^*Flox/Flox*^ and TRIM29 CKO mice. **E** Ubiquitinomics analysis assessing the 13 pathways presented in (**D**) based on their differential ubiquitination following TRIM29 knockdown in HK-2 cells subjected to H/R. **F** Western blot analysis of p-JNK, JNK, p-p38, p38, p-ERK, and ERK levels in kidney tissues from TRIM29 ^*Flox/Flox*^ and TRIM29 CKO mice subjected to Sham or RIRI operation. **G** Western blot analysis of JNK and p38 phosphorylation in HK-2 cells transfected with shNC or shTRIM29 under Control or hypoxia/reoxygenation (H/R) conditions. Each experiment was repeated at least three times independently.

To dissect the signaling networks regulated by TRIM29, we first performed a bioinformatic analysis of the public GSE192883 dataset. Protein-protein interaction (PPI) network analysis positioned TRIM29 as a central hub gene (Fig. 3A), while KEGG pathway enrichment analysis indicated significant activation of MAPK and NF-κB signaling pathways (Fig. 3B). To substantiate these public findings, we interrogated our internal multi-omics datasets. Transcriptome profiling identified numerous differentially expressed genes (Fig. 3C), with subsequent GSEA highlighting the MAPK pathway as the most significantly enriched signaling cascade (Fig. 3D). Consistently, independent ubiquitinomics analysis confirmed that differentially modified proteins were predominantly mapped to this same MAPK network (Fig. 3E).

Validation at the protein level confirmed these bioinformatic findings. In vivo, RIRI robustly triggered JNK and p38 phosphorylation in TRIM29 ^*Flox/Flox*^ mice; however, this activation was markedly blunted in TRIM29 CKO mice (Figs. 3F and S2J). Notably, ERK phosphorylation remained unaffected, indicating pathway selectivity. Parallelly, in HK-2 cells, H/R stimulation induced JNK/p38 hyperactivation, an effect that was efficiently abrogated by TRIM29 knockdown (Figs. 3G and S2K). Collectively, these data demonstrate that TRIM29 promotes renal injury specifically by amplifying the JNK/p38 MAPK signaling axis.

### TRIM29 directly interacts with and negatively regulates DUSP10 at the post-translational level

**Fig. 4:**
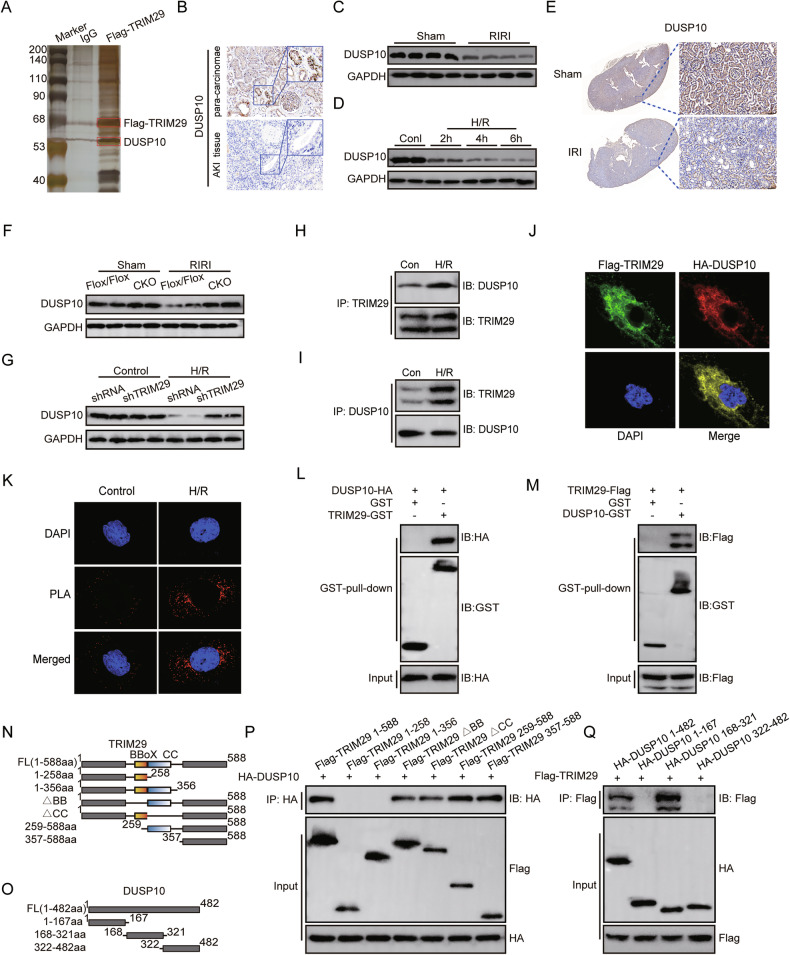
TRIM29 directly interacts with and negatively regulates DUSP10. **A** Silver staining of immunoprecipitates from HK-2 cells transfected with Flag-TRIM29. **B** Representative IHC staining of DUSP10 in human kidney biopsy samples from normal controls and AKI patients (*n* = 5 per group). Western blot analysis of DUSP10 protein levels in kidney tissues from Sham and RIRI mice (**C**) and in HK-2 cells under Control or H/R conditions (**D**). **E** Representative IHC staining of DUSP10 in kidney sections from **mice** in the Sham and RIRI groups. **F** Western blot analysis of DUSP10 protein levels in kidney tissues from *TRIM29 Flox/Flox* and *TRIM29 CKO* mice subjected to Sham or RIRI operation. **G** Western blot analysis of DUSP10 protein levels in HK-2 cells transfected with shNC or shTRIM29 under Control or H/R conditions. **H**, **I** Co-IP assays analyzing the endogenous interaction between TRIM29 and DUSP10 in HK-2 cells under Control and H/R conditions. **J** Representative immunofluorescence (IF) images showing co-localization of TRIM29 (green) and DUSP10 (red) in the cytoplasm of 293T cells. **K** Representative PLA images detecting the endogenous interaction between TRIM29 and DUSP10 in HK-2 cells. Scale bar: 10 μm. **L**, **M** GST pull-down assays using purified proteins to confirm direct interaction. Schematic diagrams of TRIM29 (**N**) and DUSP10 (**O**) full-length and truncation mutants. Co-IP assays in HEK293T cells identifying the binding domains of TRIM29 (**P**) and DUSP10 (**Q**). Each experiment was repeated at least three times independently.

The activation of MAPK signaling pathways is precisely regulated by a dynamic balance between phosphorylation and dephosphorylation. To elucidate the mechanism governing MAPK regulation, we performed immunoprecipitation (IP) followed by silver staining and mass spectrometry (MS) in HK-2 cells, identifying the MAPK phosphatase DUSP10 as a high-confidence TRIM29 interactor (Figs. 4A and S3A). Clinical IHC confirmed significant DUSP10 downregulation in AKI patients (Figs. 4B and S3B). Consistently, DUSP10 protein levels were markedly depleted in RIRI tissues (Figs. 4C and S3C) and H/R-treated cells (Figs. 4D and S3D), exhibiting an inverse expression pattern relative to TRIM29. Correspondingly, IHC staining revealed a marked reduction of DUSP10 expression in mouse kidney sections following RIRI (Fig. 4E). Notably, this injury-induced downregulation was rescued by TRIM29 ablation in mice (Figs. 4F and S3E) and by TRIM29 knockdown in HK-2 cells (Figs. 4G and S3F), suggesting that TRIM29 negatively regulates DUSP10 at the protein level. By contrast, while TRIM29 also interacted with PARP1 (Fig. S3G), PARP1 stability was unaffected by TRIM29 manipulation (Fig. S3H), ruling it out as a degradation target.

To further substantiate the interaction between TRIM29 and DUSP10, we performed endogenous co-immunoprecipitation (Co-IP) assays in HK-2 cells. Immunoprecipitation with an anti-TRIM29 antibody successfully co-precipitated DUSP10 (Fig. 4H), and reciprocally, an anti-DUSP10 antibody pulled down TRIM29 (Fig. 4I). Notably, while a basal interaction was detectable in the control group, the amount of co-precipitated DUSP10 (or TRIM29) was markedly enhanced following H/R treatment, suggesting that cellular stress promotes the formation of this complex. To explore their spatial relationship, we performed immunofluorescence staining, which revealed a distinct co-localization of TRIM29 and DUSP10 within the cytoplasm of 293 T cells (Fig. 4J). To further verify their physical interaction in situ at a higher resolution, we employed Proximity Ligation Assays (PLA). As anticipated, prominent PLA signals (red puncta) indicative of endogenous TRIM29–DUSP10 complexes were observed, and the interaction intensity was significantly enhanced under H/R conditions compared to controls (Fig. 4K). Subsequently, GST pull-down assays using purified GST-TRIM29 with His-DUSP10 (Fig. 4L) and GST-DUSP10 with Flag-TRIM29 (Fig. 4M) confirmed a direct physical interaction.

To identify the binding interface, we generated truncation mutants for TRIM29 and DUSP10 (Fig. 4N, O). Co-IP assays unveiled that the C-terminal region (357-588 aa) of TRIM29 is required for binding to DUSP10 (Fig. 4P), and reciprocally, TRIM29 specifically binds to the 168–321 aa segment of DUSP10 (Fig. 4Q). Importantly, *Dusp10* mRNA levels remained unchanged across groups (Fig. S3I, J), confirming that TRIM29 negatively regulates DUSP10 through post-translational destabilization rather than transcriptional repression.

### DUSP10 overexpression inhibits MAPK/NF-κB activation and apoptosis to protect against renal ischemia-reperfusion injury (RIRI)

**Fig. 5:**
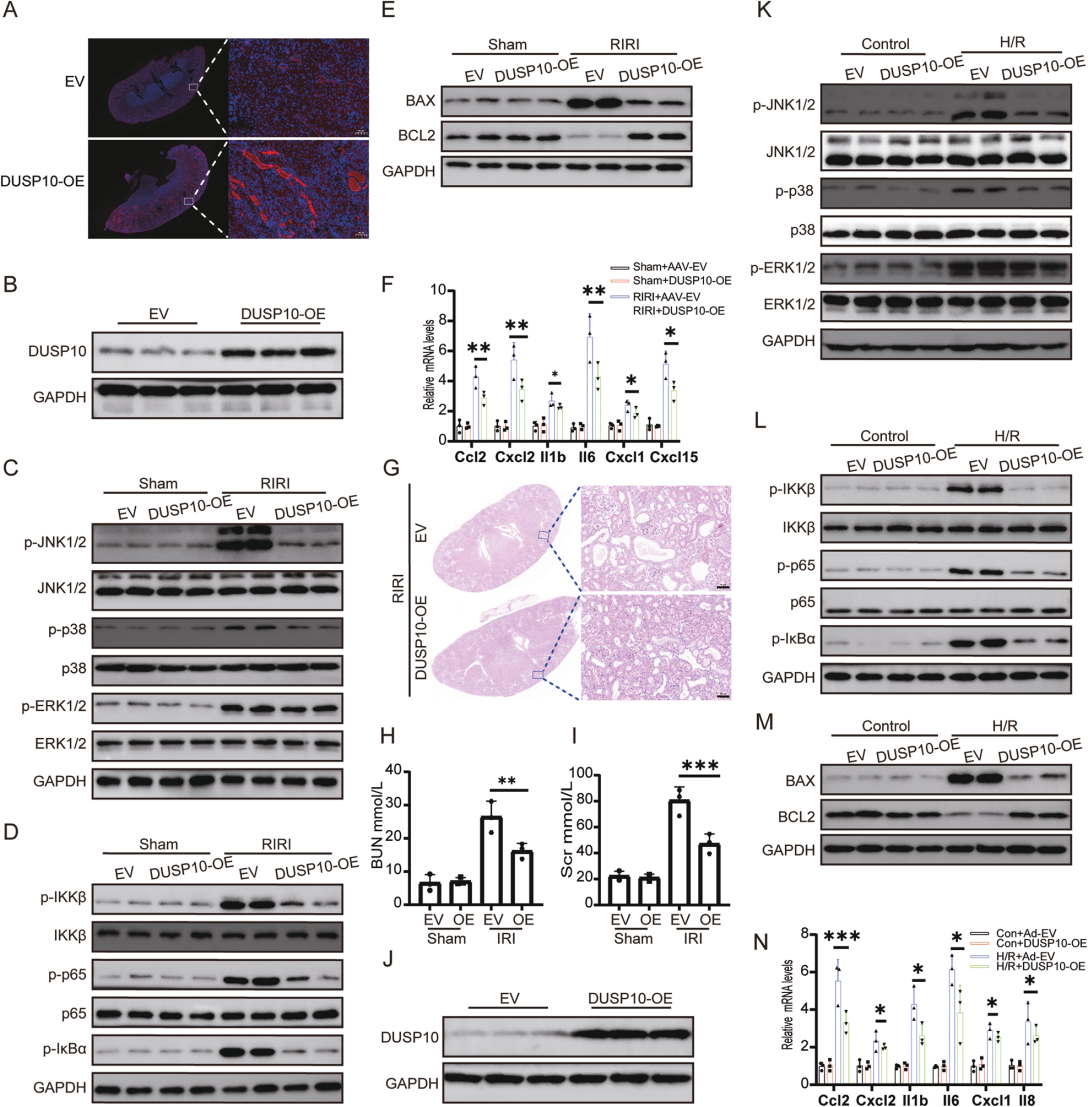
DUSP10 overexpression mitigates renal ischemia-reperfusion injury (RIRI). **A** Representative immunofluorescence (IF) staining validating DUSP10 overexpression in the renal cortex of mice following AAV9-DUSP10 (DUSP10-OE) injection. **B** Western blot analysis confirming the overexpression efficiency of DUSP10 protein in kidney tissues from mice administered AAV9-EV or AAV9-DUSP10. Western blot analysis of MAPK pathway components (**C**, p-JNK, p-p38, and p-ERK), NF-κB pathway components (**D**, p-IKKβ, p-p65, and p-IκBα), and apoptosis markers (**E**, BAX and BCL-2) in kidney tissues. Mice were administered the indicated AAV9 vectors (EV or DUSP10-OE) 2 weeks prior to Sham or RIRI operation. **F** qRT-PCR analysis of inflammatory cytokines (*Ccl2, Cxcl2, Il1b, Il6*, and *Cxcl15*) in kidney tissues from mice pre-treated with AAV9-EV (EV) or AAV9-DUSP10 (DUSP10-OE) followed by RIRI. **G** Representative H&E staining of kidney sections from mice pre-treated with the indicated vectors and subjected to RIRI. Serum creatinine (**H**) and BUN (**I**) levels in the indicated groups. **J** Western blot validation of DUSP10 overexpression in HK-2 cells transduced with Ad-DUSP10 (DUSP10-OE) compared to Ad-EV (EV). Western blot analysis of MAPK pathway components (**K**, p-JNK, p-p38, and p-ERK), NF-κB pathway components (**L**, p-IKKβ, p-p65, and p-IκBα), and apoptosis markers (**M**, BAX and BCL-2) in HK-2 cells. Cells were transduced with Ad-EV (EV) or Ad-DUSP10 (DUSP10-OE) for 48 h prior to Control or H/R stimulation. **N** qRT-PCR analysis of inflammatory cytokines (*Ccl2, Cxcl2, Il1b, Il6*, and *Il8*) in HK-2 cells pre-transduced with the indicated vectors and subsequently exposed to H/R. Data are presented as mean ± SEM. Differences between two groups were determined using Student’s t-test. For comparisons involving two or more independent variables, two-way ANOVA was applied. *P < 0.05, **P < 0.01, ***P < 0.001. EV, empty vector; DUSP10-OE, DUSP10 overexpression. Each experiment was repeated at least three times independently.

To interrogate the therapeutic potential of DUSP10, we performed gain-of-function studies in vivo and in vitro. For the in vivo experiments, mice were administered AAV9-DUSP10 or AAV9-EV via tail vein injection 2 weeks prior to the establishment of RIRI. Robust overexpression of DUSP10 in the renal cortex was confirmed by immunofluorescence and Western blot (Figs. 5A, B and S3K).

Mechanistically, DUSP10 overexpression efficiently dampened the I/R-induced hyperphosphorylation of JNK and p38, while ERK phosphorylation remained largely unaffected, confirming the substrate specificity of DUSP10 (Figs. 5C and S3L). Concurrently, the activation of the NF-κB signaling cascade (p-IKKβ, p-p65, and p-IκBα) was markedly inhibited in kidneys pre-treated with AAV9-DUSP10 compared to controls (Figs. 5D and S3M).

Functionally, prior restoration of DUSP10 levels attenuated apoptotic signaling, as evidenced by reduced BAX and elevated BCL-2 levels (Figs. 5E and S3N). Furthermore, it significantly lowered the mRNA expression of a broad spectrum of inflammatory cytokines, including Ccl2, Cxcl2, Il1b, Il6, and Cxcl15 (Fig. 5F). Accompanying these molecular changes, DUSP10 overexpression markedly ameliorated histological damage (Figs. 5G and S3O) and preserved renal function, indicated by lower SCr and BUN levels (Fig. 5H, I).

Consistent with in vivo results, in vitro experiments were conducted by transducing HK-2 cells with Ad-DUSP10 or Ad-EV for 48 h prior to H/R stimulation. Validation of overexpression was confirmed by immunoblotting (Figs. 5J and S3P). Under H/R conditions, pre-transduction with Ad-DUSP10 significantly suppressed MAPK/NF-κB activation (Figs. 5K, L and S3Q, R), inhibited apoptosis (Figs. 5M and S3S), and reduced inflammatory cytokine production (Fig. 5N). Collectively, these data establish DUSP10 as a potent negative regulator of ischemic injury.

### Rescue experiments confirm TRIM29 mediates kidney injury via regulation of DUSP10

**Fig. 6:**
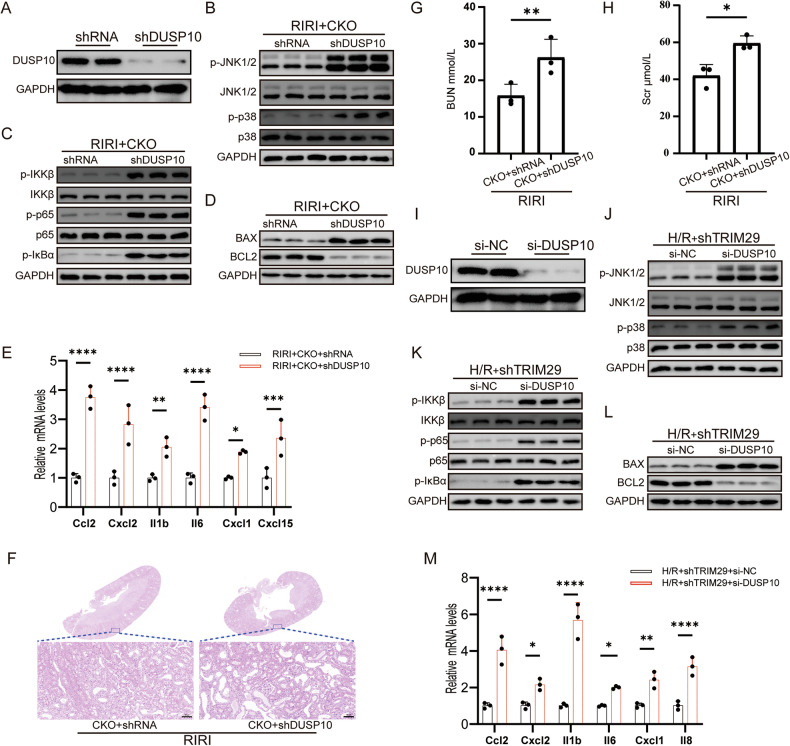
Rescue experiments confirm TRIM29 mediates kidney injury via regulation of DUSP10. **A** Western blot validation of DUSP10 knockdown efficiency in kidney tissues from TRIM29 CKO mice injected with AAV9-shNC or AAV9-shDUSP10. Western blot analysis of MAPK pathway components (**B**, p-JNK, JNK, p-p38, and p38), NF-κB pathway components (**C**, p-IKKβ, p-p65, and p-IκBα), and apoptosis markers (**D**, BAX and BCL-2) in kidney tissues. Mice were pre-treated with the indicated AAV9 vectors for 2 weeks prior to Sham or RIRI operation. **E** qRT-PCR analysis of inflammatory cytokines (*Ccl2, Cxcl2, Il1b, Il6*, and *Cxcl15*) in kidney tissues from TRIM29 CKO mice pre-treated with AAV9-shNC or AAV9-shDUSP10 followed by RIRI. **F** Representative H&E staining of kidney sections from the indicated groups. BUN (**G**) and Serum creatinine (**H**) levels in TRIM29 CKO mice subjected to AAV9 injection and subsequent RIRI. **I** Western blot validation of transient DUSP10 knockdown using small interfering RNA (siRNA) in HK-2 cells with stable TRIM29 knockdown(shTRIM2**9**). Western blot analysis of MAPK pathway (**J**), NF-κB pathway (**K**), and apoptosis markers (**L**) in shTRIM29 HK-2 cells transfected with si-NC or si-DUSP10 prior to H/R treatment. **M** qRT-PCR analysis of inflammatory cytokines (*Ccl2, Cxcl2, Il1b, Il6*, and *Il8*) in HK-2 cells from the indicated groups. Data are presented as mean ± SEM. Differences between two groups were determined using t-test. For comparisons involving two or more independent variables, two-way ANOVA was applied. *P < 0.05, **P < 0.01, ***P < 0.001, ****P < 0.0001. Each experiment was repeated at least three times independently.

To confirm the functional hierarchy of the TRIM29–DUSP10 axis, we performed rescue experiments. In TRIM29 CKO mice, AAV-mediated knockdown of DUSP10 abolished the nephroprotection conferred by TRIM29 deletion (Figs. 6A and S4A). Specifically, DUSP10 silencing reactivated JNK/p38 (Figs. 6B and S4B) and NF-κB signaling (Figs. 6C and S4C), restored pro-apoptotic marker expression (Figs. 6D and S4D), and re-elevated inflammatory cytokines (Fig. 6E). Consequently, the structural preservation and functional recovery observed in CKO mice were reversed (Figs. 6F–H and S4E). Consistent with in vivo findings, co-knockdown of DUSP10 in HK-2 cells negated the protective effects of TRIM29 silencing (Figs. 6I–M and S4F–I). Furthermore, pharmacological inhibition of JNK (SP600125) or p38 (SB203580) completely reversed NF-κB activation and apoptosis induced by DUSP10 deficiency (Fig. S4J–O), confirming that DUSP10 exacerbates downstream injury strictly via JNK/p38 hyperactivation. Collectively, these data provide compelling evidence that TRIM29 targets DUSP10 to trigger MAPK-dependent inflammatory and apoptotic cascades, thereby exacerbating renal injury.

### TRIM29 mediates DUSP10 ubiquitination via K48-linked chains primarily at lysine 231 and promotes its proteasomal degradation

**Fig. 7:**
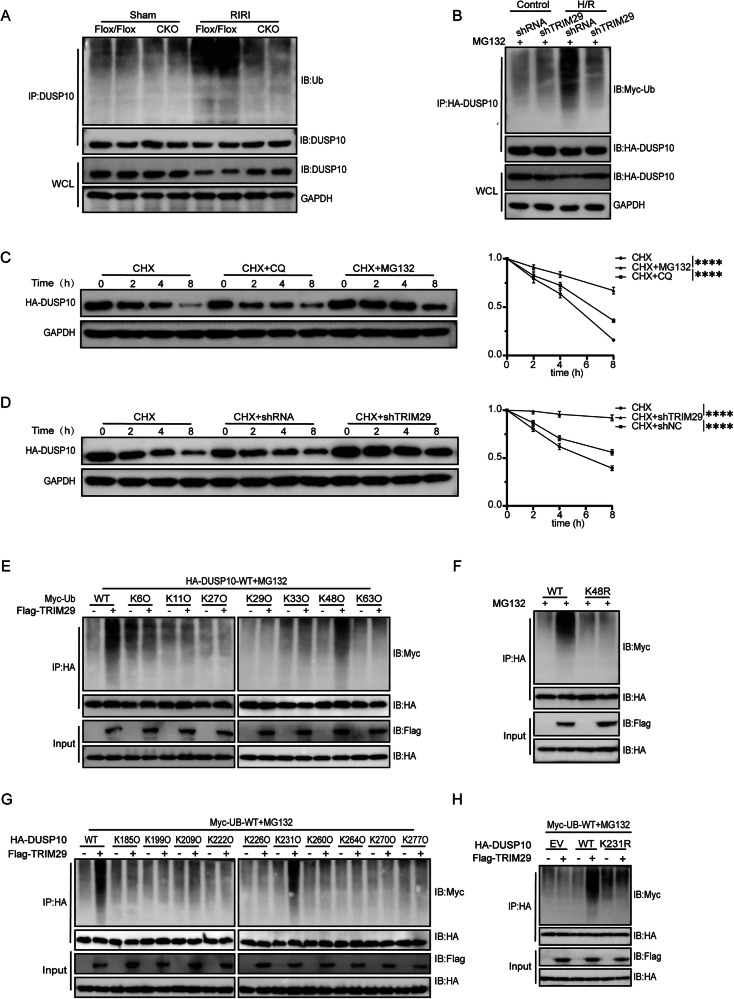
TRIM29 mediates DUSP10 ubiquitination via K48-linked chains primarily at lysine 231 and promotes its proteasomal degradation. **A** Co-immunoprecipitation (Co-IP) analysis of endogenous DUSP10 ubiquitination in kidney tissues from TRIM29 ^*Flox/Flox*^ and TRIM29 CKO mice subjected to Sham or RIRI operation. **B** Co-IP analysis of DUSP10 ubiquitination in HK-2 cells transfected with shNC or shTRIM29 under Control or H/R conditions in the presence of MG132 (10 μM, 6 h). **C** Western blot analysis of DUSP10 degradation in HK-2 cells treated with cycloheximide (CHX, 20 μg/mL) in the presence of the proteasome inhibitor MG132 or the autophagy inhibitor Chloroquine (CQ). **D** CHX chase assay analyzing DUSP10 protein half-life in HK-2 cells transfected with shNC or shTRIM29. **E** Linkage-specific ubiquitination assay in HEK293T cells treated with MG132 and co-transfected with Flag-TRIM29, HA-DUSP10, and Myc-Ub “Lysine-only” mutants retaining only a single lysine residue (e.g., K48O, K63O). **F** Ubiquitination assay using the “Lysine-dead” Myc-Ub-K48R mutant (incapable of forming K48 linkages) to validate linkage specificity in MG132-treated HEK293T cells. **G** Mapping of the ubiquitination site using HA-DUSP10 “Lysine-only” mutants (only the indicated lysine is retained, others are mutated to arginine) co-transfected with Flag-TRIM29 under MG132 treatment. **H** Validation of the critical ubiquitination site using the “Lysine-dead” DUSP10-K231R single point mutant. (Note: The suffix “O” denotes a “Lysine-only” mutant, whereas “R” indicates a “Lysine-dead” single lysine-to-arginine substitution.) Data are presented as mean ± SEM, and two-way ANOVA was applied. ****P < 0.0001. Each experiment was repeated at least three times independently.

To elucidate the precise molecular mechanism by which TRIM29 regulates DUSP10 protein stability, we first interrogated the ubiquitination status of DUSP10. In the in vivo model, Co-IP assays revealed that the ubiquitination level of endogenous DUSP10 was markedly potentiated in the injured kidneys of TRIM29 ^*Flox/Flox*^ mice following RIRI; however, this effect was significantly abrogated in TRIM29 CKO mice (Fig. 7A). Corroborating these findings in vitro, H/R stimulation enhanced DUSP10 ubiquitination in HK-2 cells treated with the proteasome inhibitor MG132, whereas shRNA-mediated knockdown of TRIM29 effectively suppressed this modification (Fig. 7B). Collectively, these data provide robust evidence that TRIM29 functions as a critical E3 ligase promoting DUSP10 ubiquitination under ischemic stress.

Next, we dissected the degradation pathway governing DUSP10 stability using cycloheximide (CHX) chase assays. DUSP10 underwent rapid turnover in control cells; however, this degradation was effectively halted by the proteasome inhibitor MG132, but not by the autophagy inhibitor chloroquine (CQ) (Fig. 7C, D), indicating that DUSP10 is degraded via the ubiquitin-proteasome system (UPS). Substantiating the destabilizing role of TRIM29, silencing TRIM29 significantly prolonged the half-life of DUSP10 protein during the CHX chase (Fig. 7E, F), confirming that TRIM29 is the upstream driver of DUSP10 proteasomal turnover.

To determine the specific topology of the ubiquitin chain mediated by TRIM29, we performed linkage-specific ubiquitination assays in the presence of MG132 using ubiquitin mutants retaining only a single lysine residue (e.g., K48O, K63O). The results demonstrated that TRIM29 robustly catalyzed the formation of K48-linked polyubiquitin chains on DUSP10, a canonical signal for proteasomal degradation, while other linkages showed minimal involvement (Fig. 7G). Conversely, when cells were transfected with a ubiquitin mutant incapable of forming K48 linkages (K48R), the TRIM29-induced ubiquitination of DUSP10 was completely abolished (Fig. 7H).

Finally, to pinpoint the specific amino acid residue serving as the ubiquitin acceptor, we generated a series of DUSP10 single-lysine mutants covering the binding interface (168–321 aa), in which only one specific lysine was retained while others were mutated to arginine (Fig. S5A). By co-transfecting Flag-TRIM29 with these mutants under MG132 treatment, we observed that the mutant retaining only Lysine 231 (K231O) exhibited the strongest polyubiquitination signal (Fig. 7I). To validate this site, we generated a point mutant by substituting Lysine 231 with Arginine (K231R). Immunoblotting confirmed that the K231R mutation effectively negated TRIM29-mediated ubiquitination (Fig. 7J). Notably, enzymatic assays confirmed that K231 ubiquitination triggers DUSP10 turnover without compromising its intrinsic phosphatase activity (Fig. S5B, C). Taken together, these findings establish that TRIM29 selectively targets DUSP10 for K48-linked polyubiquitination at K231, thereby driving its proteasomal degradation.

## Discussion

Ischemic insult is a well-established precipitant of acute kidney injury (AKI), driving tubular dysfunction and initiating maladaptive repair that often progresses toward chronic disease [1]. In this study, we identify TRIM29 as a stress-inducible E3 ubiquitin ligase that functions as a previously unrecognized and pivotal amplifier of renal ischemia-reperfusion injury. Through integrative transcriptomic, proteomic, and biochemical approaches, we demonstrate that TRIM29 directly engages the dual-specificity phosphatase DUSP10 and catalyzes its K48-linked ubiquitination at lysine 231, triggering proteasomal degradation. This post-translational destabilization of DUSP10 lifts a critical inhibitory constraint on the JNK/p38 MAPK axis, thereby sustaining pro-inflammatory and pro-apoptotic signaling during ischemic AKI. TRIM29 induction and DUSP10 depletion thus form a reciprocal regulatory loop that drives pathological MAPK hyperactivation and accelerates tubular injury.

These findings refine the emerging concept that TRIM family E3 ligases function as central determinants of renal stress adaptation. Our findings align with recent work by Chen et al., who identified TRIM65 as a mediator of mitochondrial dysfunction in AKI through VDAC1 ubiquitination [17], and with Li et al., who revealed that TRIM21 promotes AKI-to-CKD transition by impairing p62-mediated autophagy [18]. Yet TRIM29, despite its documented functions in cancer progression, innate immunity, and oxidative stress responses, has not previously been mechanistically linked to renal pathology [20, 21]. The strong ischemia-selective induction of TRIM29 observed here, together with the marked renoprotection afforded by its genetic ablation, reveals a fundamental and kidney-specific role for TRIM29 as a metabolic and inflammatory stress-responsive E3 ligase.

Mechanistically, our work also clarifies a long-standing question in AKI biology: how post-ischemic kidneys acquire persistent activation of the JNK/p38 MAPK axis despite intact upstream kinase circuitry. While MAPK signaling dynamics are governed by a balance between phosphorylation and phosphatase-mediated dephosphorylation [6], the regulatory fate of MAPK-directed phosphatases during AKI has remained poorly understood. Previous studies have linked E3 ligases like TRIM31 to fibrosis via upstream kinase regulation [22]. Not coincidentally, renal fibrosis occurs predominantly in patients diagnosed with AKI [23]. Here, LC–MS-based interactome screening identified DUSP10 as a high-confidence TRIM29 interactor. DUSP10, also known as MKP5, dephosphorylates JNK and p38, thus dampening MAPK-driven inflammatory responses [24]. Our observation that DUSP10 protein, but not transcript, is markedly reduced in ischemic kidneys provides direct evidence for post-translational erosion of phosphatase activity. We show that TRIM29 is the principal E3 ligase responsible for this loss, as its deletion restores DUSP10 stability while suppressing MAPK hyperactivation. While Wang et al. recently reported that TRIM29 destabilizes PERK via SUMOylation in viral myocarditis [25], our study defines a distinct, ubiquitin-dependent mechanism in the kidney, wherein TRIM29 directly ubiquitinates DUSP10 at K231, targeting it for proteasomal degradation. Importantly, the reno-protective effects of TRIM29 loss are largely negated by DUSP10 knockdown, establishing DUSP10 as the primary effector through which TRIM29 imposes MAPK-driven injury programs.

Beyond the linear TRIM29–DUSP10–MAPK axis, TRIM29 likely functions as a broader “stress integrator” within the ischemic microenvironment. Unlike typical TRIM family members, the TRIM29 N-terminus lacks a RING domain, featuring instead a unique architecture composed of dual zinc-finger B-boxes and a coiled-coil leucine zipper [26]. This structural configuration renders it capable of scaffolding multi-protein complexes under metabolic and oxidative stress [27]. Given its role as a sensor of DNA damage and redox imbalance in oncology [25, 28], it is plausible that ischemic injury triggers conserved upstream modules to mobilize TRIM29, thereby positioning it at the nexus of cell survival and maladaptive repair. Furthermore, the kidney-specific substrate preference toward DUSP10 observed here suggests that ischemia may reshape TRIM29’s interactome, potentially influencing additional pathways such as NF-κB signaling, apoptotic checkpoints, metabolic rewiring, or early pro-fibrotic transitions [29]. Persistent MAPK activation is a hallmark of maladaptive repair and a driver of AKI progression; our findings, therefore, position TRIM29 and DUSP10 at a critical inflection point in determining long-term renal outcomes after ischemic injury.

Clinically, the identification of TRIM29 as an inducible and pathogenic E3 ligase offers actionable therapeutic insight. Crucially, our observation that renal tubule-specific *Trim29* knockout mice are viable, healthy, and maintain normal baseline renal function and histology strongly indicates that basal TRIM29 is largely dispensable for physiological renal homeostasis. TRIM29 exhibits low baseline expression but is rapidly and robustly upregulated under ischemic stress, suggesting a wide therapeutic window with minimal interference in physiological renal homeostasis. Targeting the TRIM29–DUSP10 interface, either through competitive inhibitors, E3 ligase modulators, or PROTAC-based degraders, may represent a strategy to dampen MAPK hyperactivation without fully suppressing MAPK function, which is essential for tissue repair. Moreover, the high degree of evolutionary conservation in TRIM29 structure suggests that such interventions may possess favorable translatability [19].

This study has several implications for future work. First, while our current study identifies the transcription factor KLF10 as a primary upstream driver of TRIM29 induction during ischemia, the specific microenvironmental cues that initially trigger this cascade—such as redox stress [30], DNA damage pathways [31], mitochondrial dysfunction [32], or innate immune receptors—serve as the major trigger for this cascade could reveal additional therapeutic leverage points [33]. Second, our findings raise the possibility that TRIM29 may control a broader substrate landscape during AKI beyond DUSP10. Defining this interactome under dynamic ischemic conditions will be essential for understanding the full breadth of TRIM29’s influence. Finally, whether TRIM29 contributes to long-term fibrotic remodeling through persistent MAPK activation warrants investigation, particularly given the strong epidemiological link between AKI and CKD progression.

## Conclusions

In summary, we delineate a TRIM29–DUSP10–JNK/p38 MAPK signaling axis as a central mechanism underlying ischemia-induced renal injury. By establishing TRIM29 as a stress-responsive E3 ligase that destabilizes a key MAPK phosphatase, our work reshapes current understanding of post-ischemic signaling dysregulation and identifies TRIM29 as a compelling therapeutic target for mitigating AKI severity and potentially preventing its transition to chronic kidney disease.

## Materials and methods

### Human clinical samples

Human kidney tissue specimens, including samples from patients clinically diagnosed with acute kidney injury (AKI) (*n* = 5) and normal kidney tissues from control subjects (*n* = 5), were collected at The First Affiliated Hospital of Zhengzhou University. All clinical sample collection and experimental procedures were strictly conducted in accordance with the ethical principles formulated in the Declaration of Helsinki. The study protocol was reviewed and approved by the Clinical Research Ethics Committee of The First Affiliated Hospital of Zhengzhou University (Approval No.: 2026-KY-0495-001). Written informed consent was obtained from all patients or their authorized legal representatives prior to specimen collection. Upon acquisition, the tissue samples were immediately fixed in 4% paraformaldehyde, embedded in paraffin, and continuously sectioned for subsequent immunohistochemical (IHC) evaluation to assess the expression and localization of TRIM29 and DUSP10.

### Animal experiments

Wild-type C57BL/6J mice were purchased from the Experimental Animal Center of Zhengzhou University. TRIM29 ^*Flox/Flox*^ mice were acquired from Cyagen (Strain: S-CKO-19209, Guangzhou, China). Renal tubular-specific TRIM29 knockout mice (TRIM29 CKO) were generated by crossing TRIM29 ^*Flox/Flox*^ mice with Cdh16-Cre mice (C57BL/6J background, provided by Prof. Xiuheng Liu, Renmin Hospital of Wuhan University). Male mice aged 8–10 weeks were selected to establish all animal models and randomly allocated to the different experimental groups. The RIRI model was established by right nephrectomy and left renal pedicle clamping. Under isoflurane anesthesia, the left renal pedicle was clamped for 30 min to induce ischemia, followed by clamp removal to initiate reperfusion. Sham-operated mice underwent the same procedure without clamping. Tissues and blood were harvested 24 h post-reperfusion. During the outcome assessment and data analysis, investigators were blinded to the group allocation. All experiments were approved by the Animal Ethics Committee of The First Affiliated Hospital of Zhengzhou University.

### Cell culture and hypoxia/reoxygenation (H/R) model

HK-2 human proximal tubular cells (American Type Culture Collection, ATCC) were cultured in DMEM/F12 supplemented with 10% FBS. HEK293T cells were maintained in DMEM with 10% FBS. To simulate RIRI in vitro, cells were subjected to a hypoxia/reoxygenation (H/R) treatment, which consisted of hypoxia (1% O₂, 94% N₂, 5% CO₂) in glucose-free and serum-free medium for 12 h, followed by reoxygenation in complete medium for 2, 4, or 8 h. Reagents used included MG132 (Proteasome inhibitor, MedChemExpress [MCE], NJ, USA, HY-13259), Chloroquine (CQ, Autophagy inhibitor, MCE, HY-17589A), and Cycloheximide (CHX, Protein synthesis inhibitor, MCE, HY-12320), SP600125 (JNK inhibitor, MCE, HY-12041), and SB203580 (p38 MAPK inhibitor, MCE, HY-10256). For CHX chase assays, cells were treated with CHX (20 μg/mL) for 0, 2, 4, and 8 h to assess protein stability degradation kinetics.

### Statistical analysis

Data were analyzed using GraphPad Prism 9.5 software and are presented as mean ± standard error of the mean (SEM). Independent experiments were repeated at least three times. The “*n*” values in all statistical analyses and figure legends strictly represent the number of independent biological replicates (e.g., individual mice or distinct cell culture preparations). Where technical replicates were performed, their values were averaged to yield a single data point per biological sample. Individual data points are overlaid on bar graphs to illustrate the data distribution. Differences between two groups were determined using Student’s unpaired t-test. For comparisons among multiple groups, one-way ANOVA followed by Tukey’s post-hoc test (single variable) or two-way ANOVA followed by Bonferroni’s post-hoc test (two independent variables) was applied. A *P* value less than 0.05 was considered statistically significant.

## Supplementary information

Supplementary Methods, Figures, and Tables.

Original WB Images

## Data Availability

The RNA sequencing data generated in this study have been deposited in the NCBI Sequence Read Archive (SRA) database under accession code PRJNA1454392. The ubiquitinomics (mass spectrometry) data have been deposited in the iProX partner repository with the dataset identifier IPX0016670000. All other datasets supporting the findings of this study are available within the article and its Supplementary Materials. Additional raw data are available from the corresponding author upon reasonable request.
