# Supplementary material for: The E3 ligase TRIM29 drives renal ischemia-reperfusion injury by targeting DUSP10 for proteasomal degradation
Source: Cell Death Dis. 2026 Jun 13;17(1):742. doi: 10.1038/s41419-026-08990-w (PMC13487226; doi:10.1038/s41419-026-08990-w)

Fig 1B

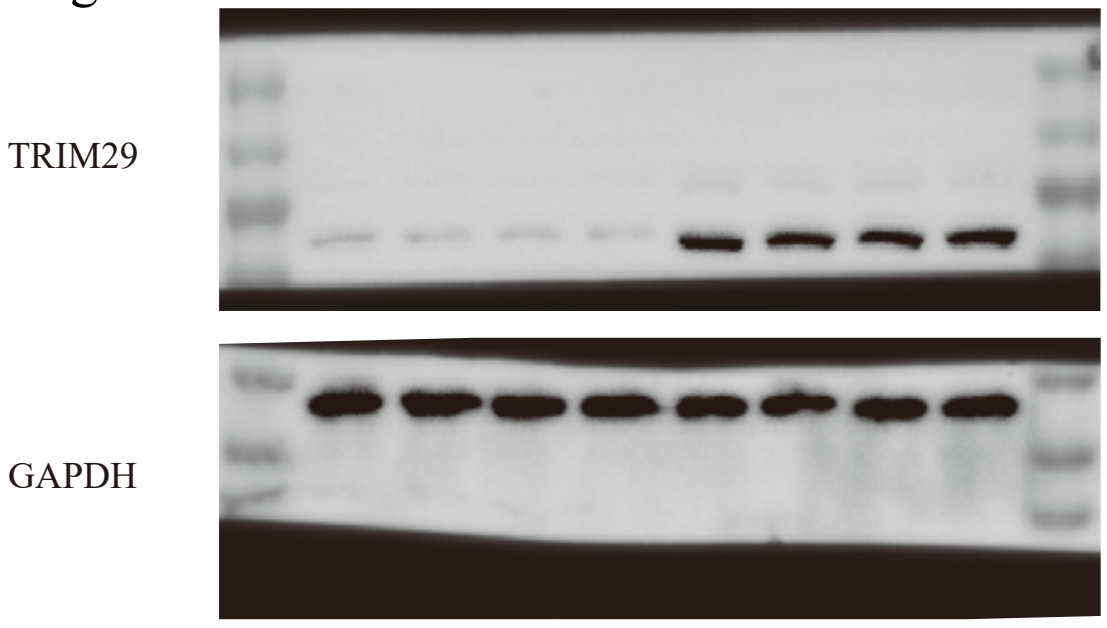

Fig 1C

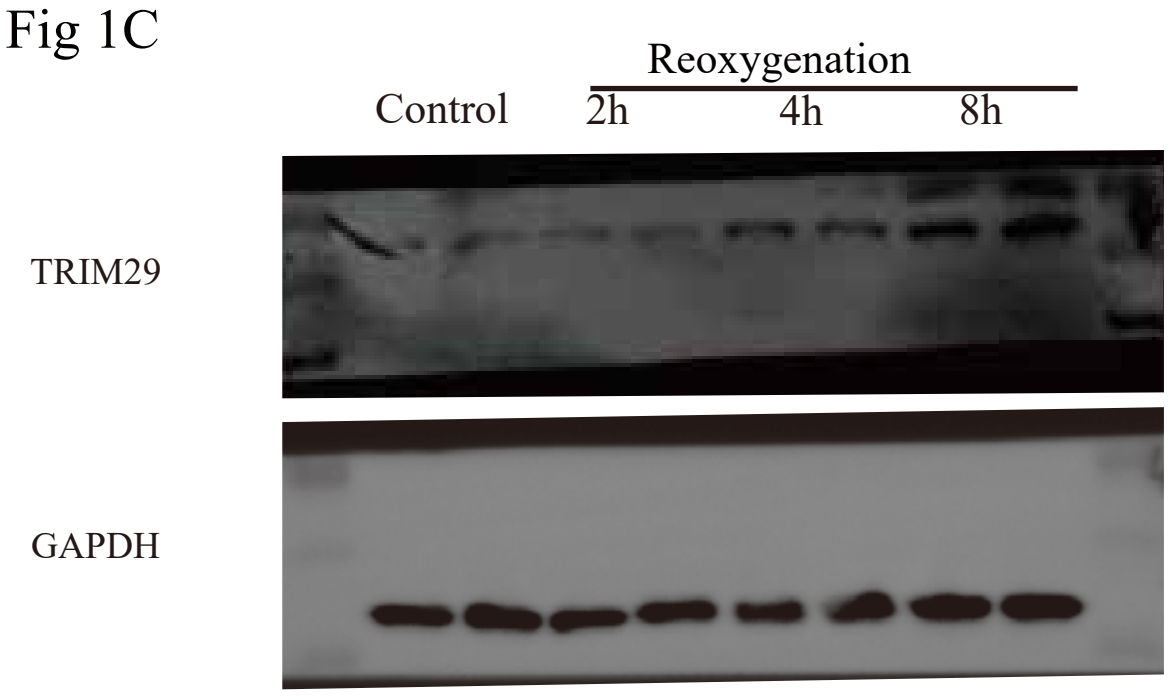

Fig 2G

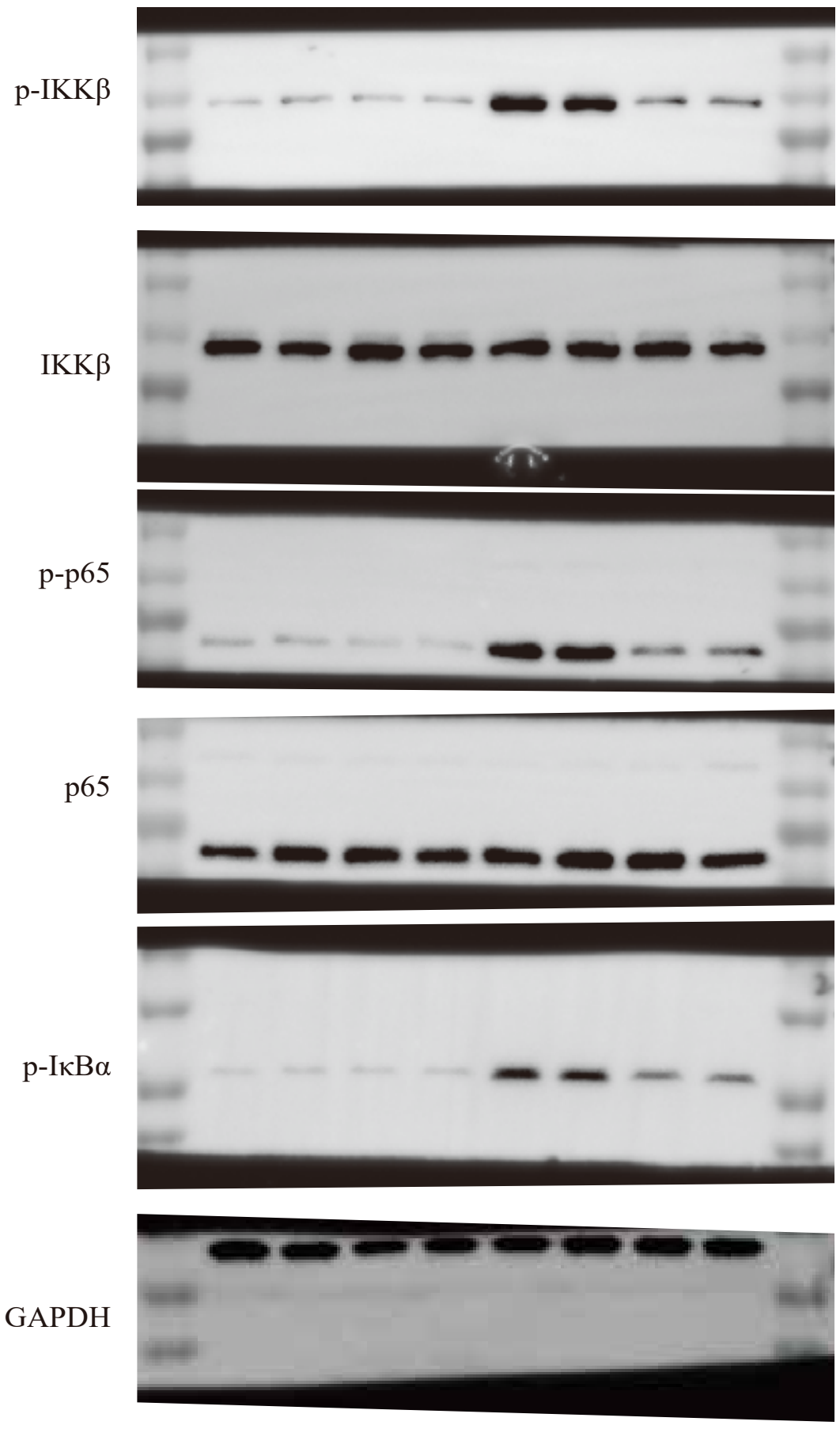

Fig 2H

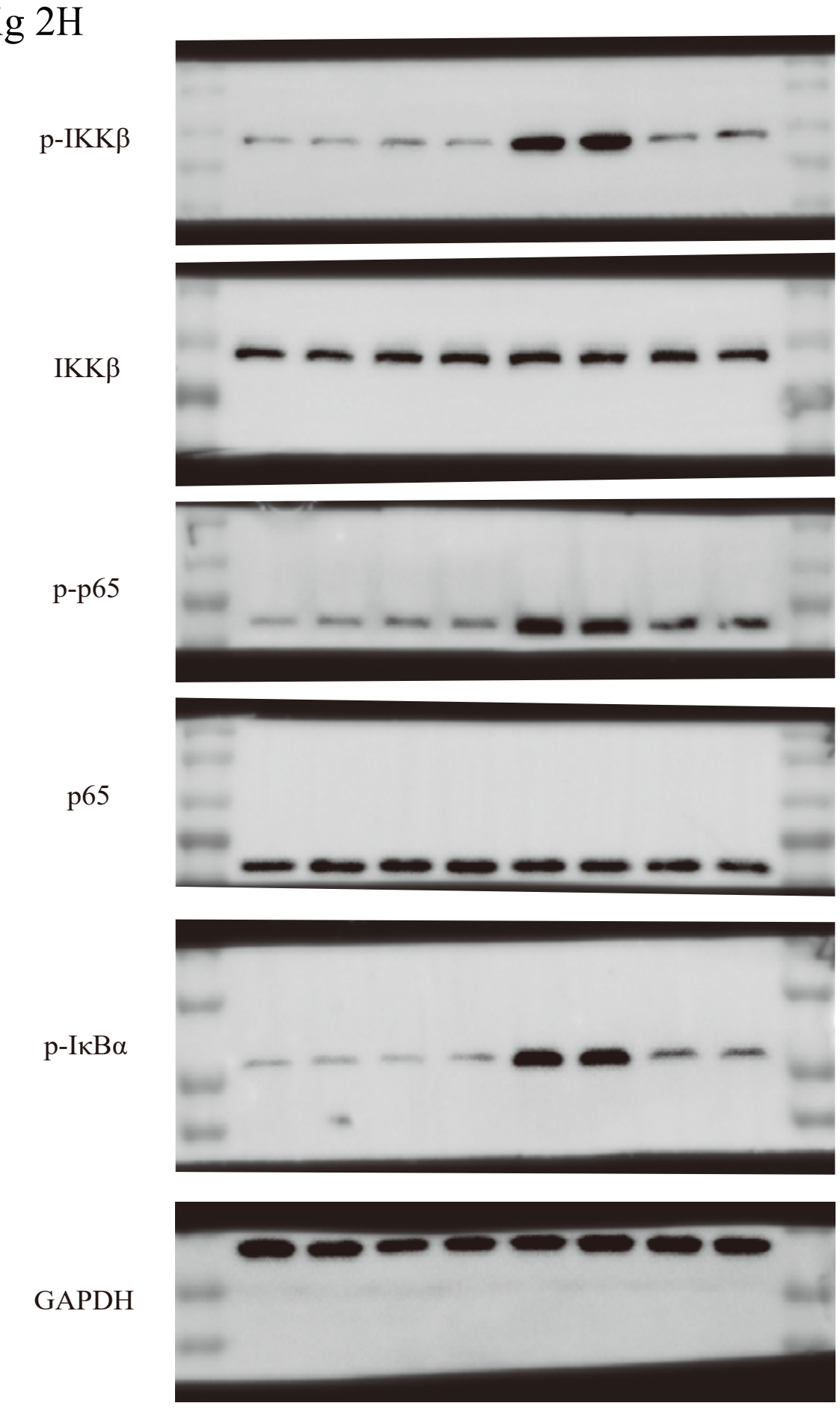

Fig 2J

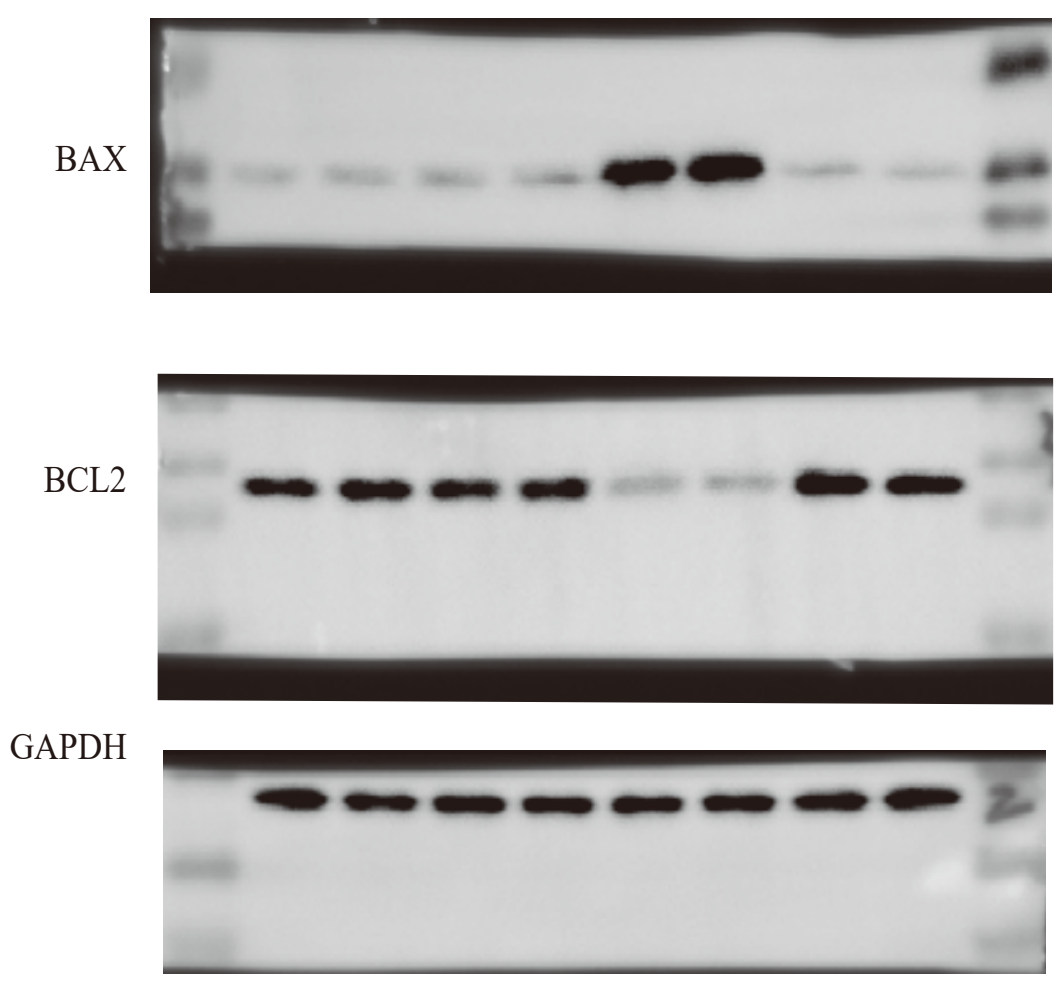

Fig 2K

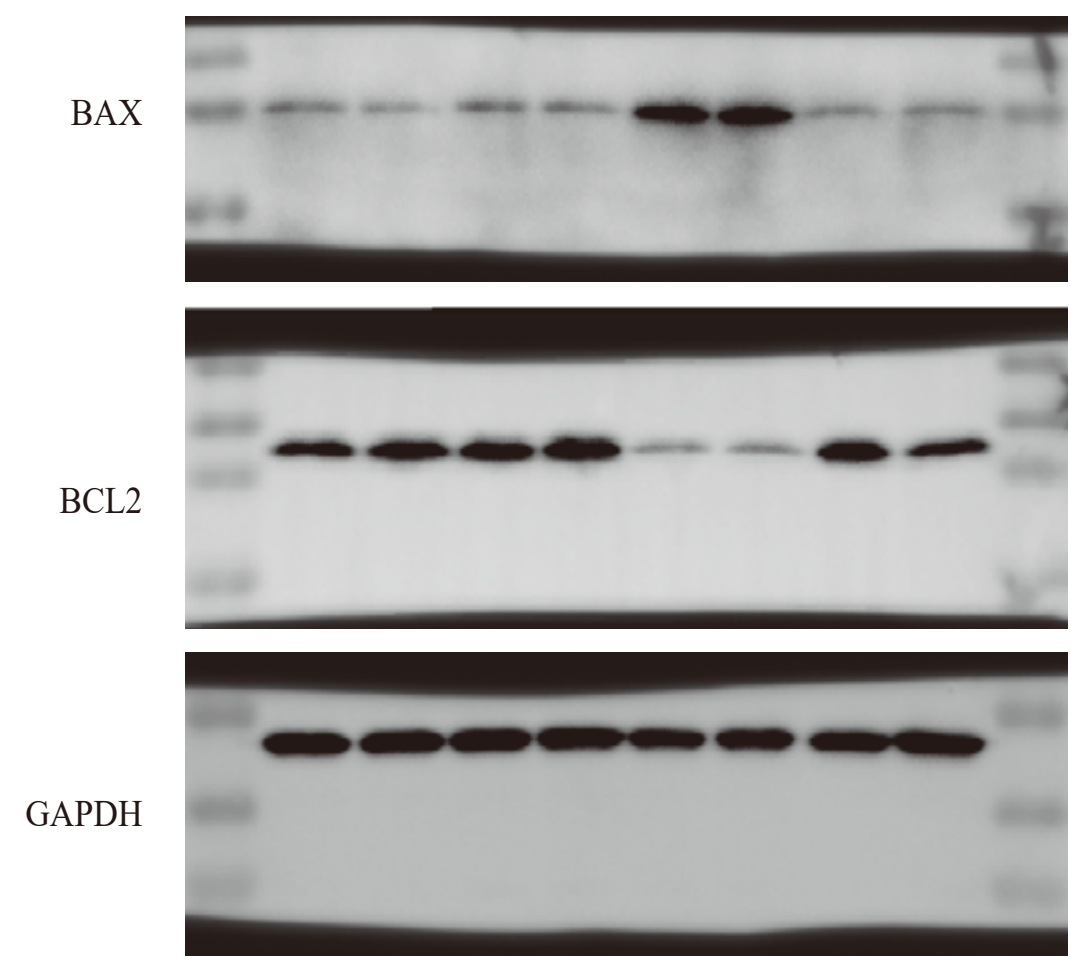

Fig 3F

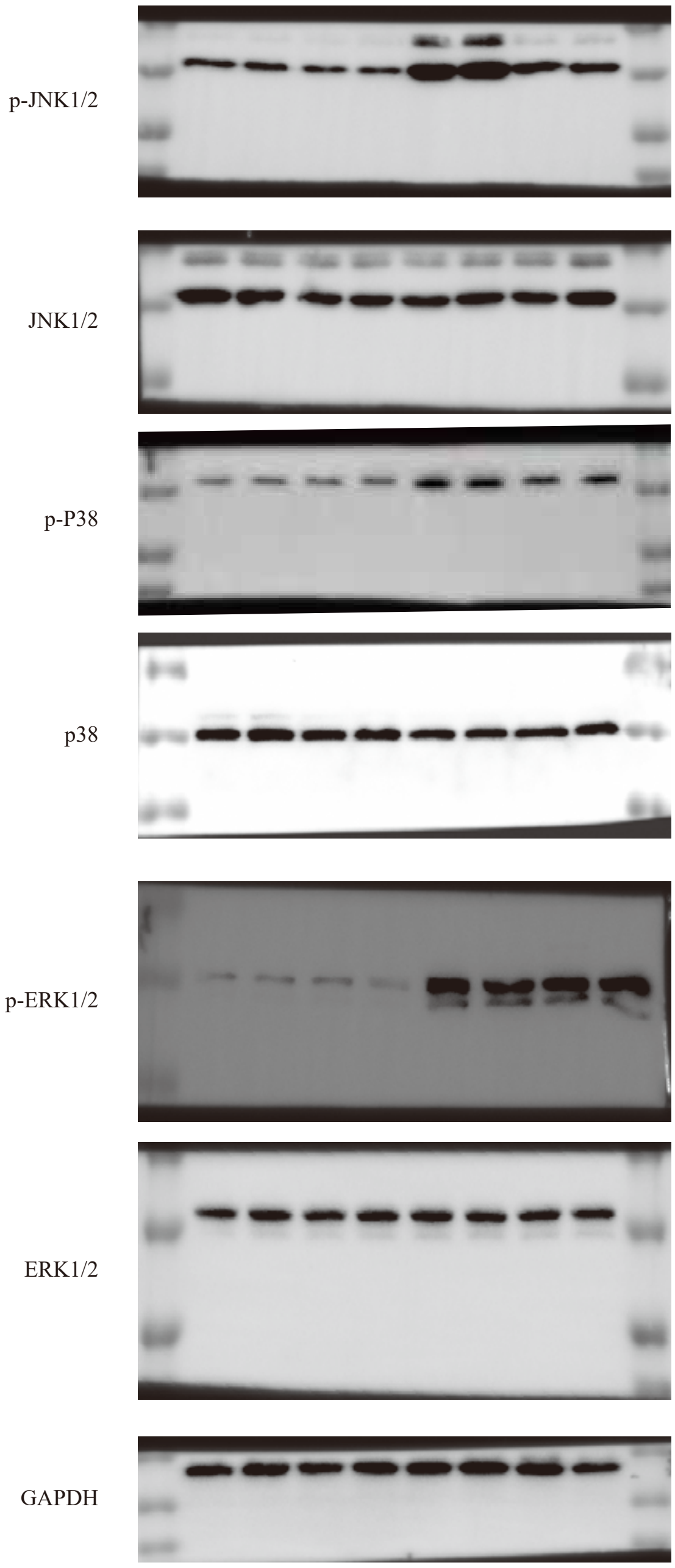

Fig 3G

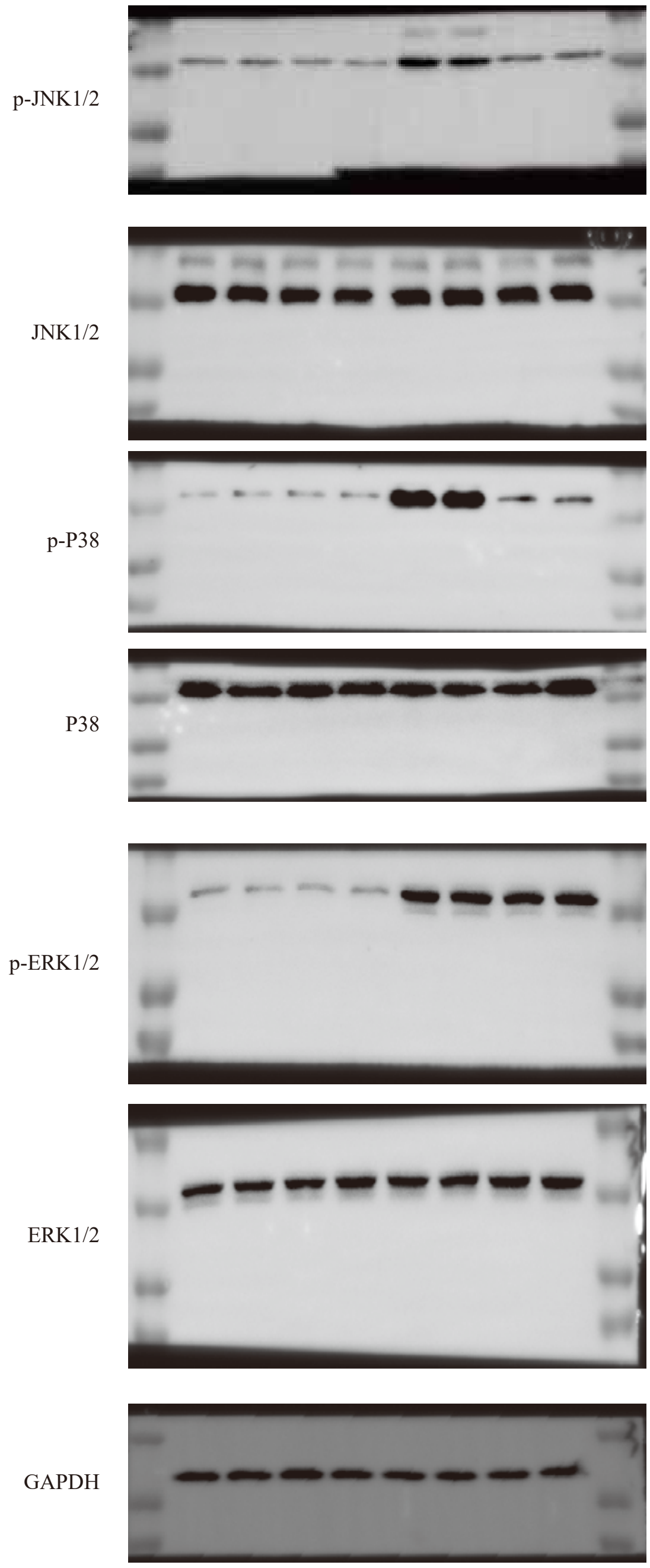

Fig 4C

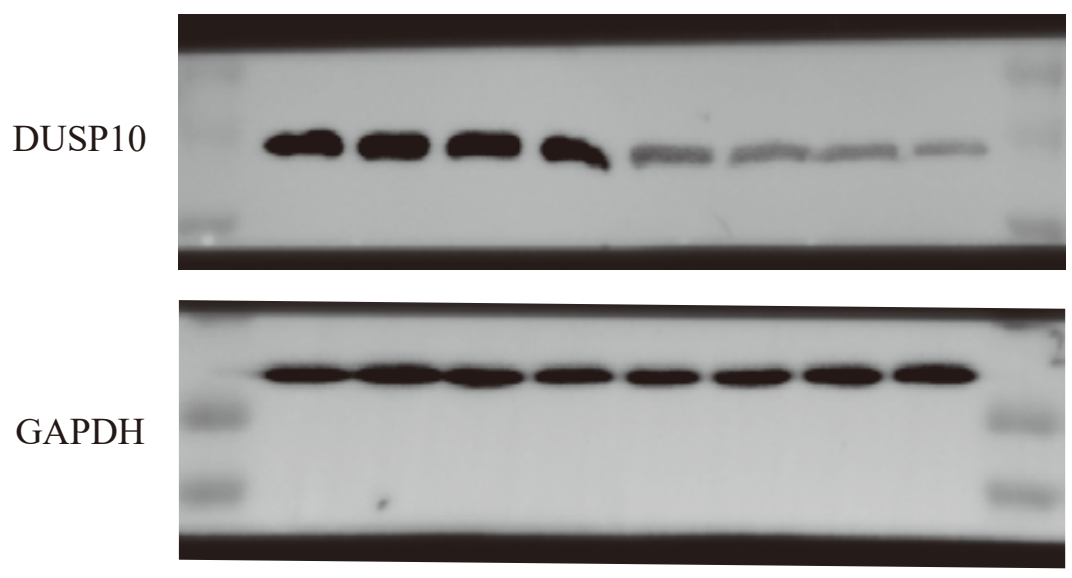

Fig 4D

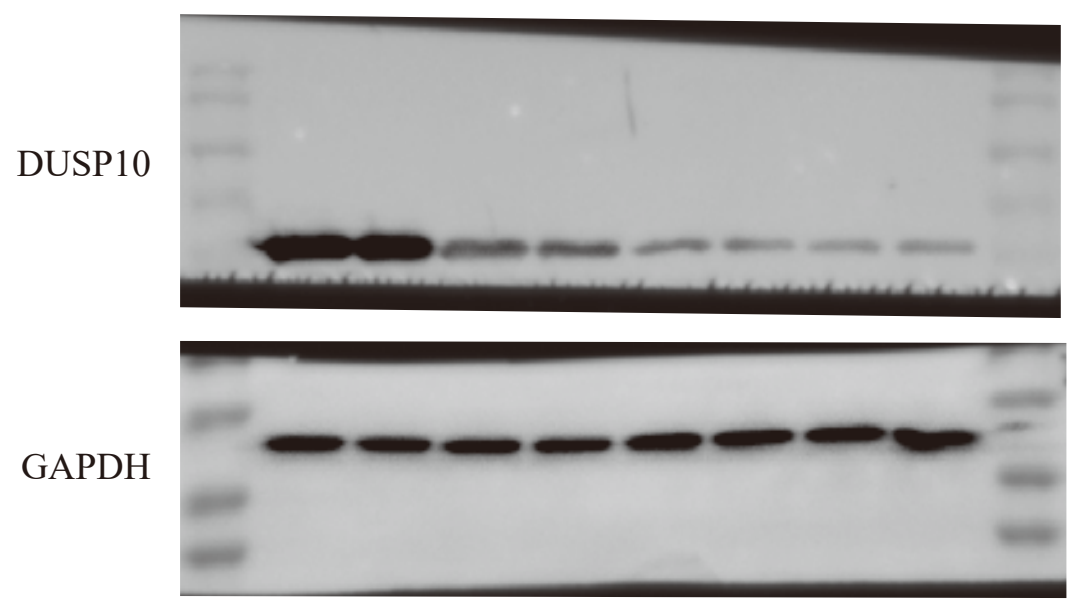

Fig 4F

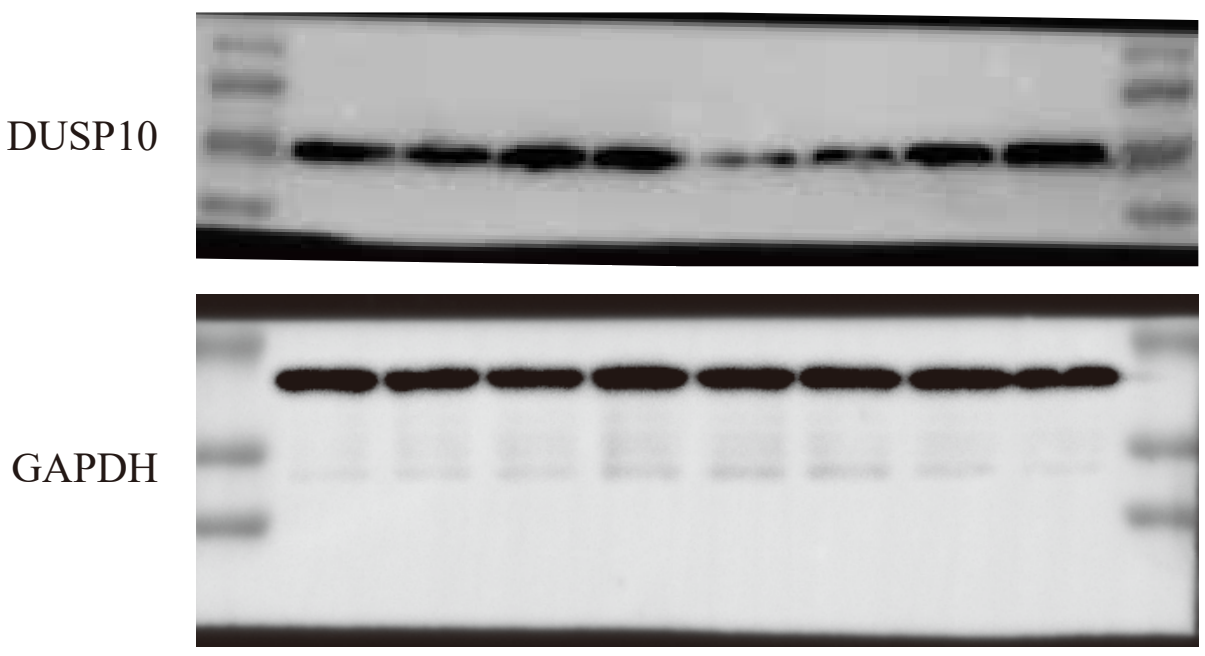

Fig 4G

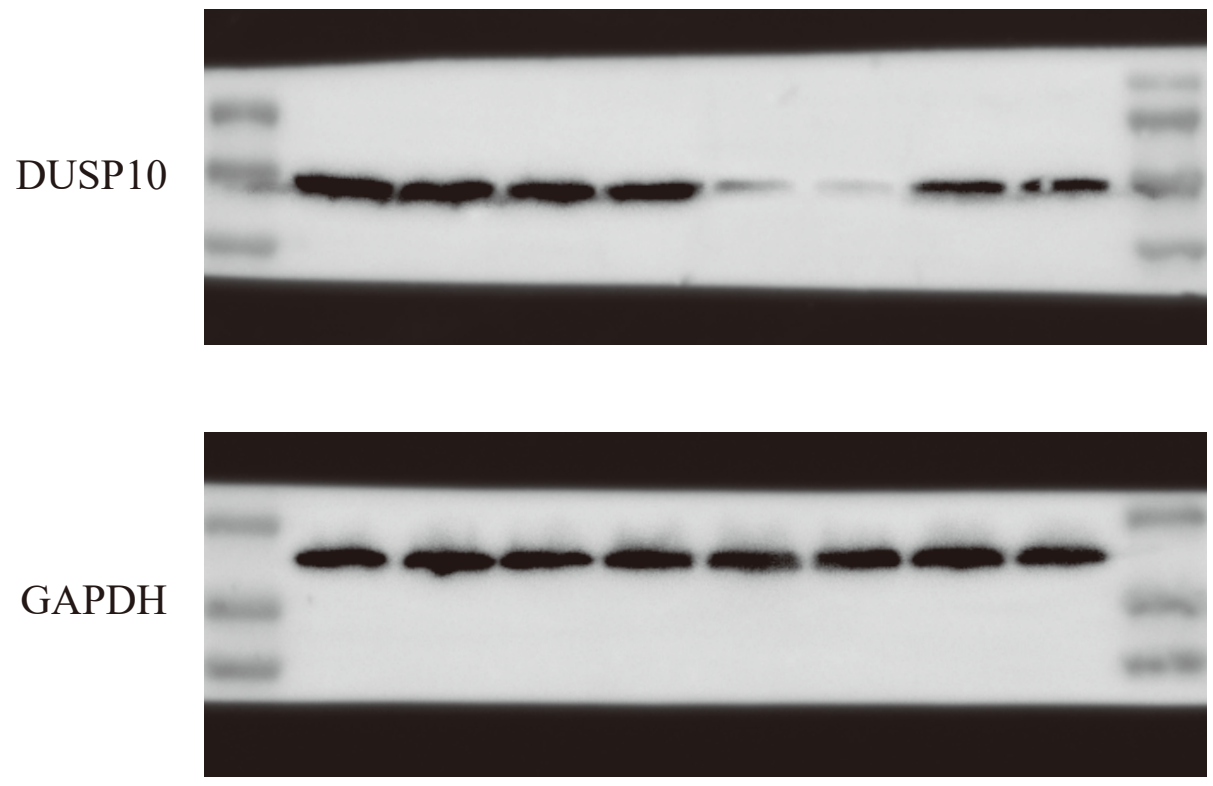

Fig 4H

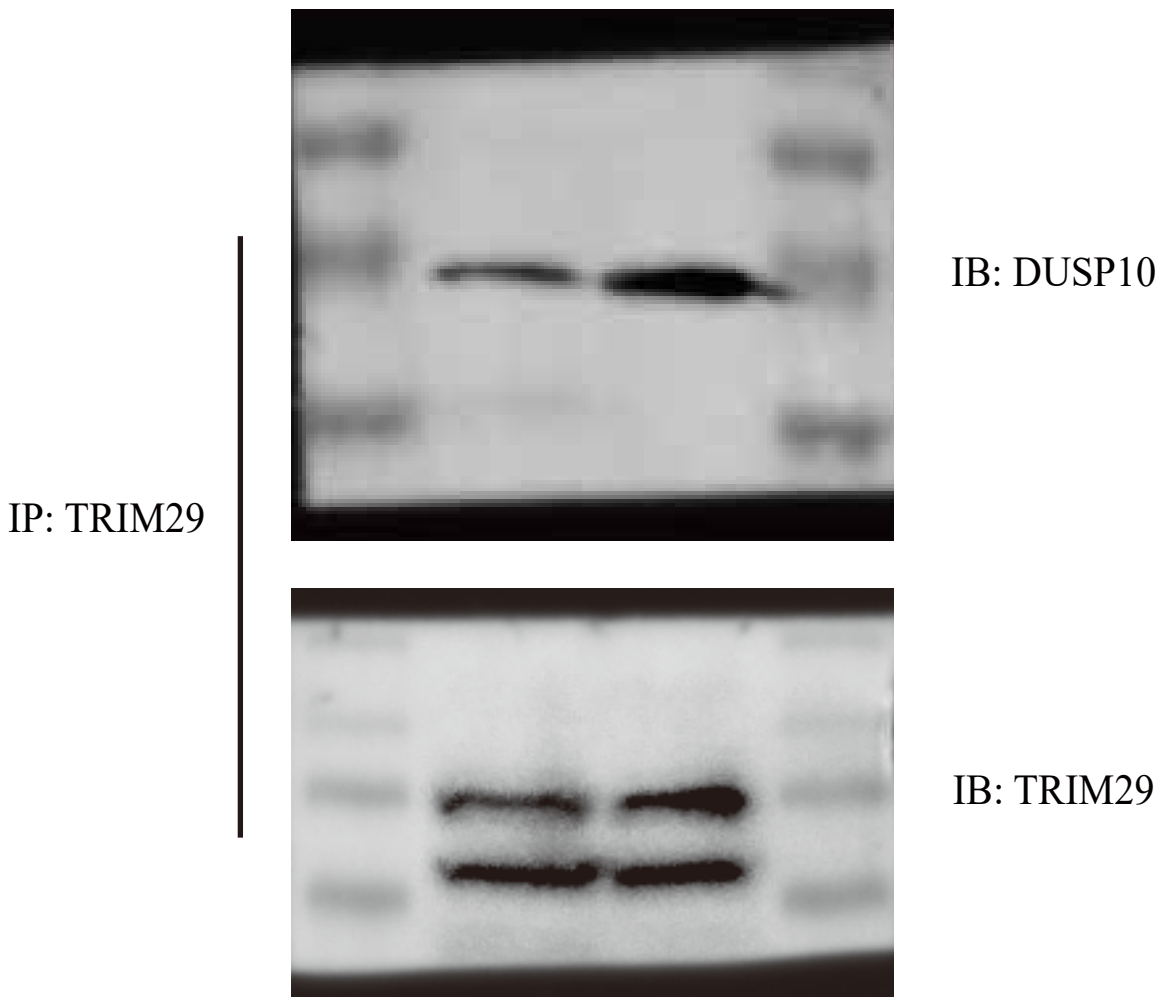

Fig 4I

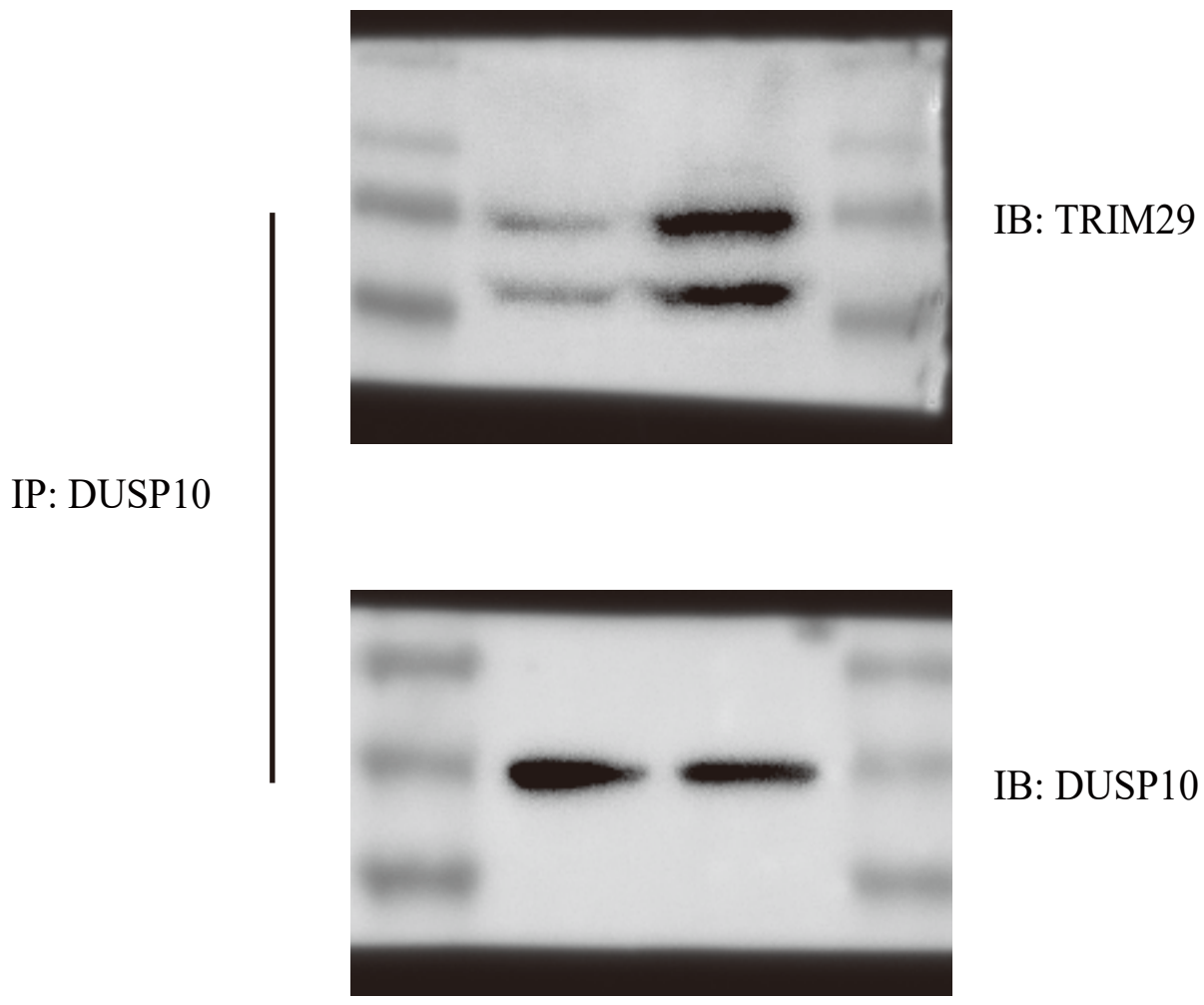

Fig 4L

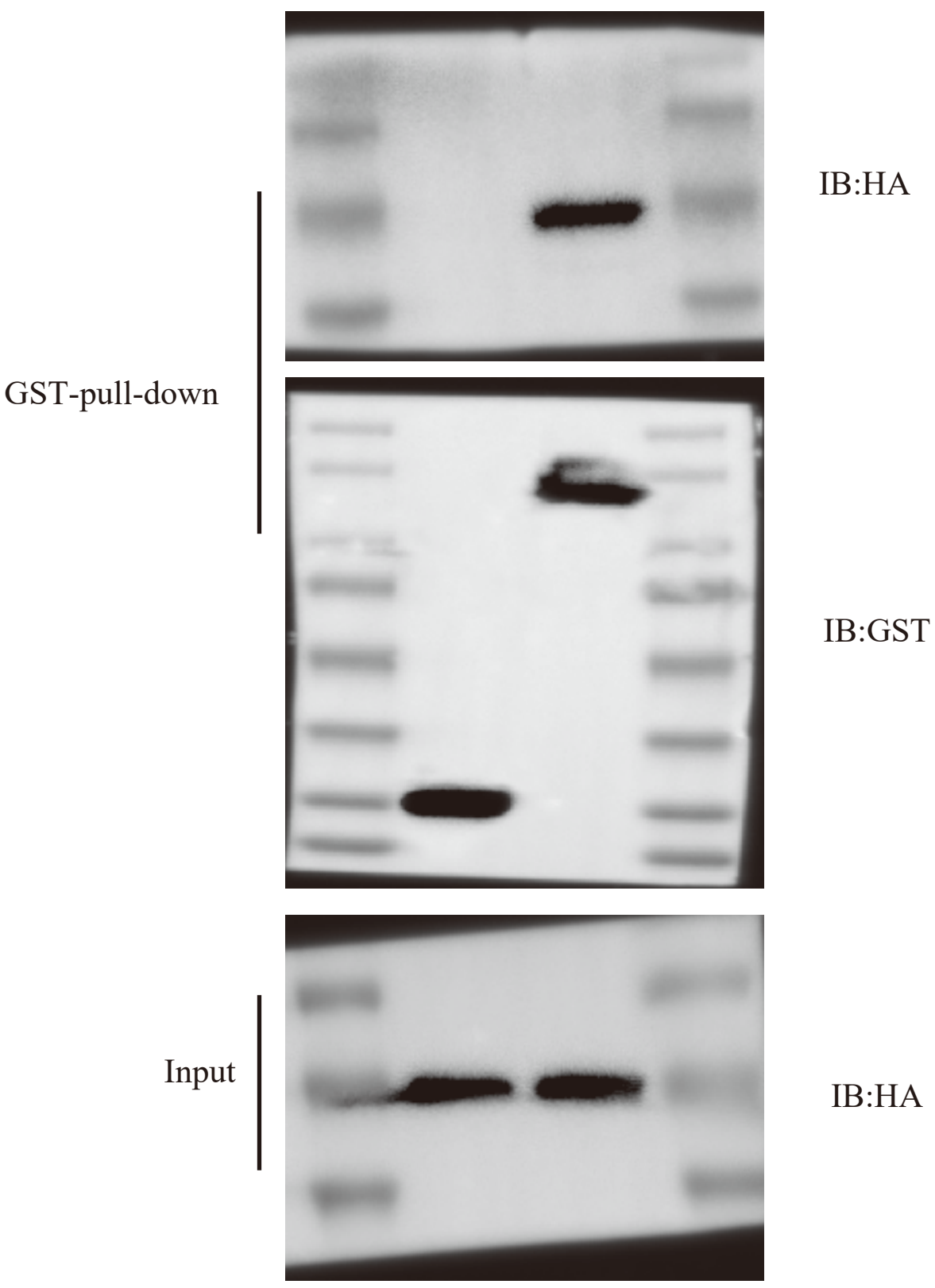

Fig 4M

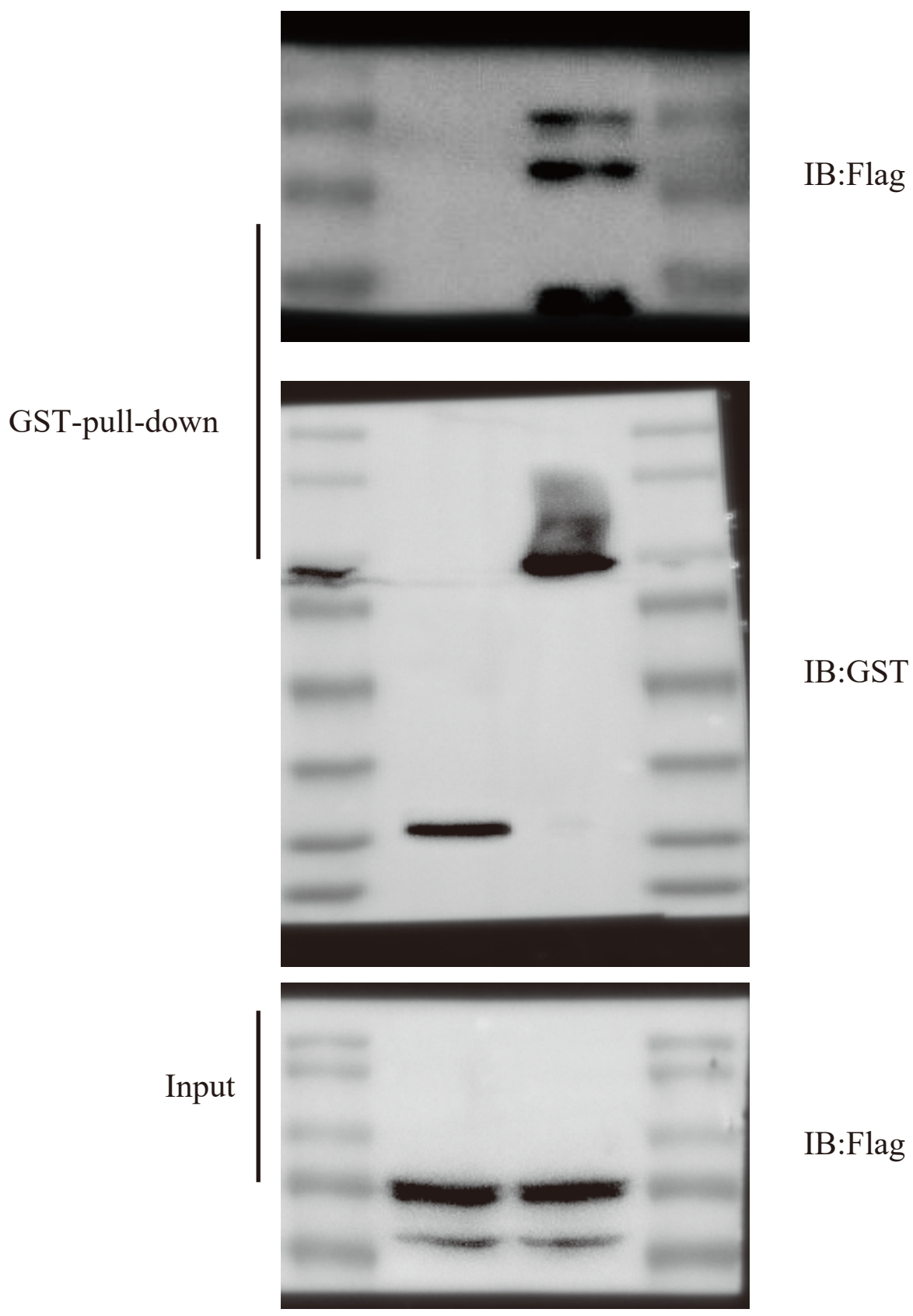

Fig 4P

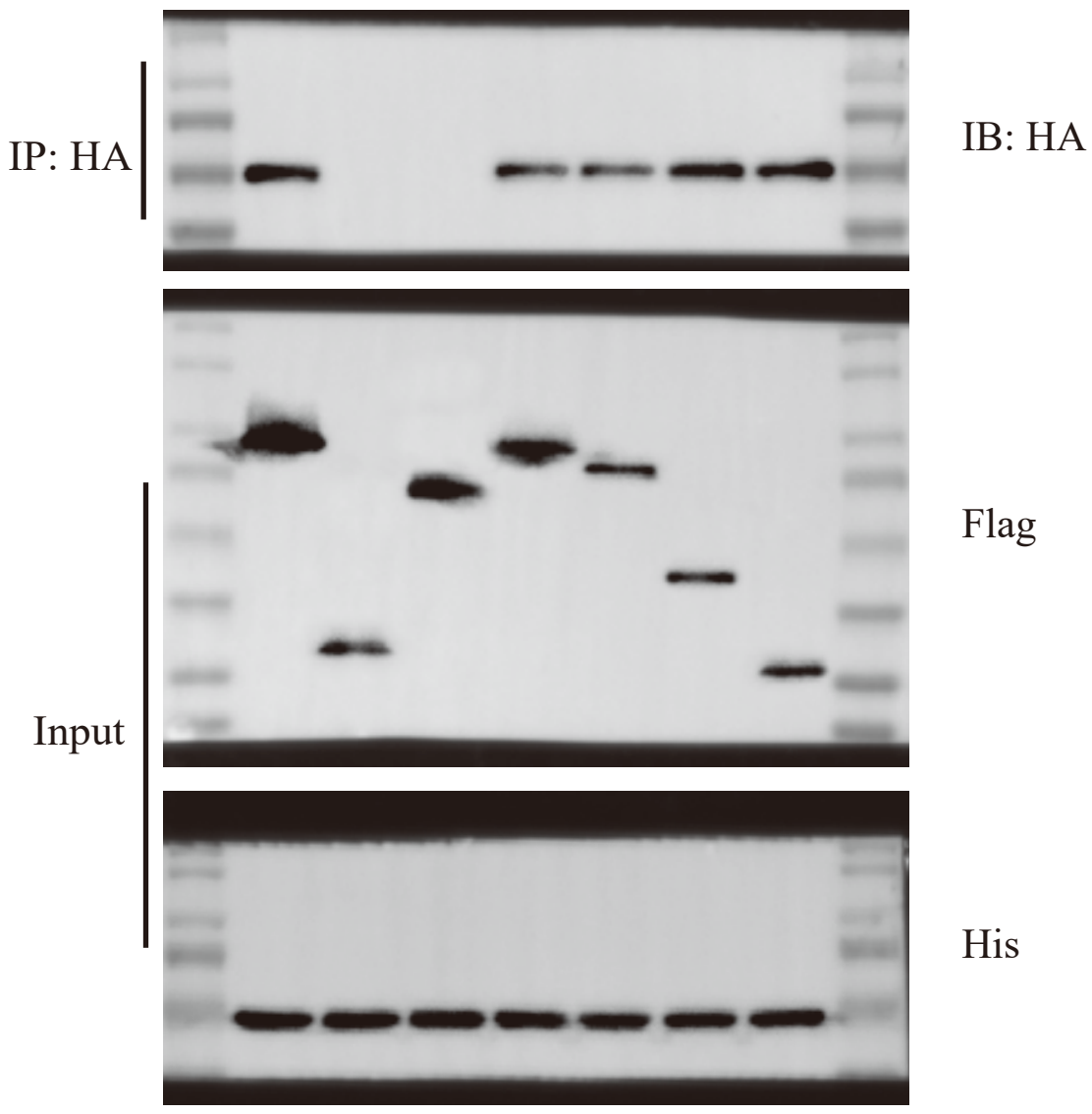

Fig 4Q

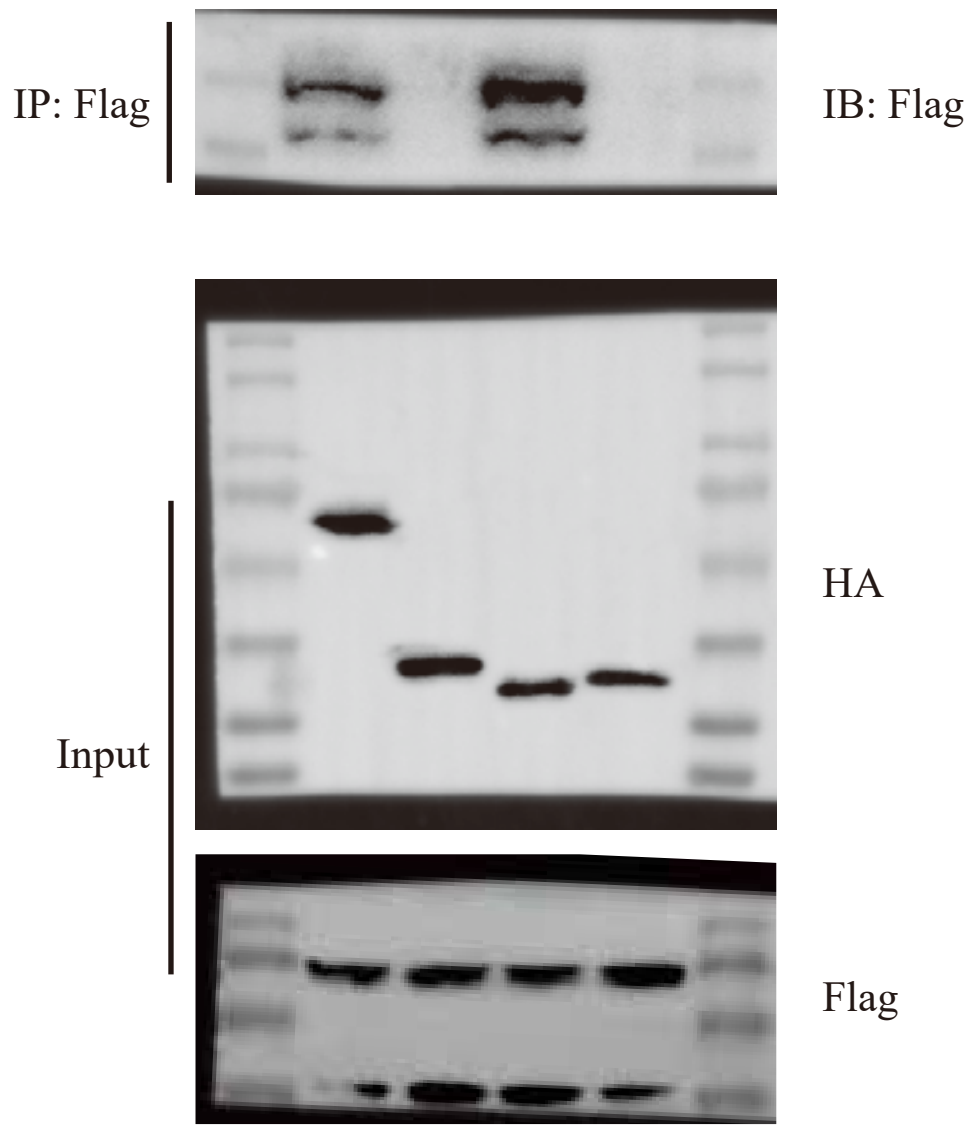

Fig 5B

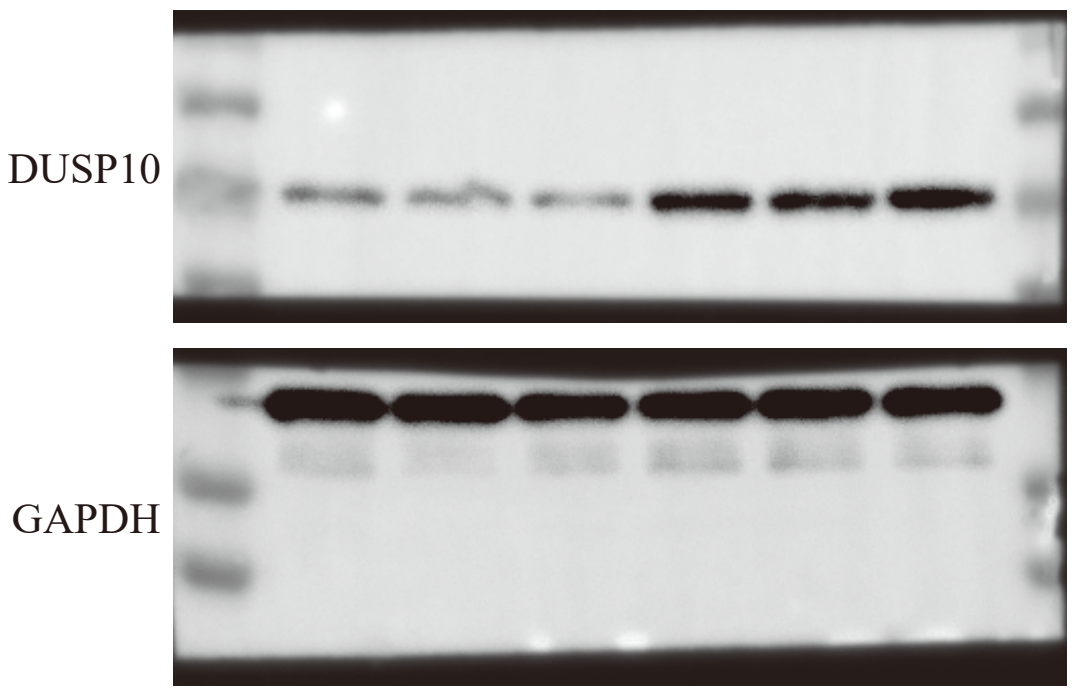

Fig 5C

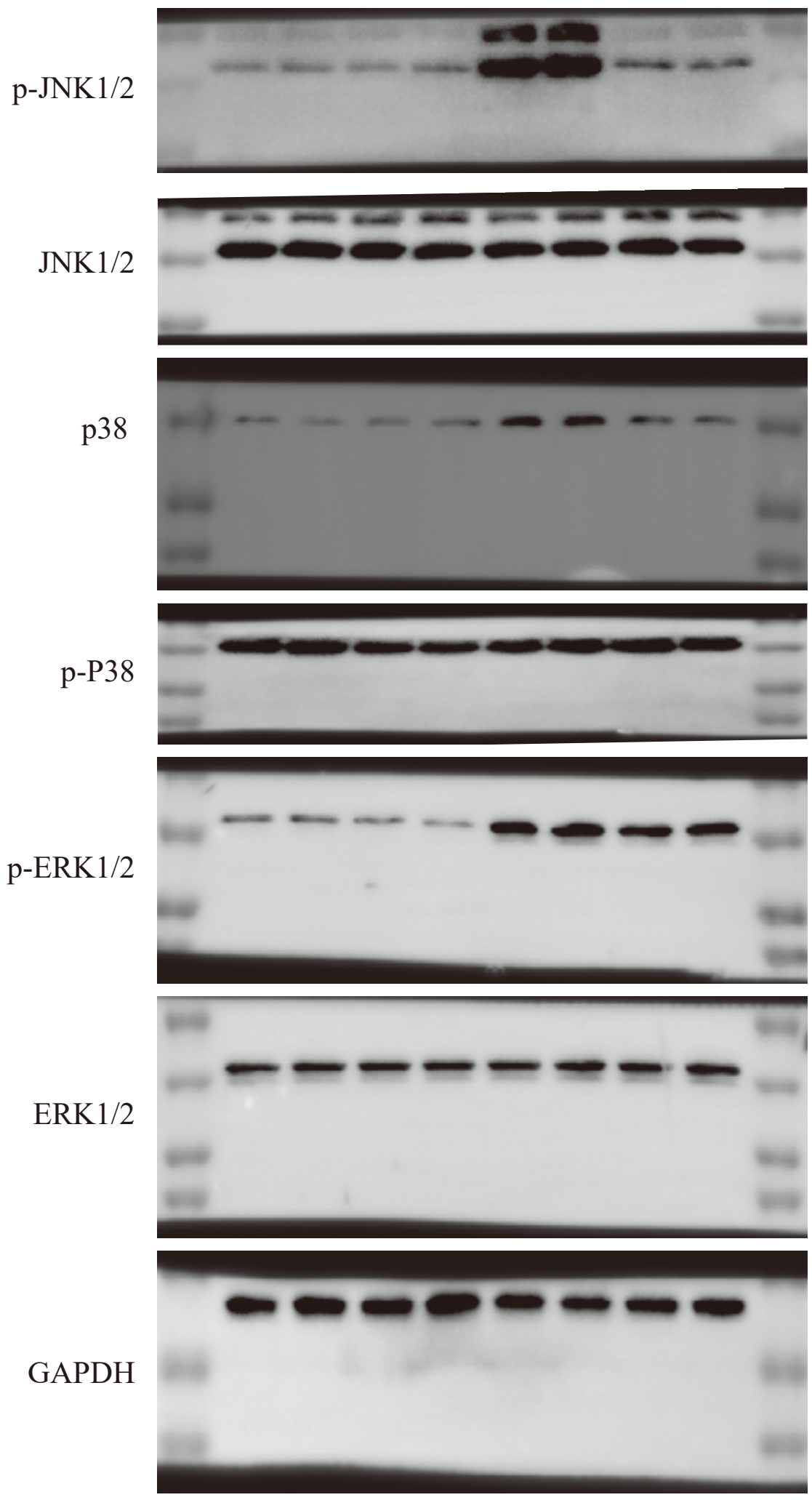

Fig 5D

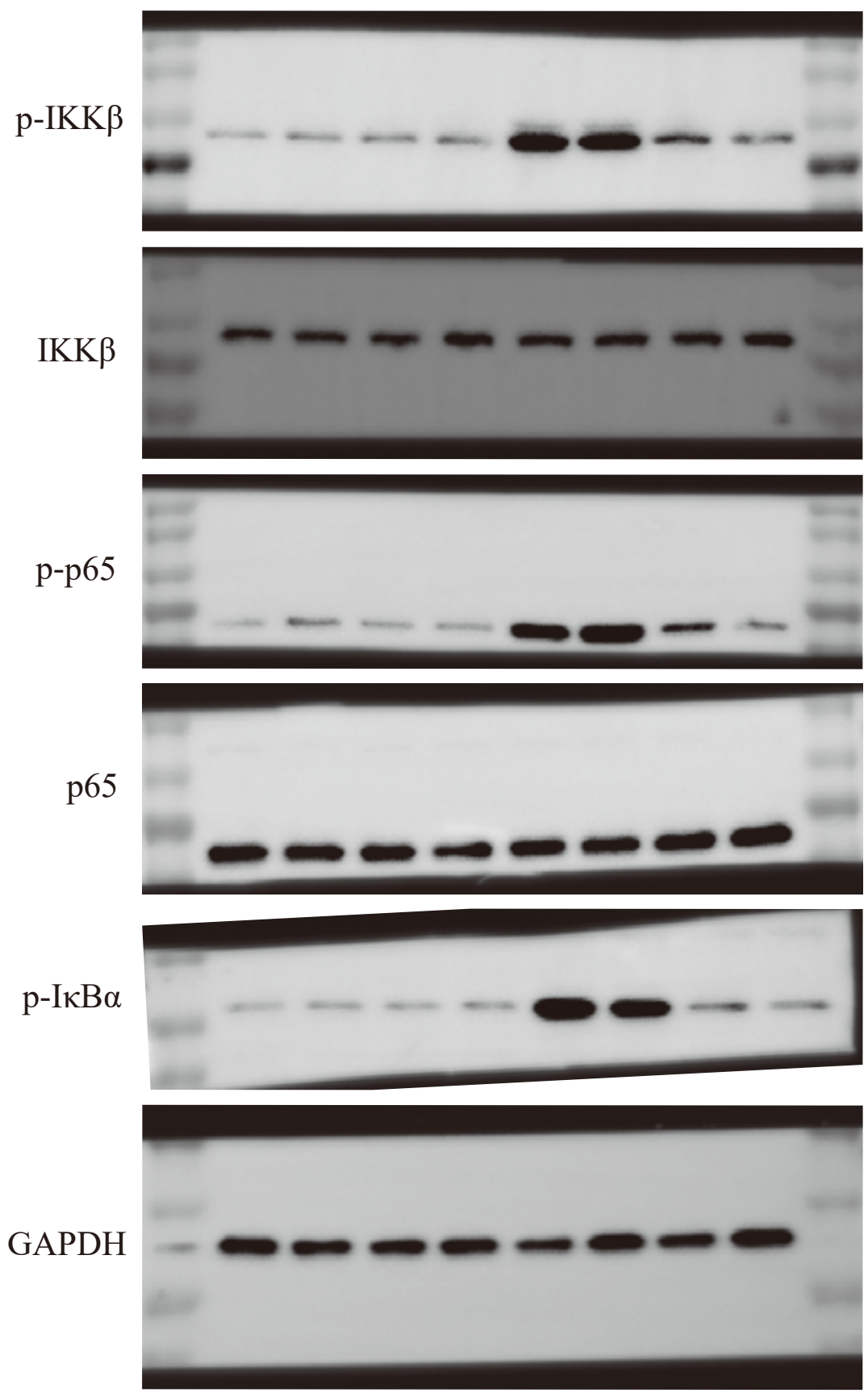

Fig 5E

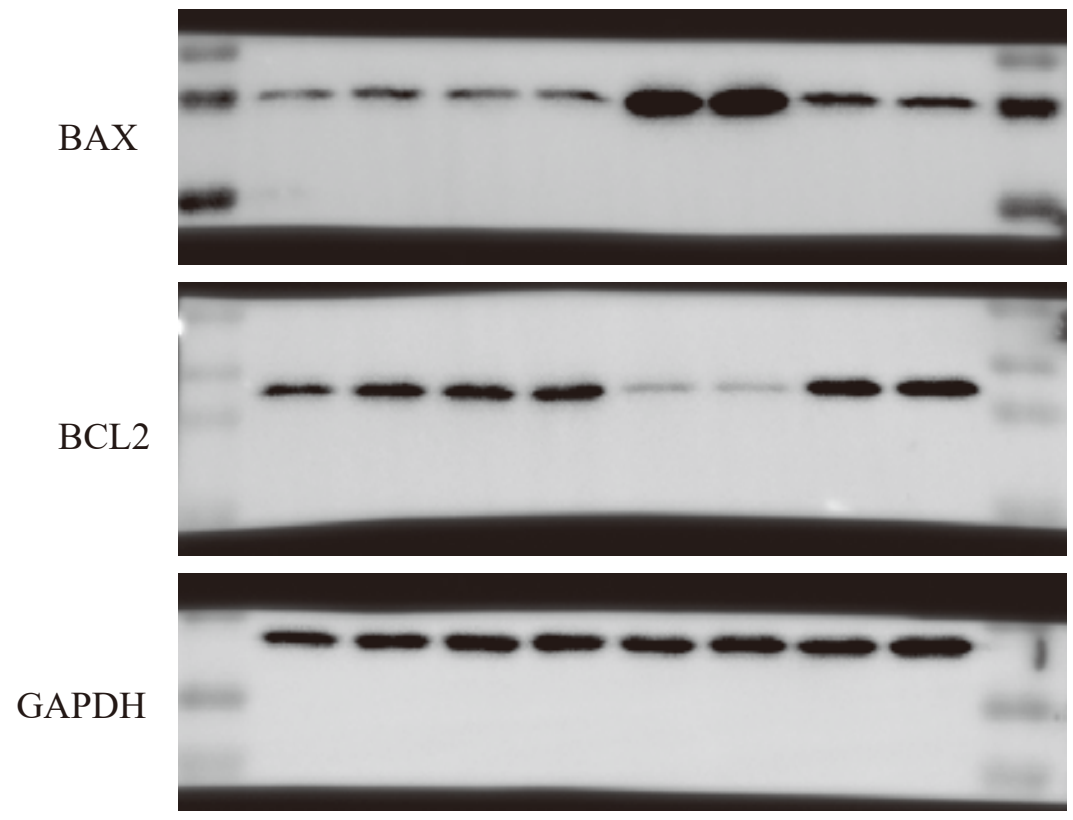

Fig 5K

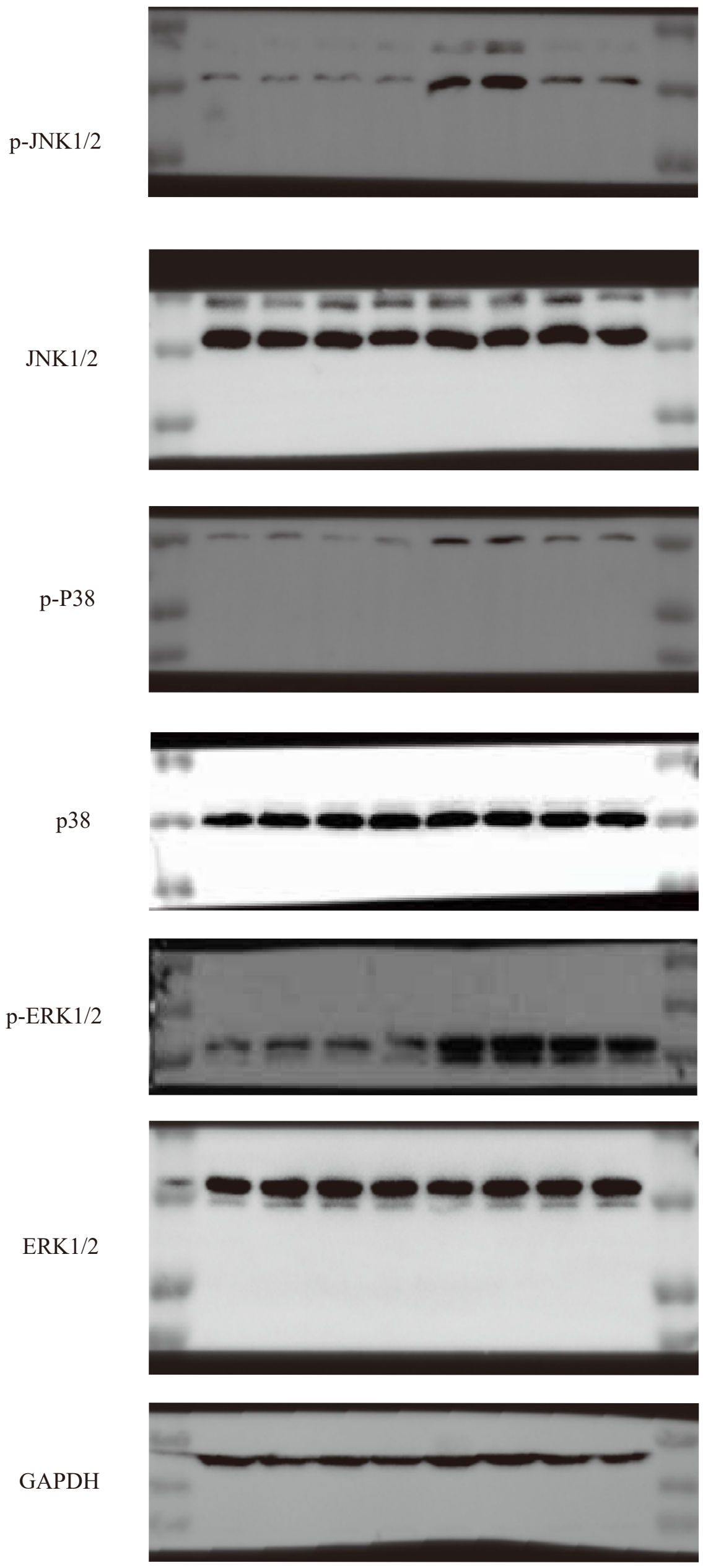

Fig 5J

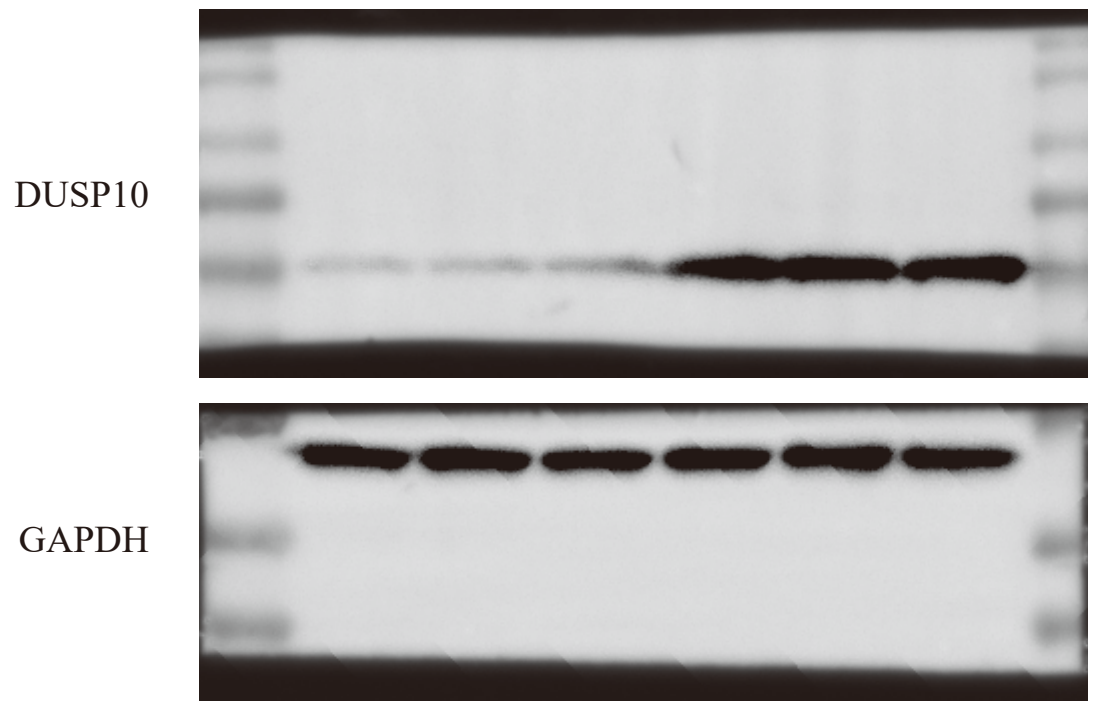

Fig 5L

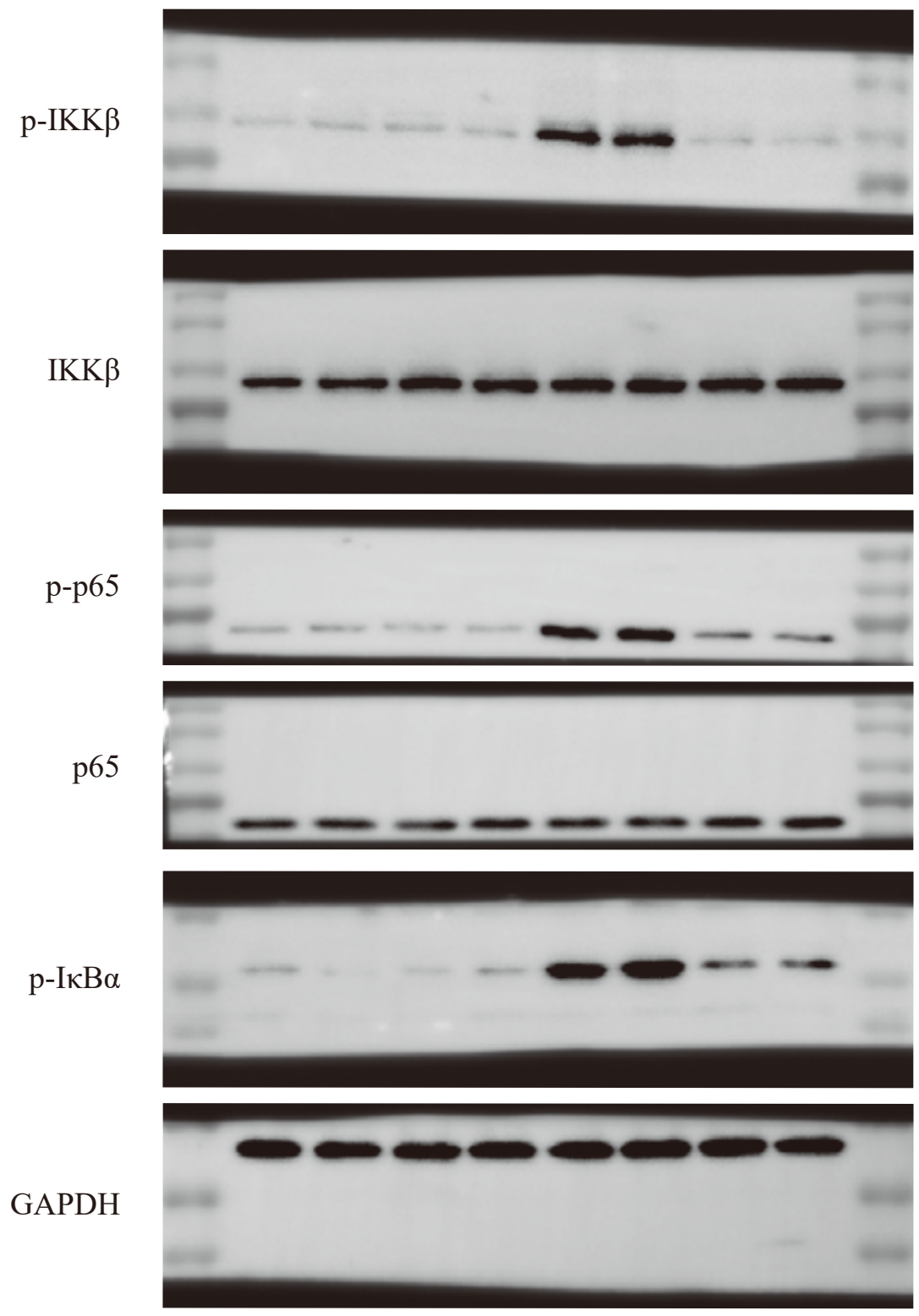

Fig 5M

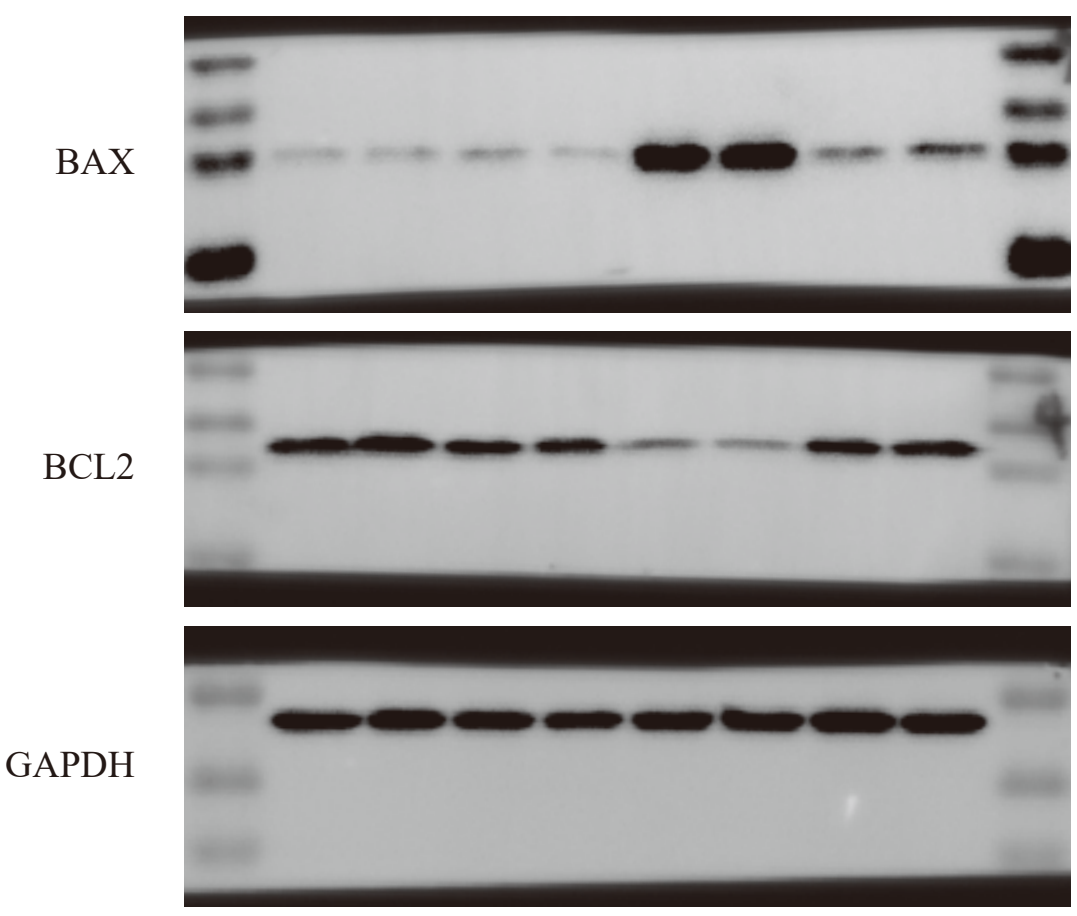

Fig 6A

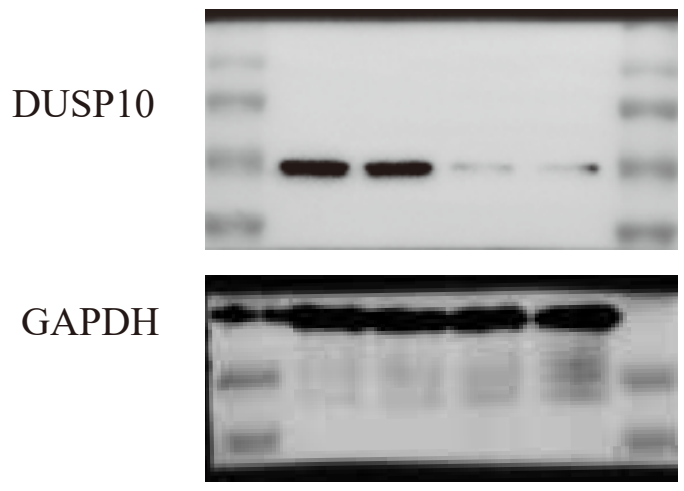

Fig 6B

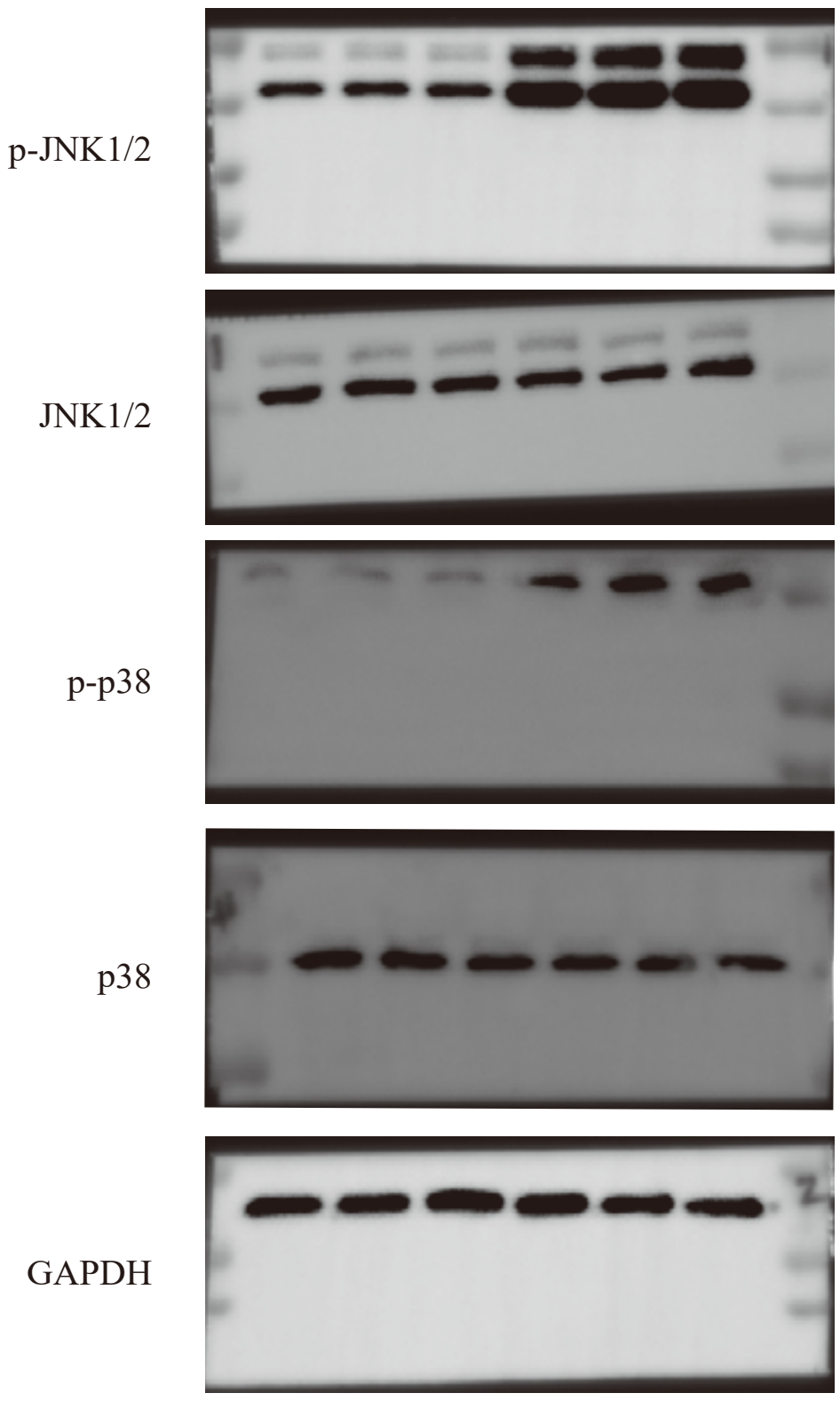

Fig 6C

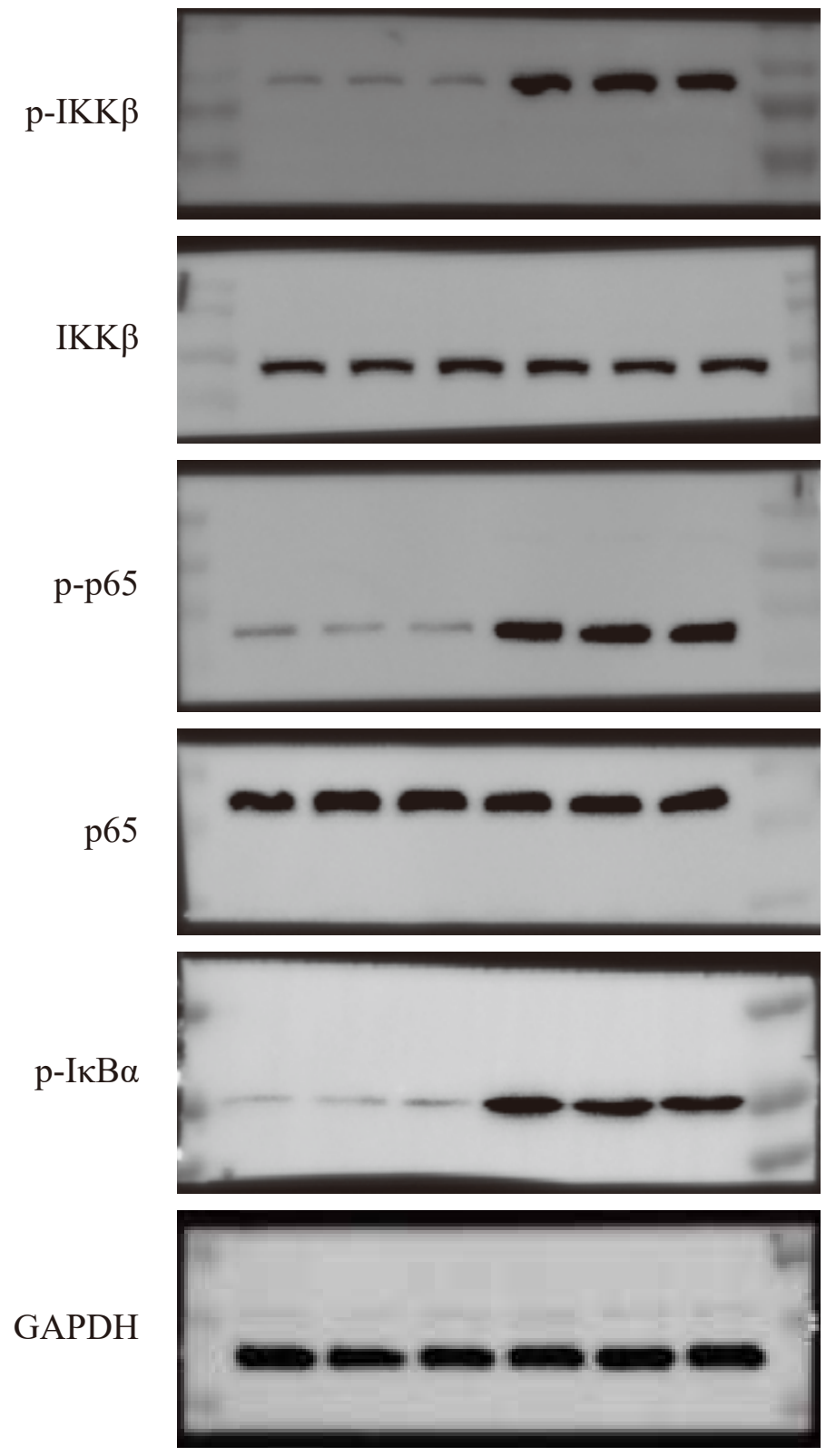

Fig 6D

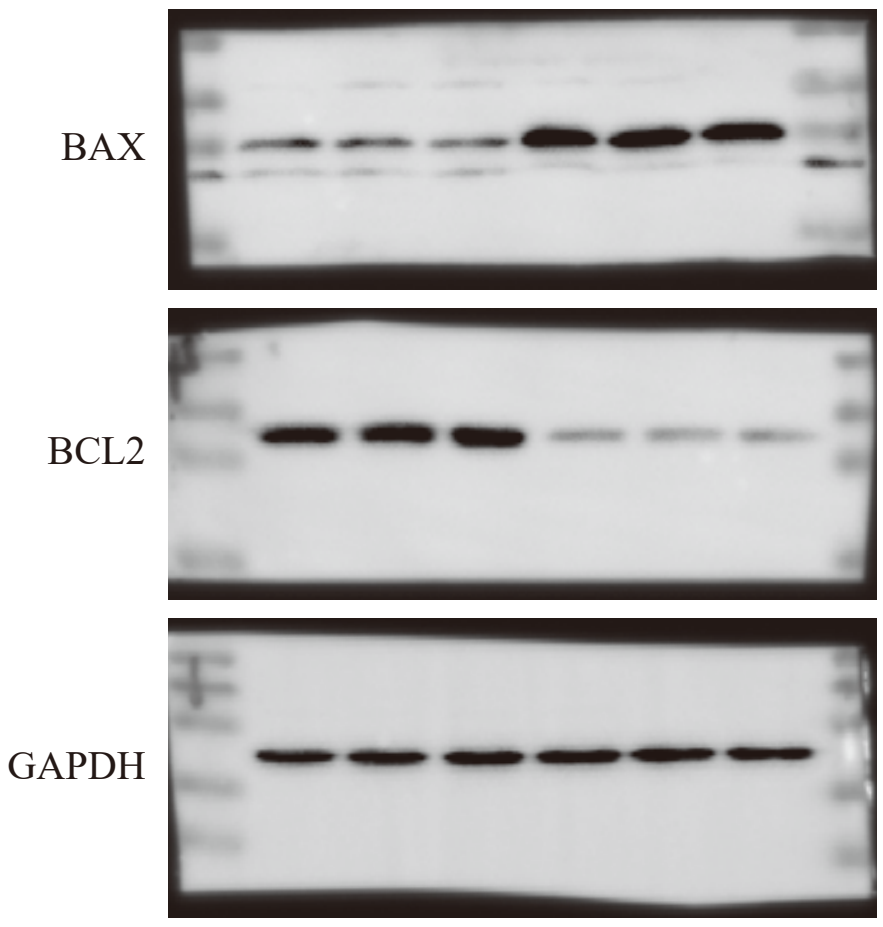

Fig 6I

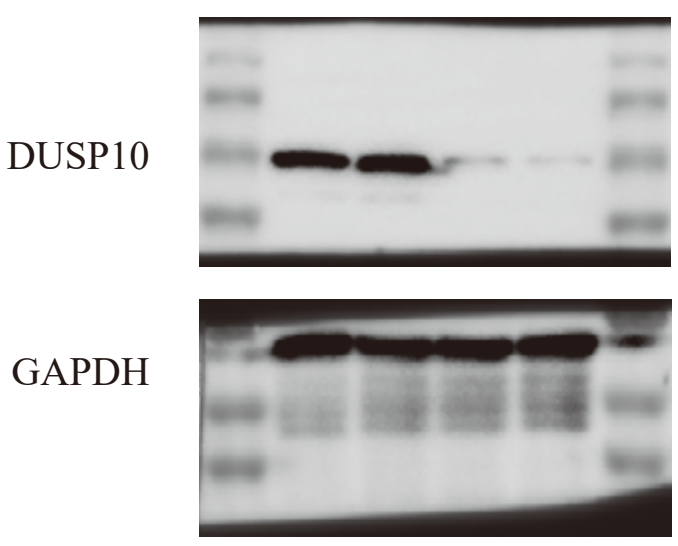

Fig 6J

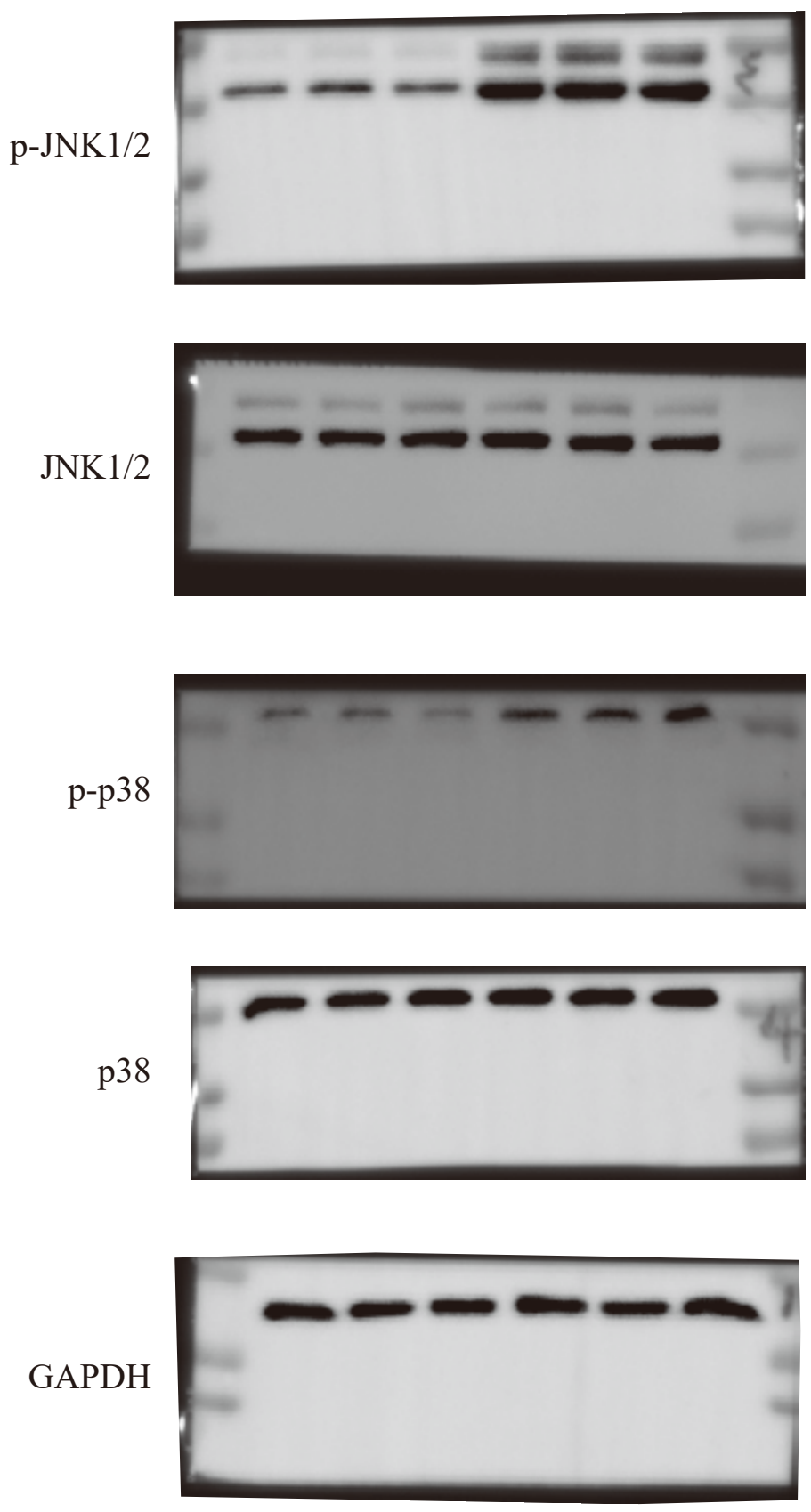

Fig 6K

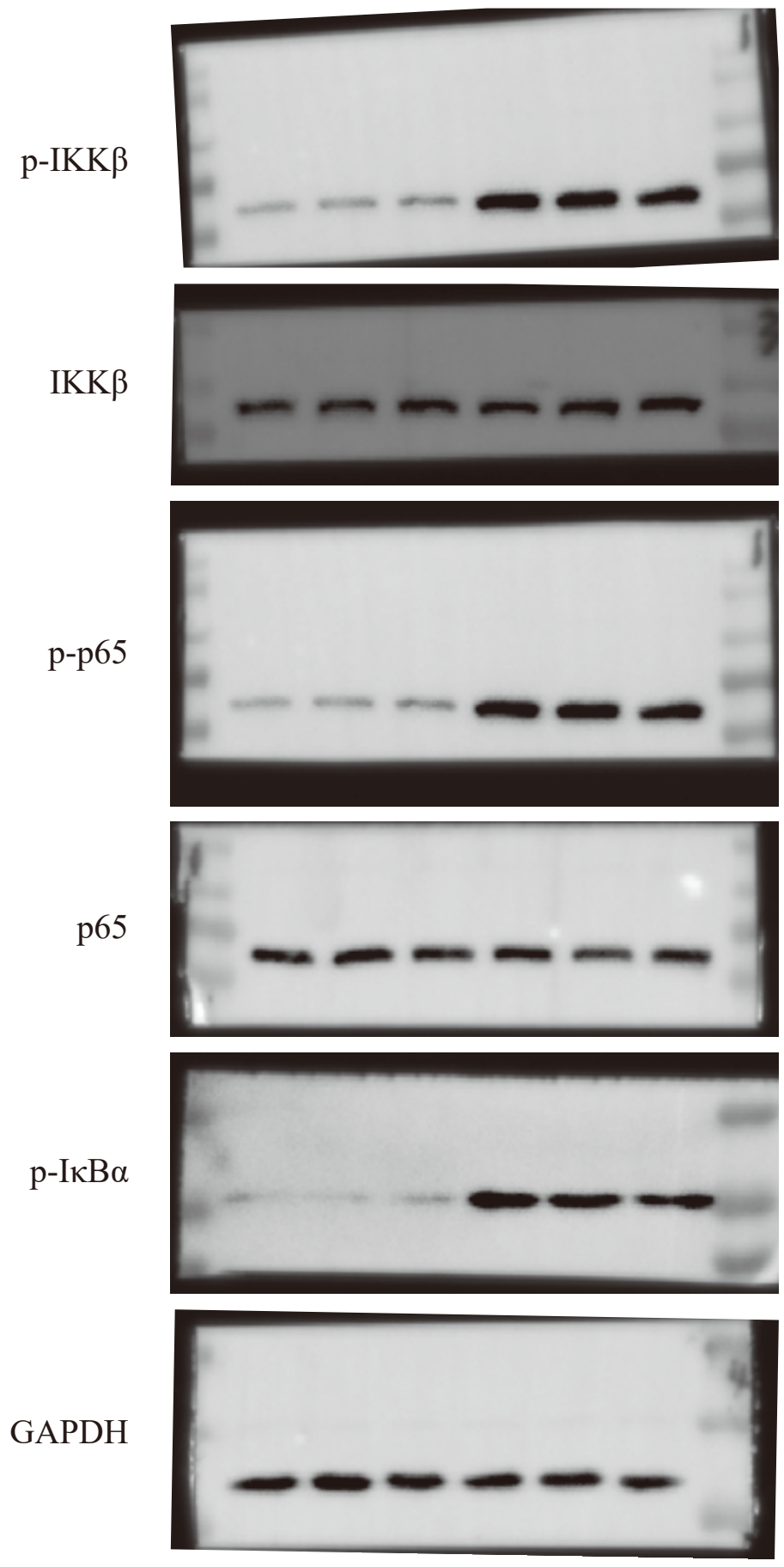

Fig 6L

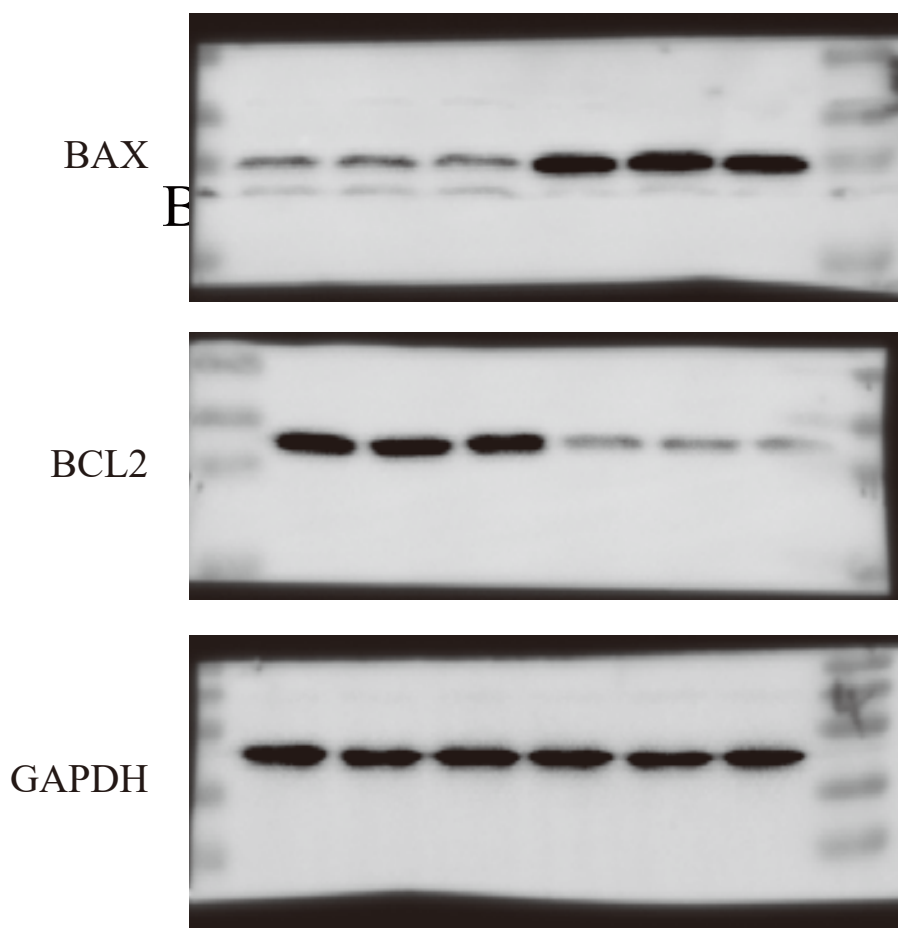

Fig 7A

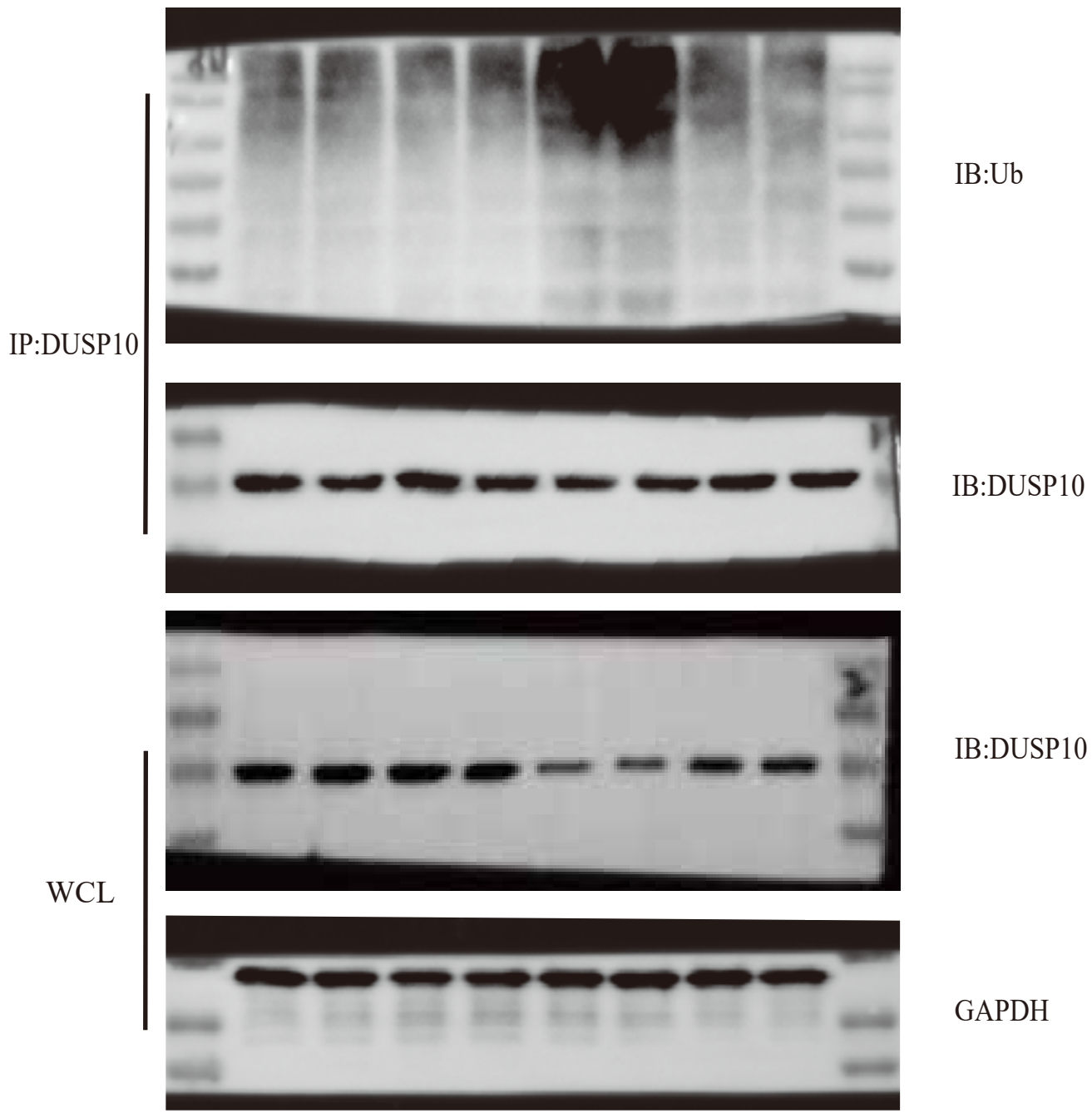

Fig 7B

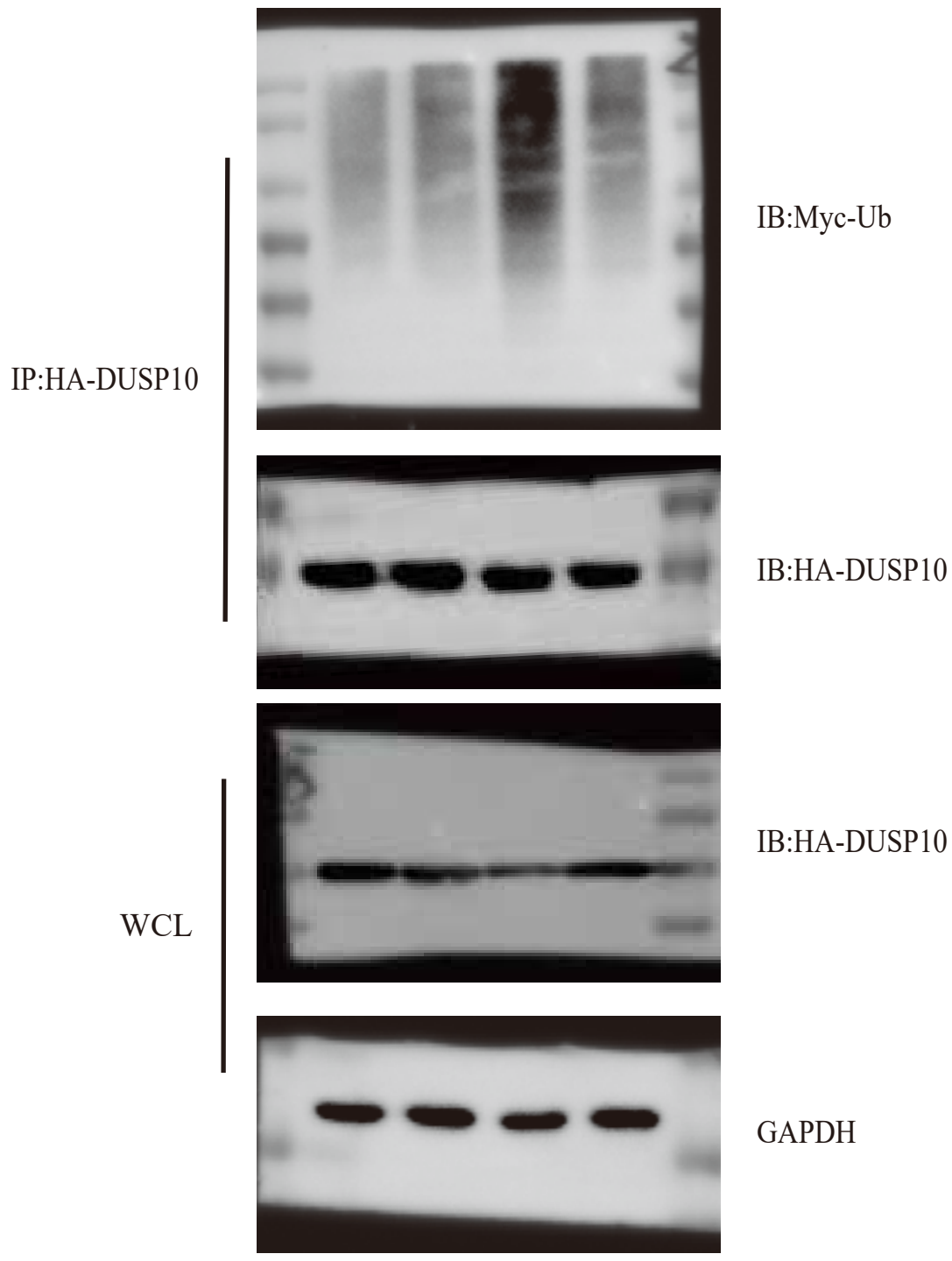

Fig 7C

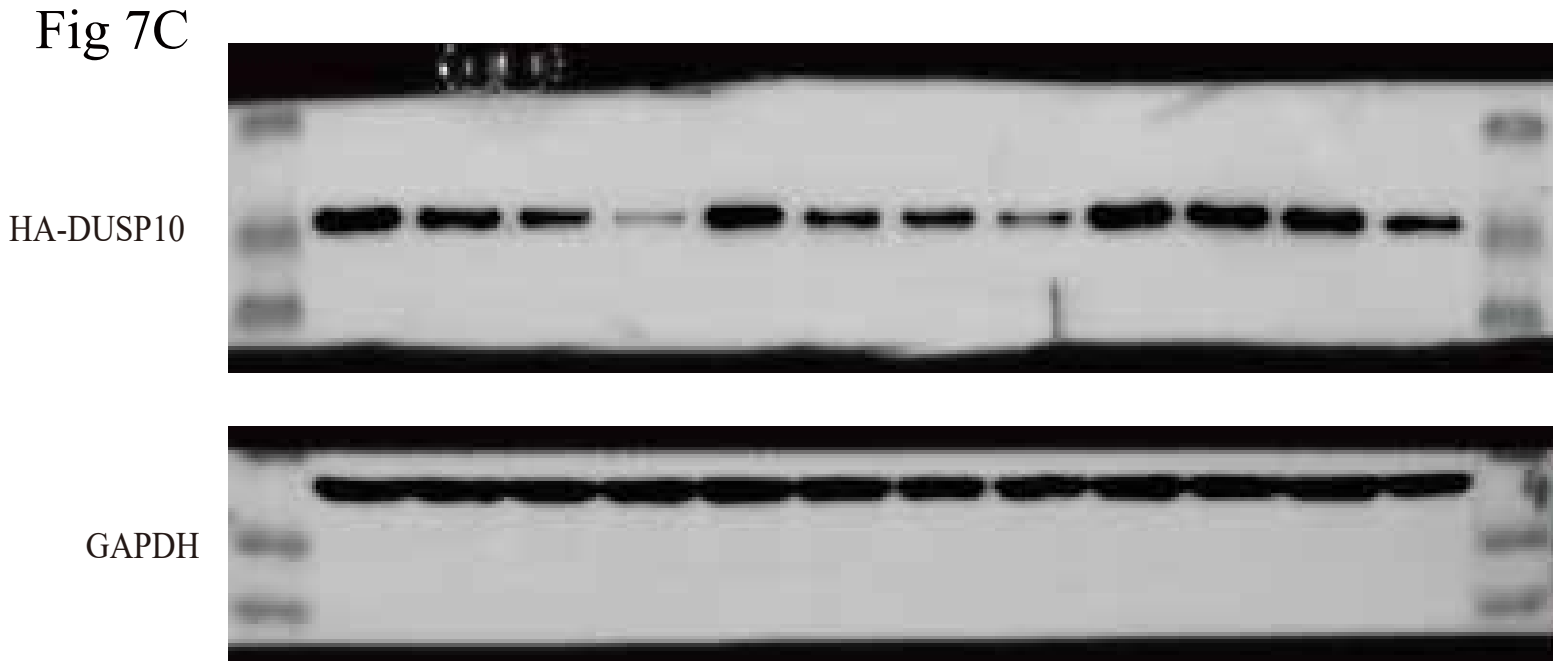

Fig 7D

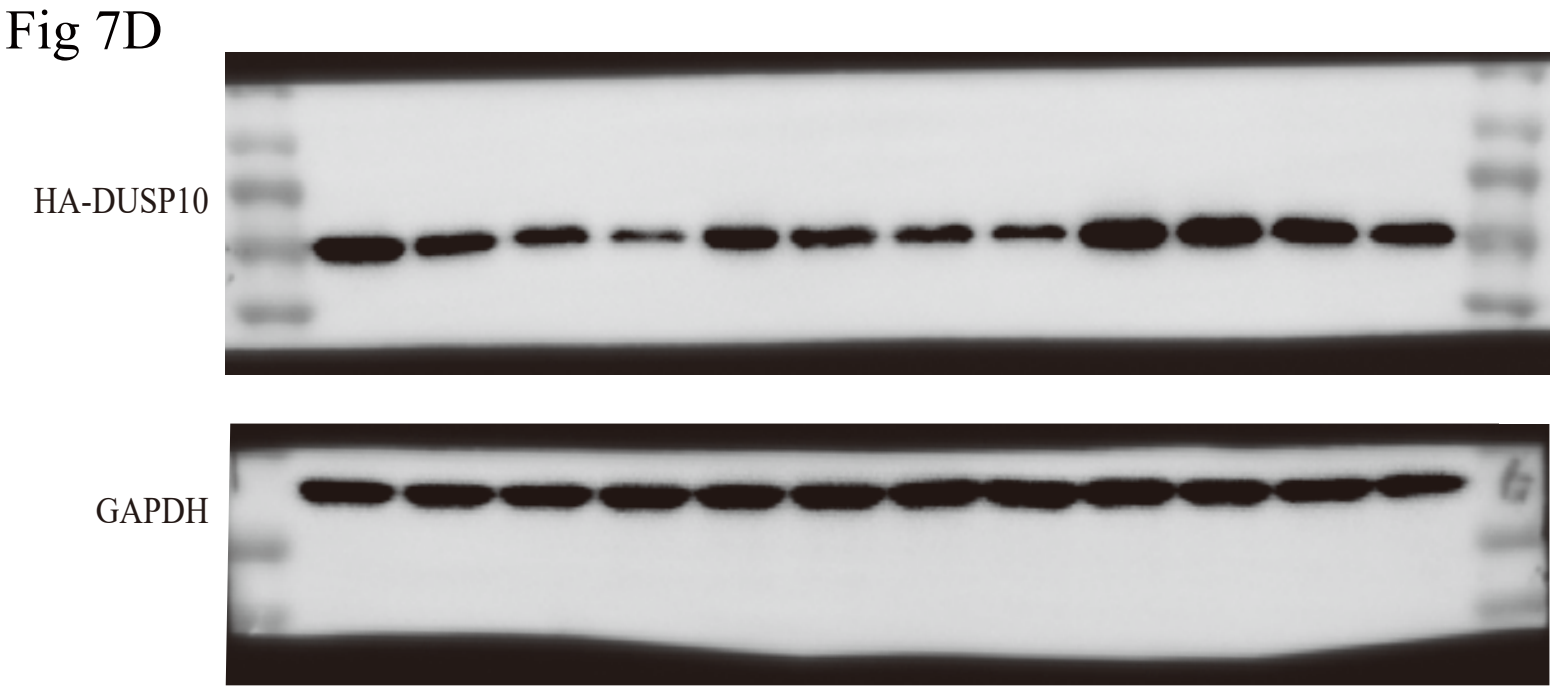

Fig 7E Left

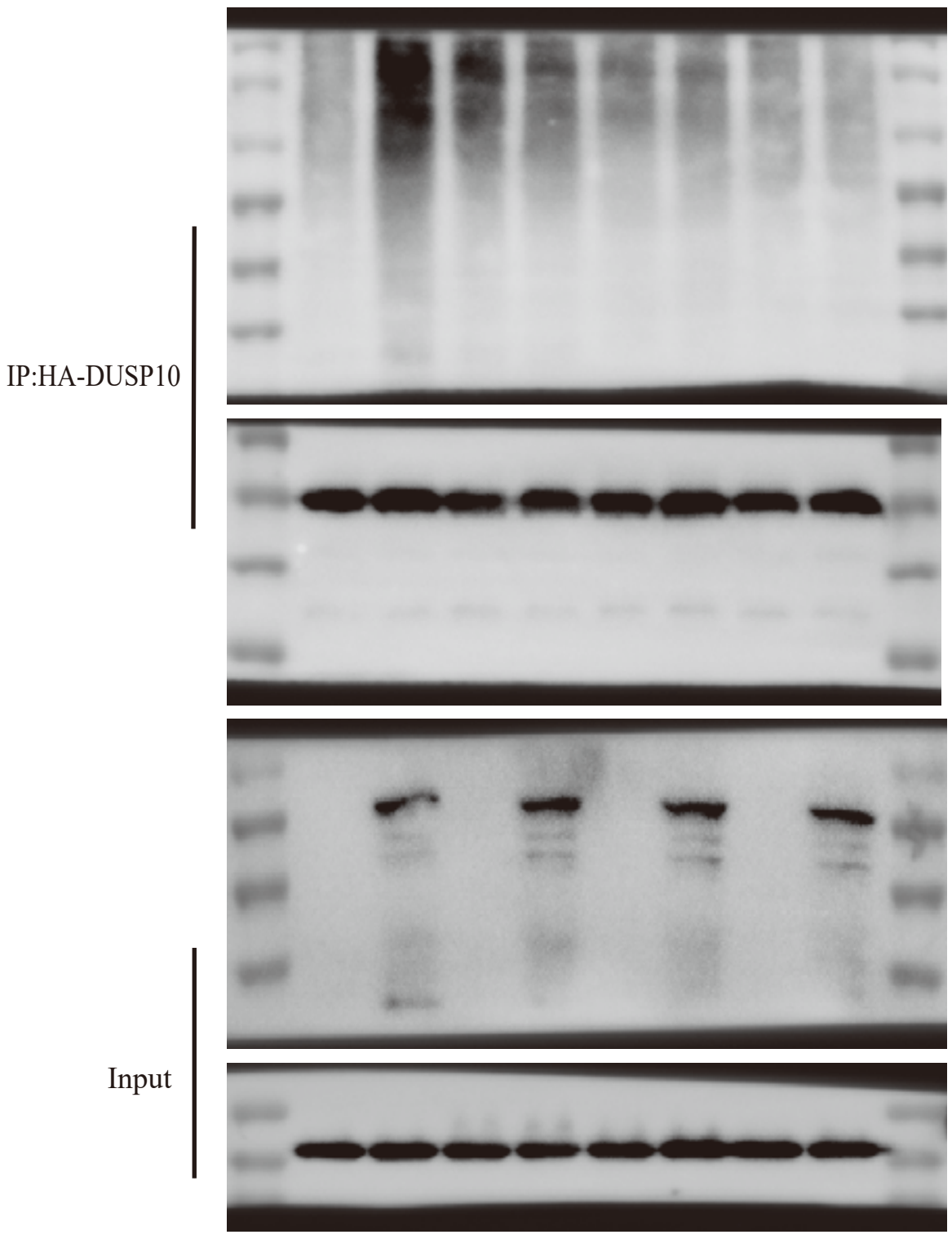

Fig 7E Right

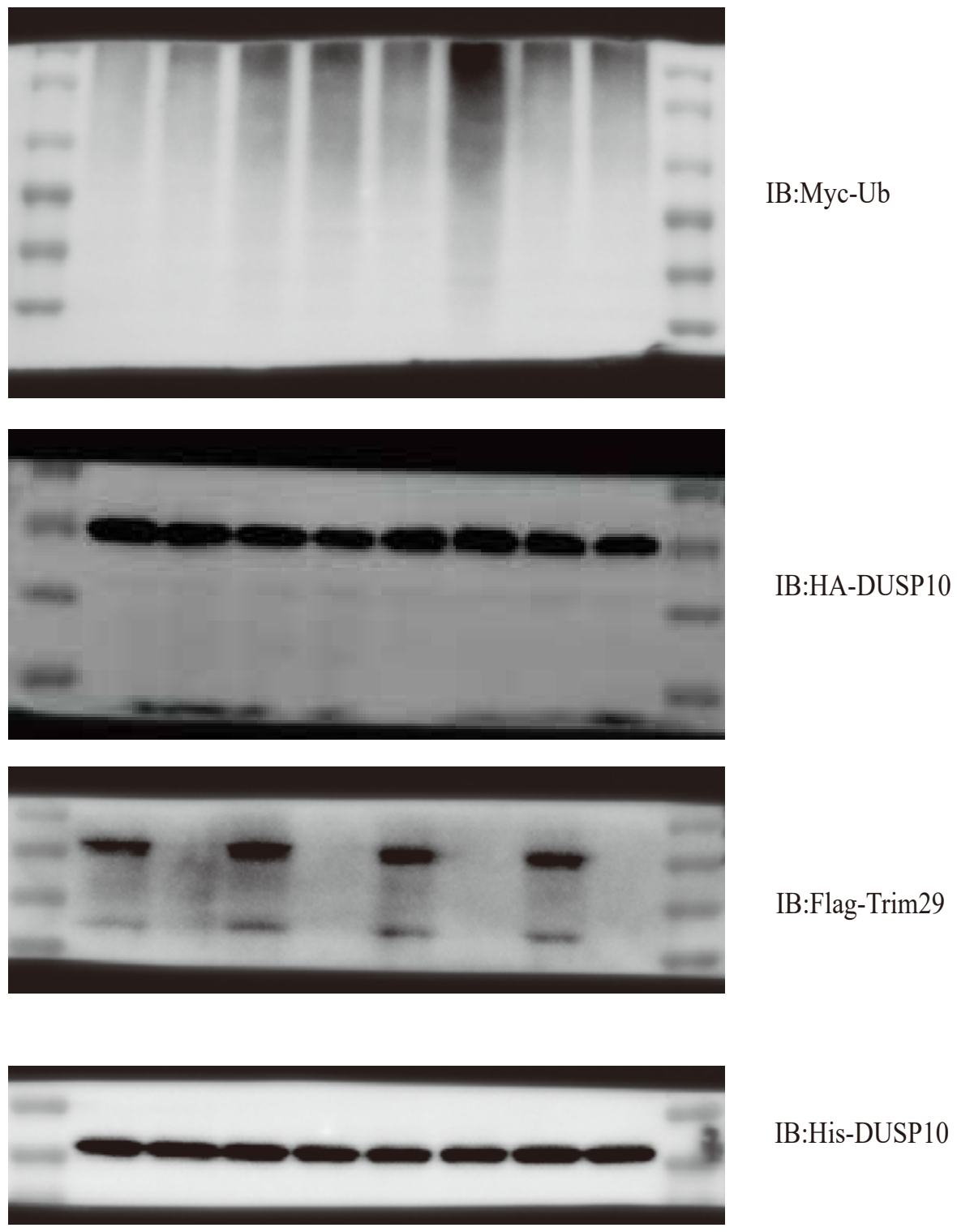

Fig 7F

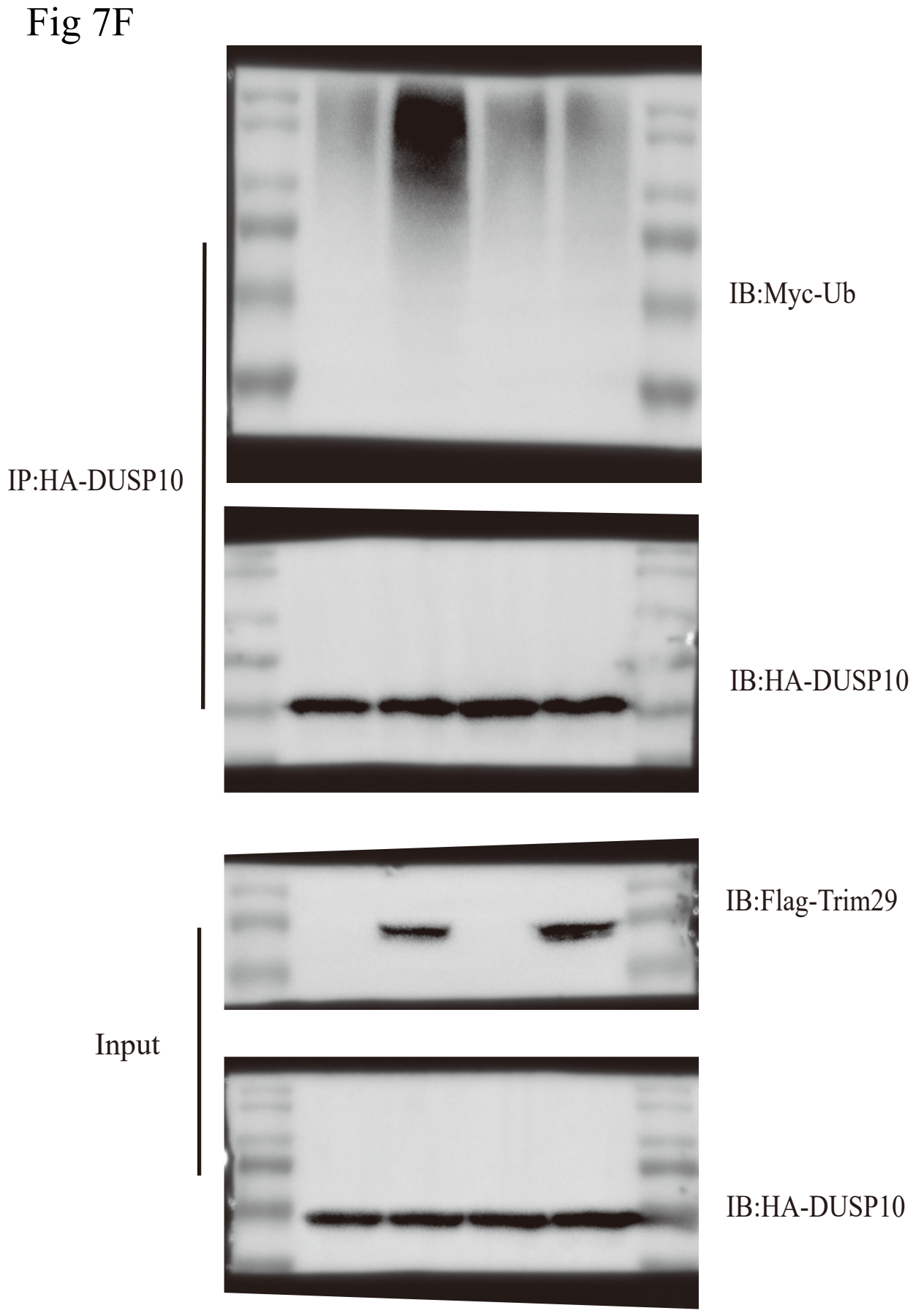

Fig 7H

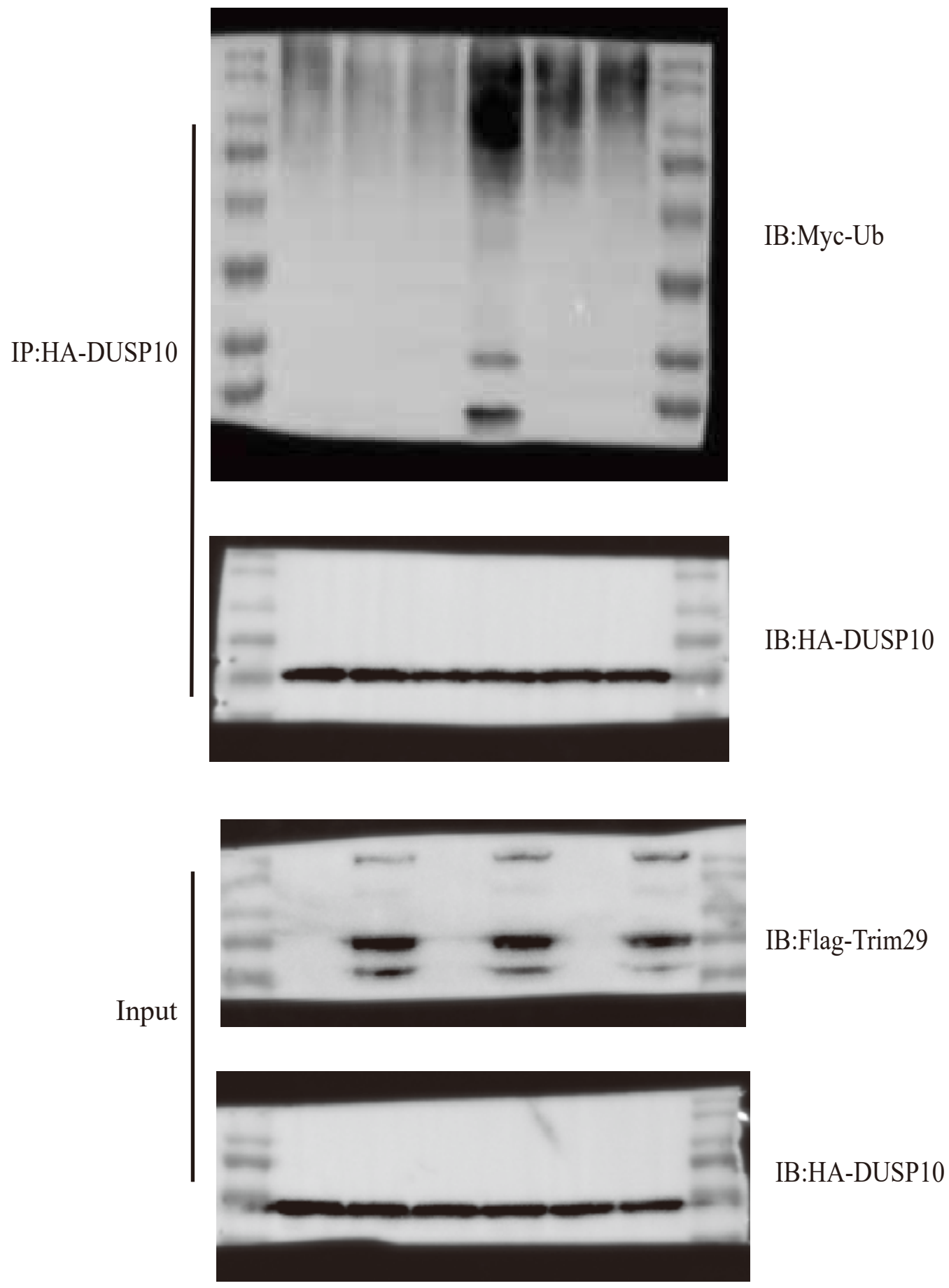

Fig 7G Left

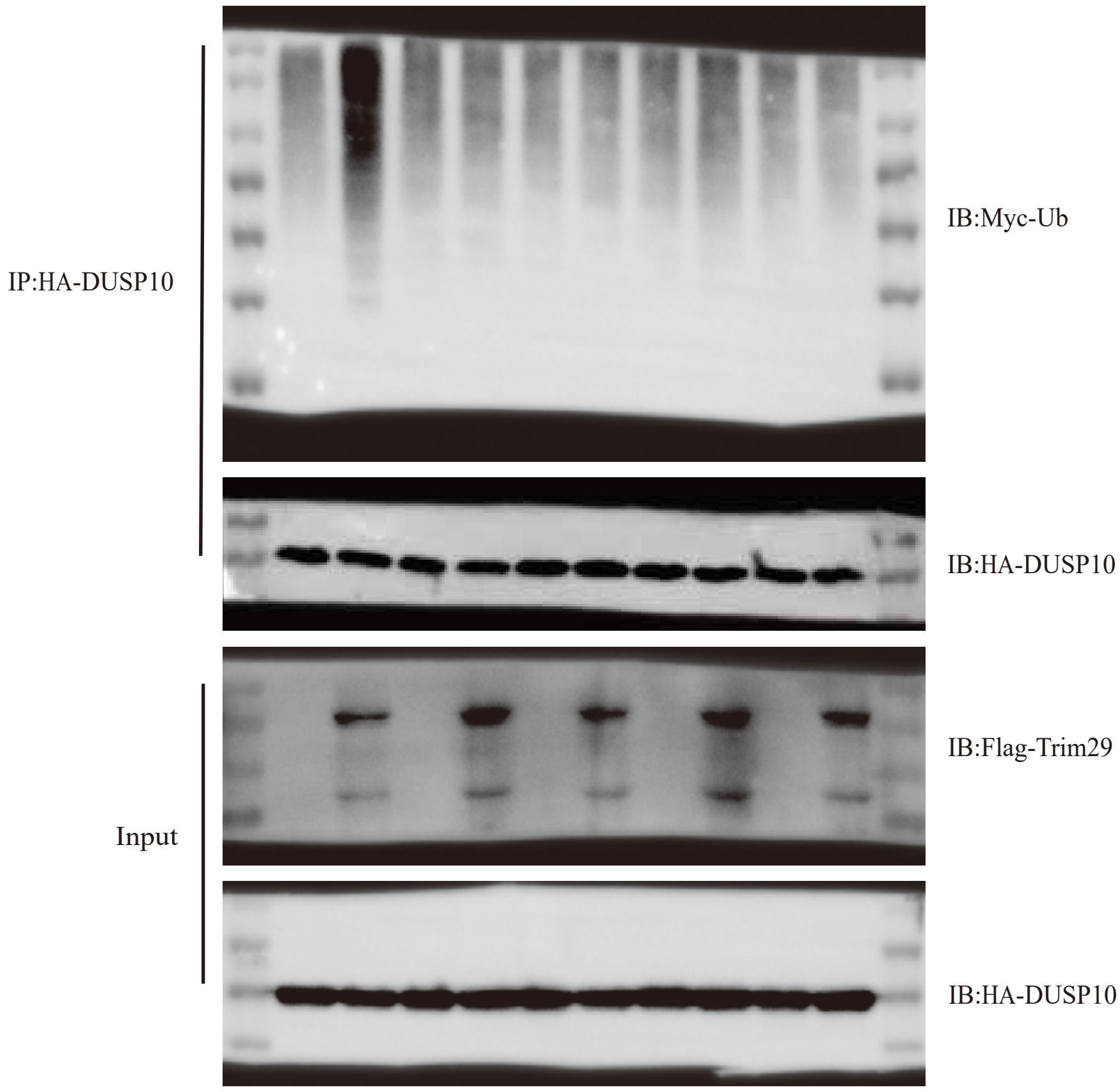

Fig 7G Right

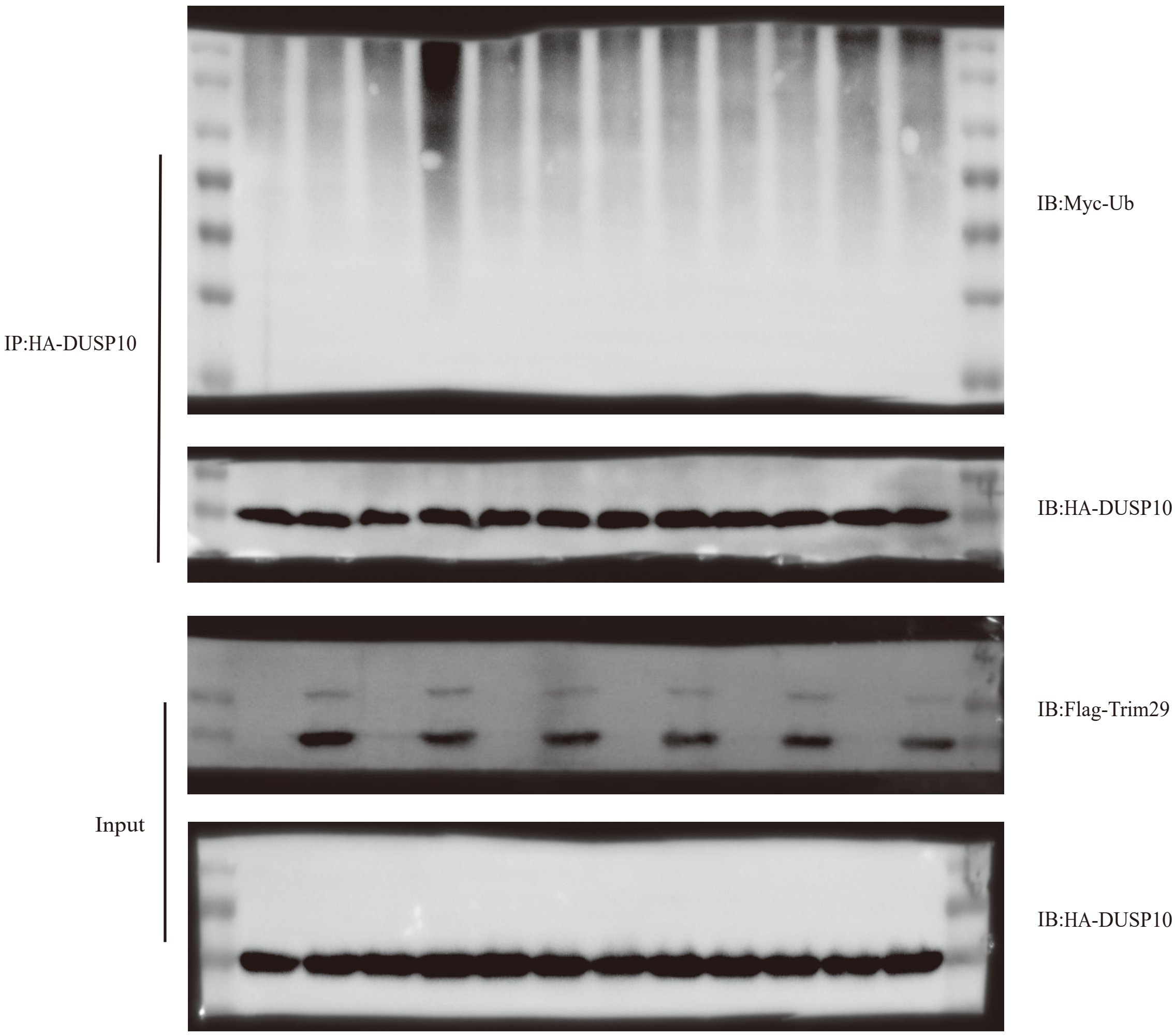

Figure SF

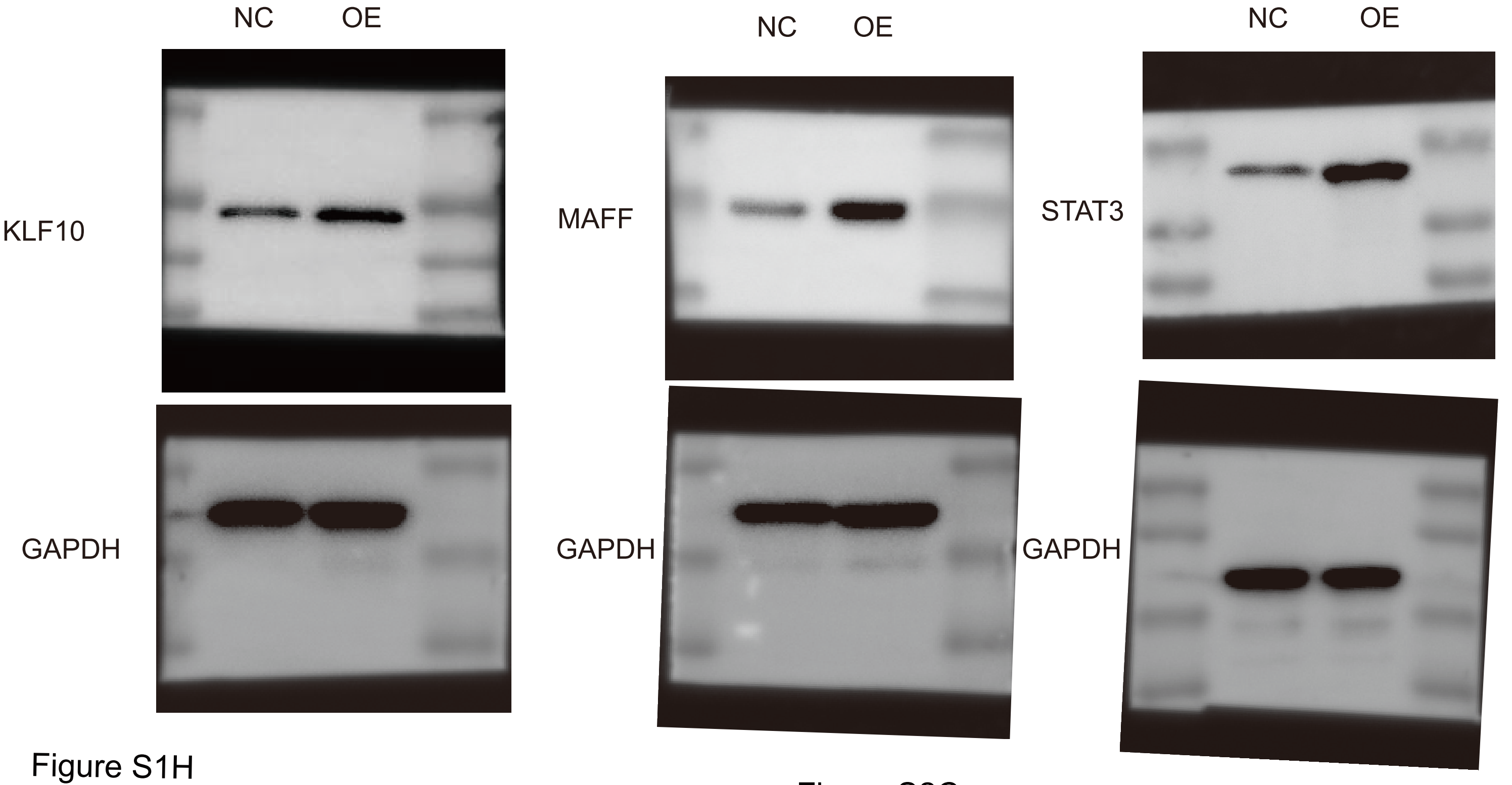

Figure S1H

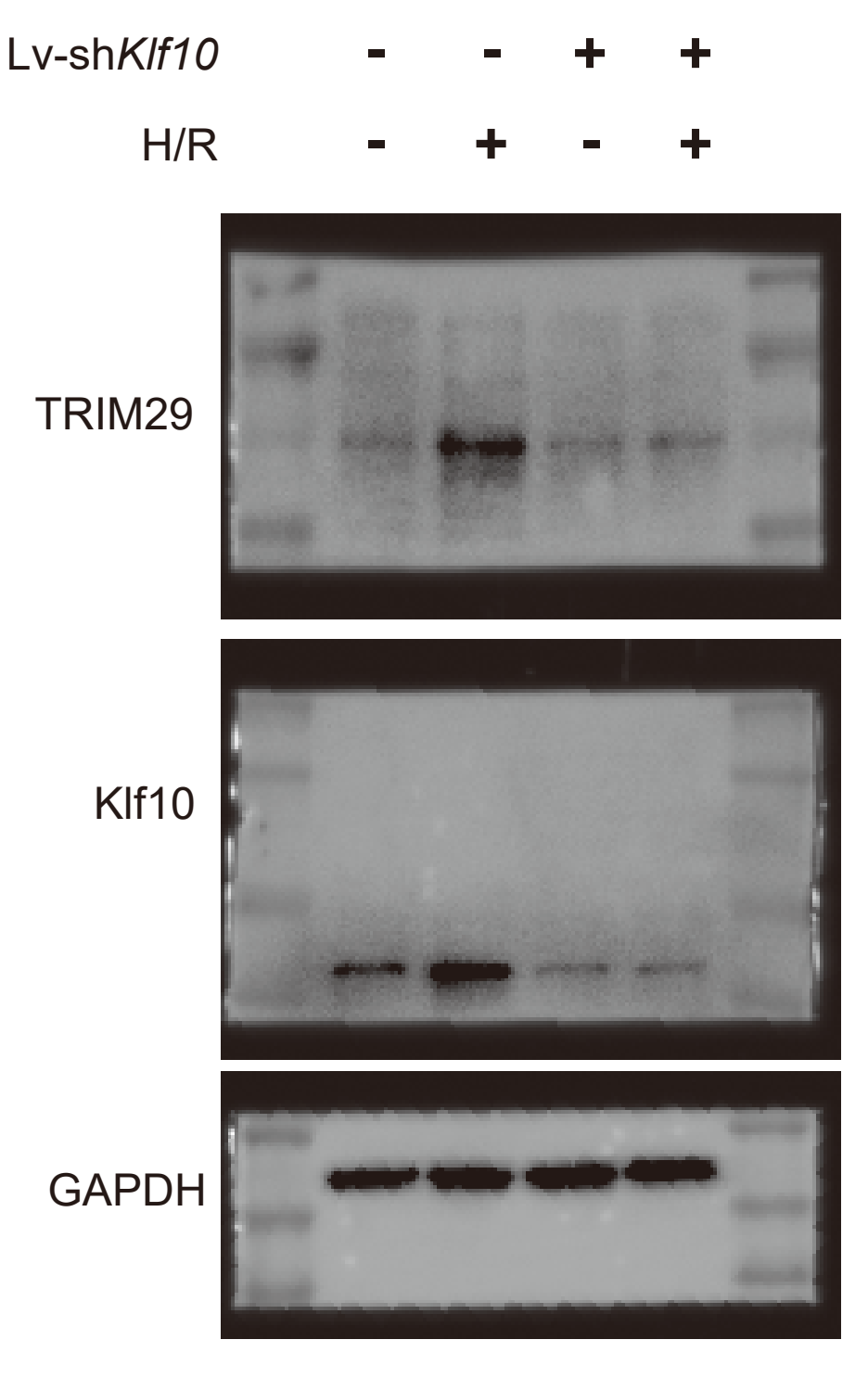

Figure S2C

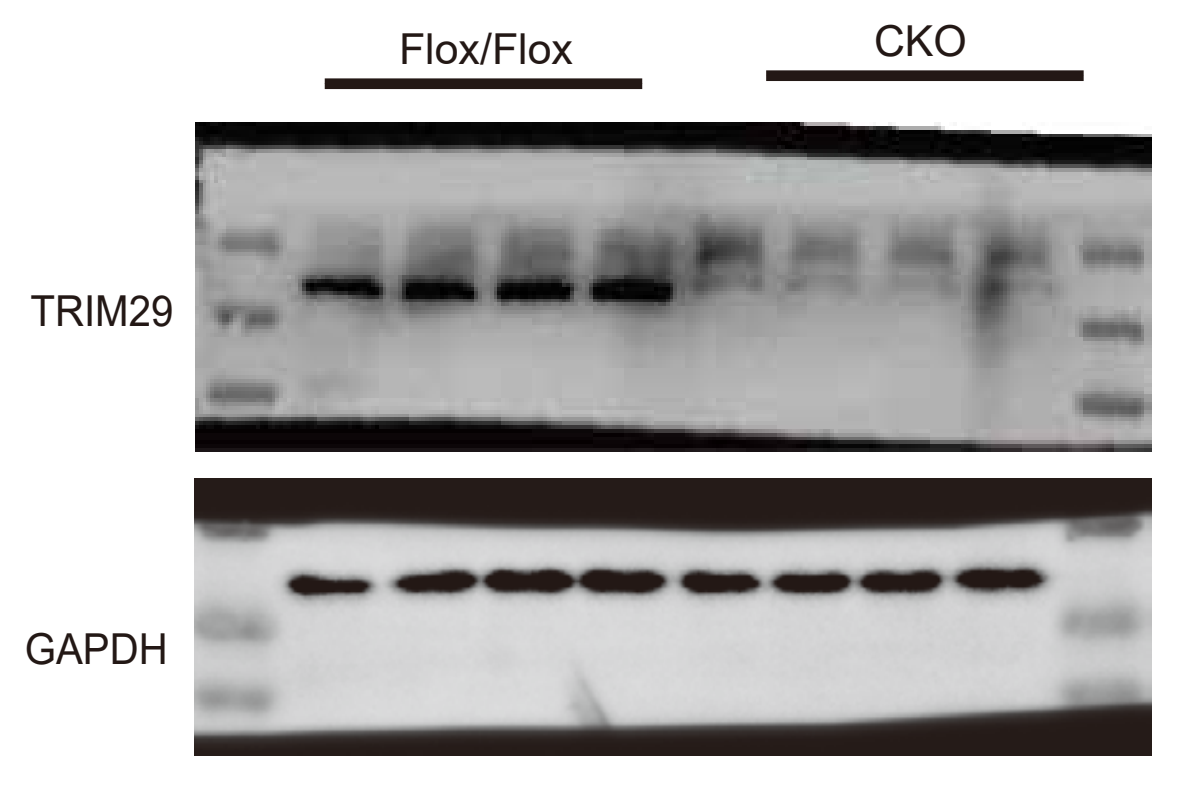

Figure S3G

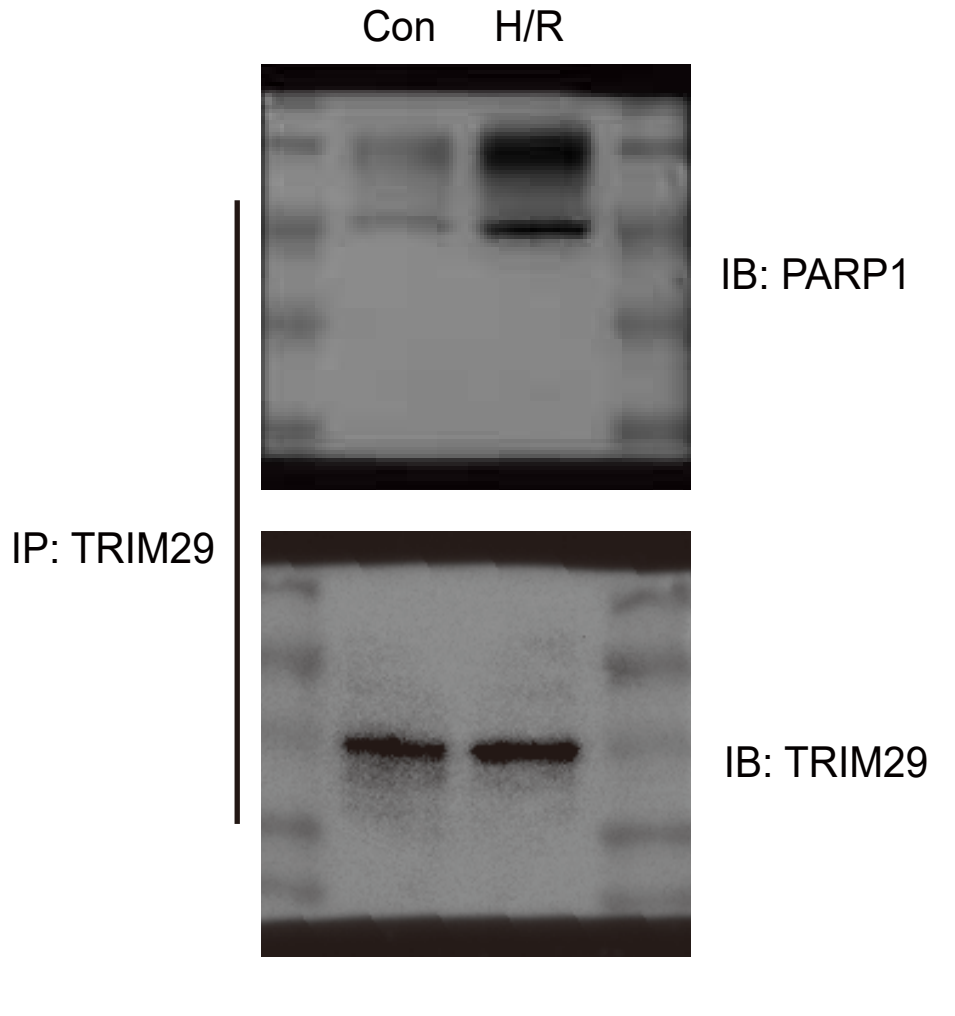

Figure S3H

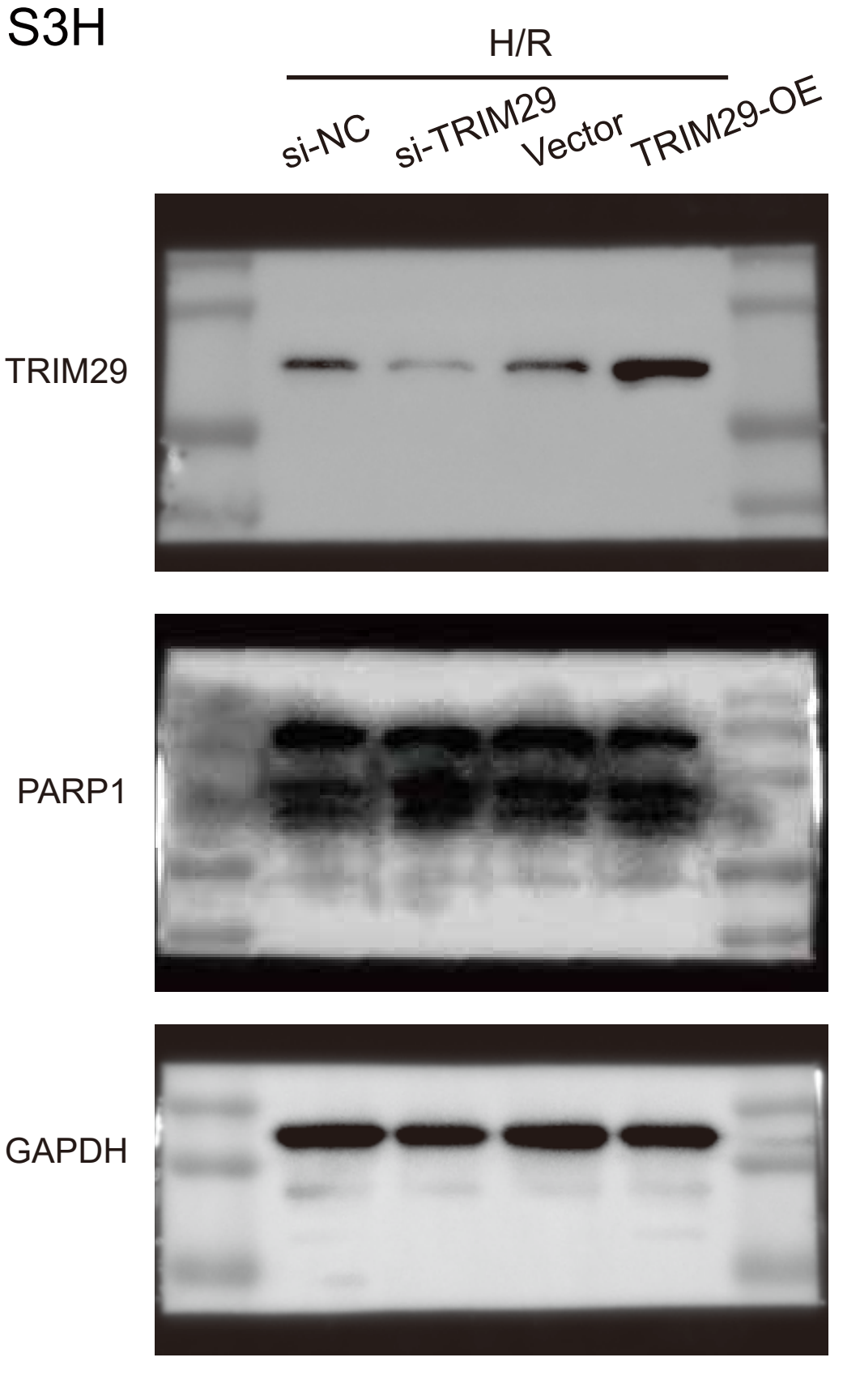

Figure S4J

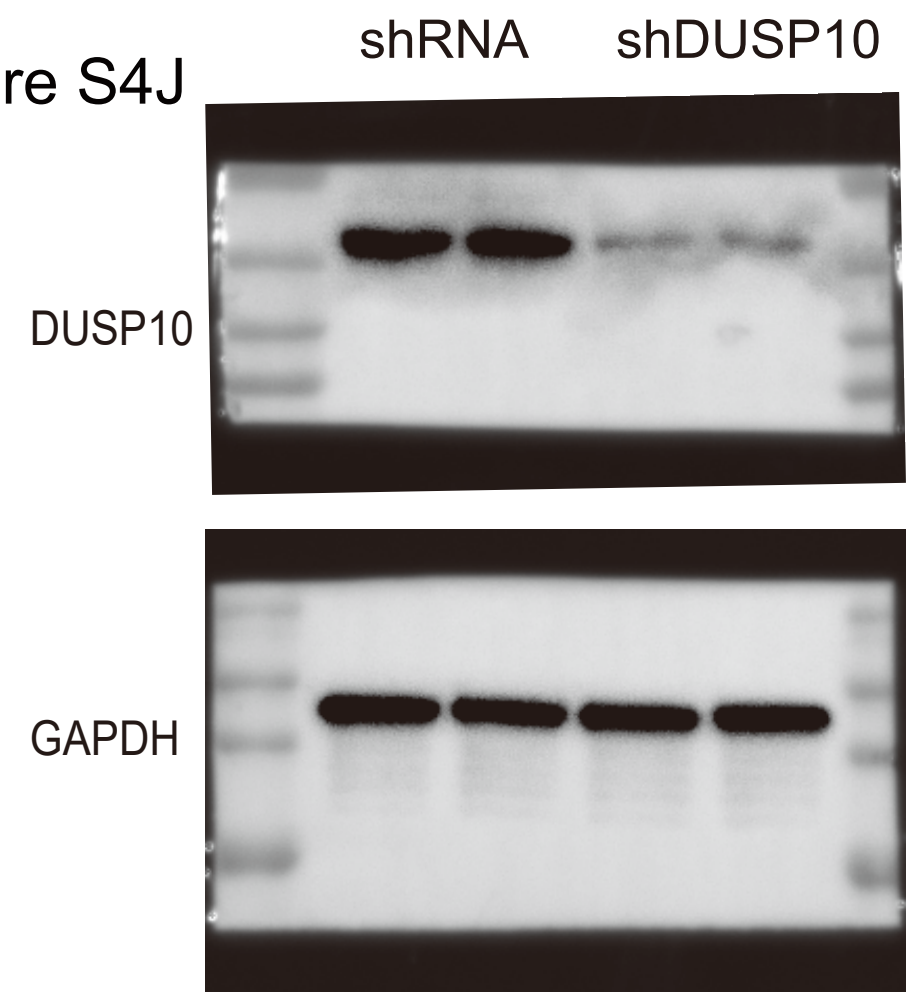

Figure S4L

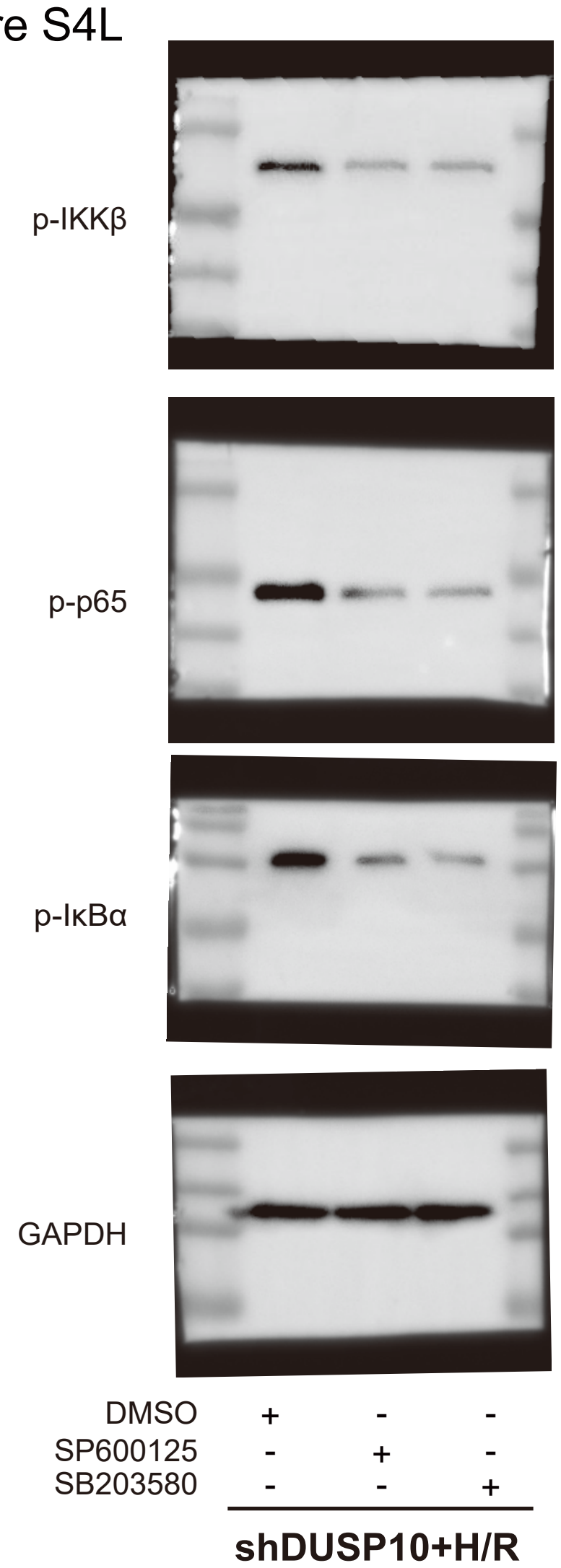

Figure S5B

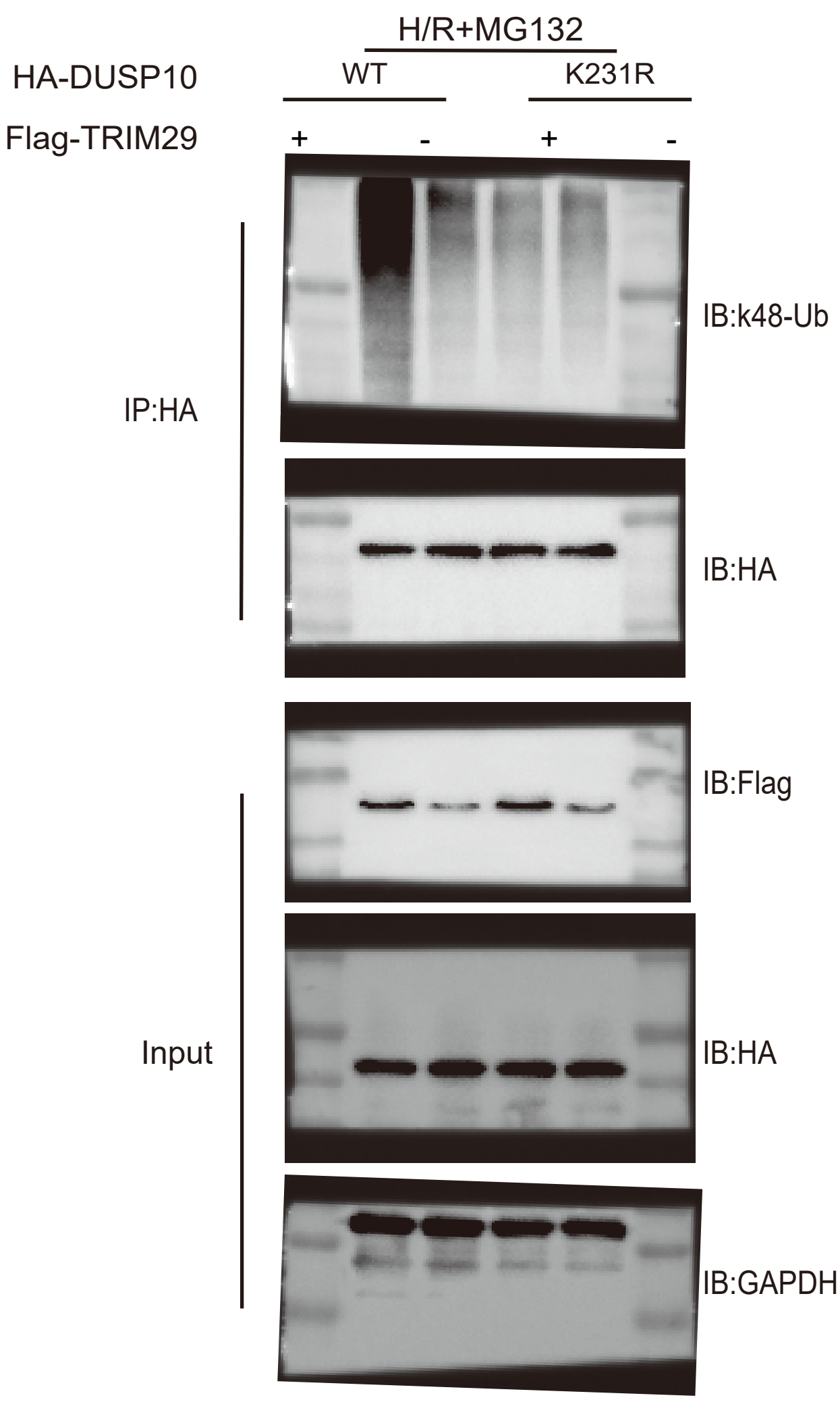

Figure S4N

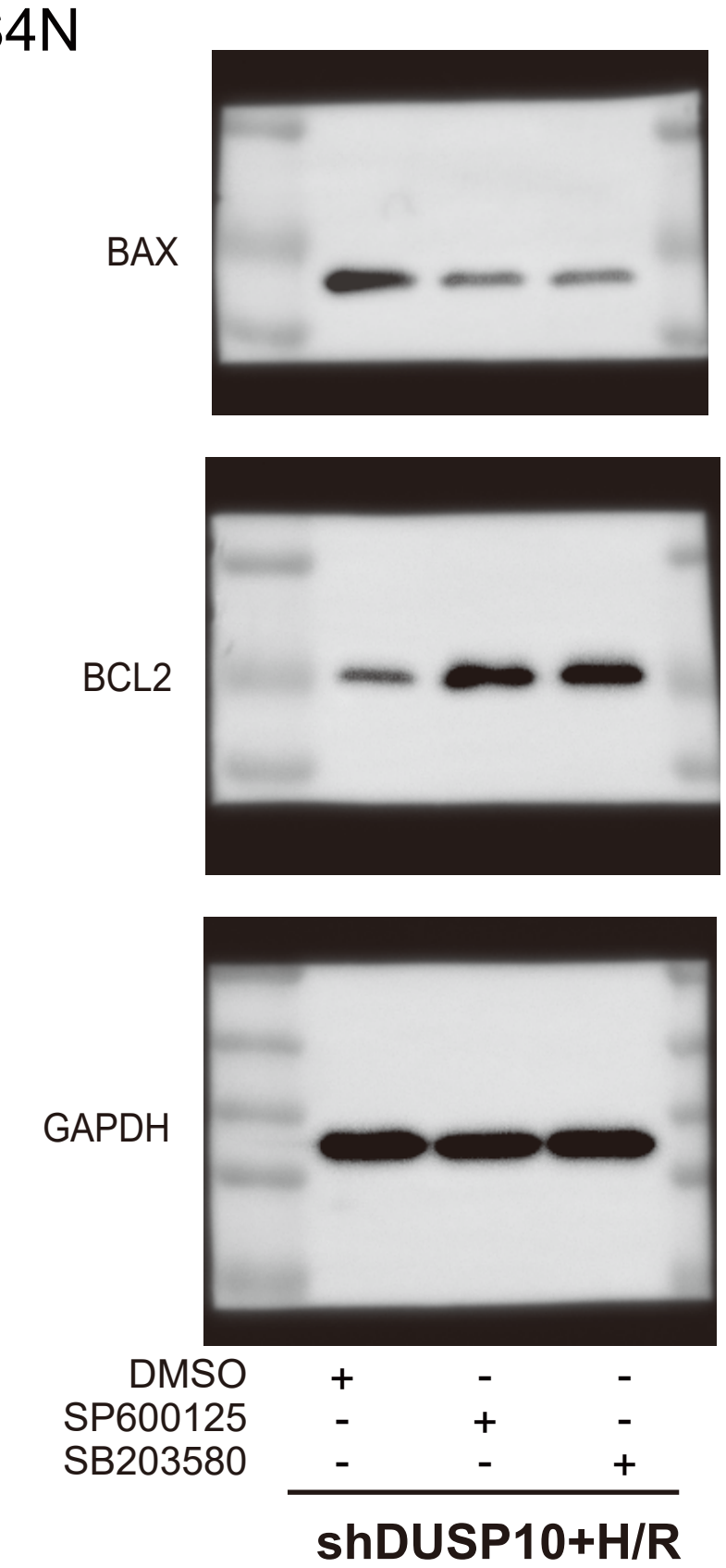

Supplement: Supplementary file 1 [file 41419_2026_8990_MOESM2_ESM.pdf]
