# Supplementary material for: The E3 ligase TRIM29 drives renal ischemia-reperfusion injury by targeting DUSP10 for proteasomal degradation
Source: Cell Death Dis. 2026 Jun 13;17(1):742. doi: 10.1038/s41419-026-08990-w (PMC13487226; doi:10.1038/s41419-026-08990-w)
Supplement: Supplementary file 2 [file 41419_2026_8990_MOESM1_ESM.docx]

**Supplementary data for:**

**The E3 ligase TRIM29 Drives Renal Ischemia-Reperfusion Injury by Targeting DUSP10 for Proteasomal Degradation**

**Table of contents**

Supplementary Materials and Methods………………………………………….……1

Supplementary Figures……………………………………...…………………….….11

Supplementary Tables……………………………………………...……....………...20

**Materials and Methods**

**Bioinformatic Analysis**

**Data Source and Preprocessing**

Transcriptomic datasets were retrieved from the GEO database (GSE192883), comprising sequencing data from Sham and RIRI murine models (n = 3 per group). Additionally, independent RNA sequencing was performed in-house. For internal samples, total RNA was isolated from kidney tissues using TRIzol reagent (Thermo Fisher Scientific, MA, USA). Sequencing libraries were constructed and sequenced on the BGISEQ-500 platform (50 bp single-end reads). Clean reads were mapped to the mouse reference genome (mm10) using HISAT2 (v2.1.0), and gene expression was quantified as FPKM using StringTie (v1.3.3b). All datasets underwent quality control, adapter trimming, and normalization prior to analysis.

**Differential Expression and Enrichment Analysis**

Differential expression analysis for microarray data was performed using the limma R package (v3.62.1) (|log2FC| > 0.7, *p* < 0.05). In-house RNA-seq data were analyzed using DESeq2 (v1.2.10) (|log2FC| > 0.7, FDR < 0.05). Differentially expressed genes (DEGs) were visualized using volcano plots and heatmaps. KEGG pathway enrichment was analyzed via clusterProfiler (v4.14.4) (*p* < 0.05). To specifically interrogate ubiquitination processes, ubiquitination-related gene sets from the Gene Ontology (GO) database were intersected with DEGs.

**Protein-Protein Interaction (PPI) Network Analysis**

DEGs were mapped to the STRING database (v11.5) with a minimum interaction confidence score of 0.7. The resulting network was visualized in Cytoscape (v3.9.1), and hub genes were identified based on node degree.

**Adeno-associated Virus 9 (AAV9) Injection**

To evaluate DUSP10 function, mice received tail vein injections of AAV9 encoding DUSP10 (AAV9-OE-DUSP10) or empty vector (AAV9-EV). To assess DUSP10 knockdown in TRIM29 CKO mice, animals were injected with AAV9-shDUSP10 or a non-targeting control (AAV9-shNC). All AAV9 vectors were constructed and packaged by GenePharma (Shanghai, China). Viral vectors (10¹¹ vg/mL, 100 µL/mouse) were administered two weeks prior to RIRI surgery to ensure optimal transgene expression.

**In Vitro Gene Modulation**

**Plasmid Transfection**

To investigate protein interactions and ubiquitination mechanisms, mammalian expression vectors encoding Flag-tagged TRIM29, HA-tagged DUSP10, and Myc-tagged Ubiquitin were utilized. TRIM29 deletion mutants (Full length, 1–258, 1–356, 259–588, 357–588, ΔBB, and ΔCC) and DUSP10 truncation mutants (Full length, 1–167, 168–321, and 322–482) were generated via PCR cloning, with specific primer sequences listed in Supplementary Table S1. The ubiquitin linkage-specific mutants retaining only a single lysine residue (K6O, K11O, K27O, K29O, K33O, K48O, or K63O) and the series of DUSP10 mutants retaining only one lysine residue within the binding interface (K185O, K199O, K209O, K222O, K226O, K231O, K260O, K264O, K270O, and K277O) were chemically synthesized and constructed by GenePharma. The specific point mutants, including DUSP10-K231R and Ubiquitin-K48R, were generated using the QuikChange Site-Directed Mutagenesis Kit (Agilent Technologies) according to the manufacturer’s protocol. HEK293T cells were transfected with these plasmids or the empty vector control using Lipofectamine 2000 (Invitrogen) and harvested 48 hours post-transfection for biochemical analyses.

**siRNA Transfection**

For transient knockdown studies, HK-2 cells were transfected with DUSP10-specific small interfering RNAs (siRNA) or a non-targeting negative control (NC) using Lipofectamine 2000 (Invitrogen, CA, USA). The primer and oligonucleotide sequences for gene silencing are listed in Supplementary Table S2. Cells were subjected to hypoxia/reoxygenation (H/R) treatment 48 hours after transfection.

**Lentiviral Transduction**

Stable TRIM29 or DUSP10 knockdown and control HK-2 cell lines were established by lentivirus transduction with TRIM29-specific shRNA (shTRIM29), DUSP10-specific shRNA (shDUSP10), or non‑targeting shRNA (shNC; Sigma‑Aldrich). The specific target sequences are provided in Supplementary Table S2. Knockdown efficiency was confirmed by Western blot. Subsequently, the established cell lines were subjected to hypoxia/reoxygenation (H/R) treatment.

**Adenoviral Transduction**

For overexpression studies, HK-2 cells were transduced with adenoviruses expressing human DUSP10 (Ad-DUSP10) or an empty vector (Ad-EV) (GenePharma, Shanghai, China) at a multiplicity of infection (MOI) of 50. Following a 10-hour incubation, the medium was replaced, and cells were cultured for an additional 48–72 hours before functional assays.

**Histological Staining**

Kidney tissue sections were fixed in 10% formalin, embedded in paraffin, and sectioned. After deparaffinization, sections were sequentially stained with hematoxylin and eosin, dehydrated, and examined under a microscope. The extent of I/R-induced injury was evaluated in randomly selected microscopic fields (Olympus, Tokyo, Japan) from each mouse (n=5 per group) by two experienced, blinded renal pathologists using the established Jablonski grading scale (0-4).

**Immunofluorescence (IF) and Immunohistochemistry (IHC)**

For IHC staining, kidney tissues were fixed, dehydrated, embedded in paraffin, and sectioned at a thickness of 4-5 µm. Sections were incubated with an appropriate primary antibody (details provided in Table S3), followed by a corresponding secondary antibody.

For IF staining of kidney sections, after antigen retrieval and blocking, the sections were incubated overnight at 4 °C with a specific primary antibody (see Table S3). After washing, sections were incubated with a fluorophore-conjugated secondary antibody (Boster Biological Technology, SA1074) for 1 hour at room temperature in the dark.

For IF staining of HK-2 cells, cells were fixed with 4% paraformaldehyde for 15 minutes, permeabilized with 0.1% Triton X-100, and blocked with 5% BSA for 30 minutes. Then, the cells were incubated with a specific primary antibody (see Table S3) overnight at 4°C. Next, they were incubated with a fluorophore-conjugated secondary antibody (Boster Biological Technology, SA1074) for 1 hour at room temperature in the dark. For the co-localization analysis, 293T cells transfected with Flag-TRIM29 and HA-DUSP10 were co-incubated with primary antibodies against Flag and HA tags overnight at 4°C, followed by incubation with a mixture of distinct fluorophore-conjugated secondary antibodies.

Following incubation with the secondary antibodies, all sections and cells were washed, and nuclei were counterstained with DAPI. Routine fluorescence images were captured using a fluorescence confocal microscope (Nikon, Tokyo, Japan). Ultra-high-resolution images for TRIM29 and DUSP10 co-localization were acquired using a STELLARIS 5 super-resolution white light confocal microscope (Leica, Germany).

**Quantitative Real-Time PCR (qRT-PCR)**

Total RNA was isolated from kidney tissues and HK-2 cells using Trizol reagent (ThermoFisher Scientific) according to the manufacturer's instructions. The isolated RNA was then reverse-transcribed into cDNA using the SuperScript First-Strand Synthesis System (Invitrogen). Quantitative real-time PCR was performed with GAPDH as an internal control. The relative mRNA expression levels, calculated using the 2^-ΔΔCt^ method, are expressed as fold change relative to the control group. The primer sequences used are listed in Supplementary Table S2.

**Immunoblotting**

Total protein was extracted from tissues or cells using RIPA lysis buffer containing protease and phosphatase inhibitors. The supernatant was collected by centrifugation for measurement of protein concentration. Equal amounts of protein were separated by SDS-PAGE and transferred onto PVDF membranes. The membranes were incubated overnight at 4°C with the corresponding primary antibodies (listed in Table S3), followed by incubation with HRP-conjugated secondary antibodies. Protein signals were detected using an ECL detection system (Servicebio, Wuhan, China), and band intensities were quantified using ImageJ software.

**Immunoprecipitation and Mass Spectrometry (IP-MS)**

HK-2 cells transfected with Flag-TRIM29 were lysed and immunoprecipitated with an anti-Flag antibody or IgG. Precipitated proteins were separated by SDS-PAGE, stained with Coomassie Brilliant Blue, and excised. Following standard in-gel tryptic digestion, peptides were analyzed using an EASY-nLC 1200 system coupled to a Thermo Scientific Orbitrap Exploris 480 mass spectrometer. MS data were acquired in data-dependent mode with a full scan resolution of 70,000 and an MS/MS resolution of 17,500. Raw data were processed using Proteome Discoverer 2.4 and searched against the Swiss-Prot human database with a precursor mass tolerance of 10 ppm and fragment mass tolerance of 0.02 Da.

**GST pull down**

To validate the direct interaction between TRIM29 and DUSP10, a GST pull-down assay was performed. Recombinant GST-TRIM29 and GST-DUSP10 proteins were constructed and purified by Yeasen (Shanghai, China). Briefly, the purified GST-fusion proteins or GST controls were immobilized on anti-GST magnetic beads and incubated with purified HA-DUSP10 or Flag-TRIM29 prey proteins, respectively. After extensive washing, the bound proteins were eluted, resolved by SDS-PAGE, and analyzed via immunoblotting.

**Proximity Ligation Assay (PLA)**

To visualize the endogenous physical interaction between TRIM29 and DUSP10 in situ, a Proximity Ligation Assay (PLA) was performed using the Duolink In Situ Red Starter Kit (Millipore-Sigma, MA, USA), following the manufacturer's instructions. Briefly, HK-2 cells seeded on coverslips were fixed with 4% paraformaldehyde for 15 minutes, permeabilized with 0.1% Triton X-100, and blocked with Duolink Blocking Solution for 1 hour at 37°C. The cells were then incubated overnight at 4°C with primary antibodies derived from different species: mouse anti-TRIM29 (Table S3) and rabbit anti-DUSP10 (Table S3). After washing, the cells were incubated with species-specific PLA probes (PLUS and MINUS) for 1 hour at 37°C. Ligation of the connector oligonucleotides was performed for 30 minutes at 37°C, followed by amplification with polymerase for 100 minutes at 37°C. The resulting PLA signals (red fluorescence) were visualized using a fluorescence confocal microscope (Nikon, Tokyo, Japan), and nuclei were counterstained with DAPI.

**Ubiquitination Assay**

To assess whether TRIM29 modulates DUSP10 ubiquitination, cultured cells were treated with the proteasome inhibitor MG132 (10 μM) for 6 h prior to harvesting. Total protein was extracted from kidney tissues or cells using RIPA lysis buffer supplemented with a protease inhibitor cocktail. Following centrifugation, equal amounts of supernatant protein were subjected to immunoprecipitation. For tissue samples (endogenous), an anti-DUSP10 antibody was used. For transfected cells, immunoprecipitation was performed using an anti-HA antibody to isolate the DUSP10 complex. The resulting immunocomplexes were washed extensively, eluted by boiling in loading buffer, resolved by SDS–PAGE, and visualized via immunoblotting using an anti-Ubiquitin antibody.

To determine the specific ubiquitin linkage types, HEK293T cells were co-transfected with HA-DUSP10, Flag-TRIM29, and the indicated linkage-specific Myc-ubiquitin plasmids (as described in Section Plasmid Transfection). Six hours before lysis, cells were treated with MG132 (10 μM). Cell lysates were harvested and subjected to immunoprecipitation using an anti-HA antibody to isolate DUSP10, followed by immunoblotting to analyze ubiquitin chain topology.

To elucidate the interaction domains and identify the specific ubiquitination acceptor sites, Flag-TRIM29 was co-transfected with the corresponding DUSP10 truncation or point mutant plasmids (detailed in Section Plasmid Transfection) in the presence of MG132. Co-immunoprecipitation was performed using anti-HA antibodies, followed by immunoblotting to evaluate the binding interface and the ubiquitination status of the mutants.

**Ubiquitinated proteomics analysis**

HK-2 cells stably expressing either non-targeting control shRNA (shNC) or TRIM29-specific shRNA (shTRIM29) and subjected to hypoxia/reoxygenation (H/R) were lysed and digested. Ubiquitinated peptides were enriched using anti-diGlycine (K-ε-GG) antibody-conjugated beads. The enriched peptides were separated by nano-liquid chromatography (Easy-nLC 1000) and analyzed by LC-MS/MS on a timsTOF HT mass spectrometer operated in Parallel Accumulation-Serial Fragmentation (PASEF) mode. The acquired MS/MS data were processed and quantified using the MaxQuant software (v.1.6.15.0) with a false discovery rate (FDR) set to <1%. Differential ubiquitination analysis was performed based on site intensity.

**Dual-Luciferase Reporter Assay**

The human TRIM29 promoter sequence was amplified and cloned into the pGL3.0 luciferase reporter vector (Promega, WI, USA) to generate the TRIM29-luc construct. HK-2 cells were seeded in multi-well plates and co-transfected with the indicated reporter plasmids (pGL3.0 empty vector or TRIM29-luc), the KLF10 overexpression plasmid (or its corresponding empty vector), and the pRL-TK Renilla luciferase plasmid as an internal control. After 48 hours of transfection, cells were harvested, and luciferase activities were measured using the Dual-Luciferase Reporter Assay System (Promega) according to the manufacturer’s instructions. Firefly luciferase activity was normalized to Renilla luciferase activity for each sample.

**Chromatin Immunoprecipitation (ChIP) Assay**

ChIP assays were performed using the SimpleChIP Enzymatic Chromatin IP Kit (Cell Signaling Technology, MA, USA) according to the manufacturer's protocol. Briefly, HK-2 cells cultured under normal or H/R conditions were cross-linked with 1% formaldehyde to preserve DNA-protein interactions and then quenched with glycine. Cell lysates were sonicated to shear chromatin into fragments. The sheared chromatin was then immunoprecipitated overnight at 4°C using a specific anti-KLF10 antibody or a normal IgG control. The immunoprecipitated DNA was subsequently purified and subjected to quantitative real-time PCR (qPCR) using specific primers flanking the predicted KLF10 binding sites within the TRIM29 promoter region.

**Colorimetric Phosphatase Activity Assay**

To measure the intrinsic catalytic activity of DUSP10, the IP-purified HA-DUSP10 complexes on magnetic beads were subjected to enzymatic evaluation using the SensoLyte® pNPP Protein Phosphatase Assay Kit (Colorimetric; AnaSpec, Cat. No. AS-71105). After standard immunoprecipitation, the target-bound beads were washed extensively with washing buffer, followed by a final wash with the assay buffer provided in the kit. The washed beads were uniformly resuspended in the assay buffer and equally divided into two aliquots. One aliquot was boiled in SDS loading buffer and subjected to Western blot analysis to quantify the recovery of the HA-DUSP10 bait and assess its K48-linked ubiquitination status. The remaining aliquot was incubated with the pNPP reaction mixture at 37 °C for 45 minutes in the dark. Following incubation, the reaction was terminated using the provided stop solution. The sample tubes were placed on a magnetic rack to pellet the beads, and the yellow supernatant was carefully transferred to a 96-well microplate. The optical density (OD) was measured at 405 nm using a microplate reader. Finally, the relative phosphatase activity was calculated from the raw OD405 values and normalized against the corresponding HA-DUSP10 protein levels determined from the parallel Western blot.

**Renal Function Assessment**

Mouse renal function was assessed by measuring blood urea nitrogen (BUN) and serum creatinine (SCr) levels using commercial assay kits (Nanjing Jiancheng Co., Nanjing, China), strictly following the manufacturers' protocols.

**Supplementary Figures**

**Figure S1. KLF10 acts as the upstream transcriptional driver of TRIM29 during ischemic stress.** **(A)** qRT-PCR analysis of TRIM29 mRNA levels in HK-2 cells subjected to H/R stimulation, with or without pre-treatment using the HIF-1α inhibitor (PX-478), NF-κB inhibitor (BAY 11-7082), or ER stress inhibitor (4-PBA). **(B, C)** Western blot and corresponding quantitative analysis of TRIM29 protein expression in HK-2 cells under the same treatment conditions as in (A). **(D)** Bioinformatic prediction of potential transcription factors binding to the *TRIM29* promoter region (-2000 bp to 100 bp), including predicted binding sequences and scores. **(E)** Heatmap derived from RNA-sequencing data showing the differential expression of the predicted candidate transcription factors (including KLF10, MAFF, and STAT3) between the Sham and RIRI groups. **(F)** Western blot analysis confirming the successful overexpression of these selected candidate transcription factors in HK-2 cells. **(G)** qRT-PCR analysis of TRIM29 mRNA levels in HK-2 cells following the validated overexpression of the indicated transcription factors. **(H)** Western blot and quantitative analysis of TRIM29 and KLF10 protein expression in HK-2 cells, demonstrating that KLF10 knockdown (Lv-shKLF10) abolishes H/R-induced TRIM29 upregulation. **(I)** Dual-luciferase reporter assay evaluating the direct transactivation of the *TRIM29* promoter by KLF10. Relative luciferase activity is expressed as fold change, with the *TRIM29*-luc + Vector control group arbitrarily set to 1 after normalization to Renilla luciferase activity. **(J)** Schematic representation of the primer sequences designed for the ChIP assay. **(K)** ChIP-qPCR analysis verifying the direct binding of KLF10 to the *TRIM29* promoter, which is significantly enhanced under H/R conditions. Data are presented as mean ± SEM. ****P < 0.0001, ns indicates not significant.

**Figure S2. Validation of knockout mice generation and analysis of downstream signaling pathways. (A)** Schematic diagram illustrating the strategy for generating renal tubule-specific TRIM29 knockout (CKO) mice by crossing TRIM29 Flox/Flox mice with Cdh16-Cre transgenic mice. **(B)** Genotyping of TRIM29 *^Flox/Flox^* and TRIM29 CKO mice using PCR analysis and agarose gel electrophoresis. PCR products showing the specific bands for Cre and the floxed TRIM29 allele confirmed the successful generation of the knockout mice. **(C, D)** Representative western blot analysis (C) and corresponding quantification (D) confirming the reduced expression of TRIM29 protein in the kidney tissues of CKO mice compared with TRIM29 *^Flox/Flox^* littermates. **(E)** Quantitative analysis of the tubular injury score evaluating histological damage (including tubular dilation, brush border loss, and cast formation) in kidney sections from TRIM29 *^Flox/Flox^* and TRIM29 CKO mice following Sham or RIRI operations. **(F, G)** Quantitative analysis of the relative phosphorylation levels of NF-κB pathway markers (p-IKKβ, p65, and p-IκBα) in kidney tissues (F) and HK-2 cells (G). **(H, I)** Quantitative analysis of relative apoptosis-related proteins (BAX and BCL-2) in kidney tissues (H) and HK-2 cells (I). **(J)** Quantitative analysis of the relative phosphorylation levels of JNK, p38, and ERK in kidney tissues from TRIM29 *^Flox/Flox^* and TRIM29 CKO mice following Sham or RIRI operations. **(K)** Quantitative analysis of the relative phosphorylation levels of JNK, p38, and ERK in HK-2 cells transfected with shNC or shTRIM29 under control or H/R conditions. Data are presented as mean ± SEM. Statistical significance was determined by Student's t-test for (D), and two-way ANOVA for (E–K). **P < 0.01, ***P < 0.001, ****P < 0.0001, ns: not significant.

**Figure S3. Identification of TRIM29-interacting proteins, quantification of endogenous DUSP10 expression, and validation of downstream targets and signaling.** **(A)** Mass spectrometry (MS) analysis of proteins immunoprecipitated with Flag-TRIM29 in HK-2 cells, listing potential binding partners including DUSP10. **(B)** Representative images and quantitative analysis of clinical immunohistochemistry (IHC) staining confirming significant DUSP10 downregulation in human kidney biopsies from AKI patients compared to normal controls. **(C, D)** Quantitative analysis of relative DUSP10 protein levels in kidney tissues from Sham and RIRI mice (C) and in HK-2 cells under Control or H/R conditions (D). **(E)** Quantitative analysis of relative DUSP10 protein expression in kidney tissues from *TRIM29* Flox/Flox and *TRIM29* CKO mice subjected to Sham or RIRI operations. **(F)** Quantitative analysis of relative DUSP10 levels in HK-2 cells transfected with shNC or shTRIM29 followed by H/R treatment. **(G)** Co-immunoprecipitation (Co-IP) assay confirming the physical interaction between TRIM29 and PARP1. **(H)** Quantitative analysis of PARP1 protein levels following TRIM29 manipulation, demonstrating that PARP1 stability is unaffected by TRIM29. **(I, J)** Quantitative analysis of relative *Dusp10* mRNA levels in kidney tissues from Sham and RIRI mice (I) and in HK-2 cells under Control or H/R conditions (J). **(K)** Quantitative analysis of relative DUSP10 protein overexpression efficiency in kidney tissues from mice administered AAV9-EV or AAV9-DUSP10. **(L–N)** Quantitative analysis of relative protein levels in kidney tissues from mice pre-treated with AAV9-EV or AAV9-DUSP10 followed by Sham or RIRI operations. (L) Quantitative analysis of the relative phosphorylation levels of MAPK pathway components (p-JNK, p-p38, and p-ERK). (M) Quantitative analysis of the relative phosphorylation levels of NF-κB pathway components (p-IKKβ, p-p65, and p-IκBα). (N) Quantitative analysis of relative apoptosis-related proteins (BAX and BCL-2). **(O)** Quantitative analysis of the tubular injury score evaluating histological damage in kidney sections from AAV9-EV and AAV9-DUSP10 mice following Sham or RIRI operations. **(P)** Quantitative analysis of relative DUSP10 protein overexpression efficiency in HK-2 cells transduced with Ad-EV or Ad-DUSP10. **(Q–S)** Quantitative analysis of signaling pathways and apoptosis markers in HK-2 cells under Control or H/R conditions. (Q) Quantitative analysis of the relative phosphorylation levels of JNK, p38, and ERK. (R) Quantitative analysis of the relative phosphorylation levels of p-IKKβ, p-p65, and p-IκBα. (S) Quantitative analysis of relative BAX and BCL-2 protein levels. Data are presented as mean ± SEM. Statistical significance was determined by Student's t-test for comparisons between two groups (B, K, P), and two-way ANOVA for multiple groups (C–F, I–J, L–O, Q–S). *P < 0.05, **P < 0.01, ***P < 0.001, ****P < 0.0001.

**Figure S4. Quantitative analysis of downstream signaling in rescue experiments and validation of the causal MAPK-to-NF-κB/Apoptosis axis.** **(A–D)** Quantitative analysis of relative protein levels in kidney tissues from *TRIM29* CKO mice pre-treated with AAV9-shNC or AAV9-shDUSP10 followed by RIRI operations. (A) Quantification of relative DUSP10 knockdown efficiency. (B) Quantitative analysis of the relative phosphorylation levels of MAPK pathway components (p-JNK and p-p38). (C) Quantitative analysis of the relative phosphorylation levels of NF-κB pathway components (p-IKKβ, p-p65, and p-IκBα). (D) Quantitative analysis of relative apoptosis-related proteins (BAX and BCL-2). **(E)** Quantitative analysis of the tubular injury score evaluating histological damage (H&E staining) in kidney sections from *TRIM29* CKO mice administered AAV9-shNC or AAV9-shDUSP10 following RIRI, demonstrating that DUSP10 depletion reverses the structural protection conferred by TRIM29 deficiency. **(F–I)** Quantitative analysis of relative protein expression in HK-2 cells with stable *TRIM29* knockdown (shTRIM29) transfected with si-NC or si-DUSP10 prior to H/R treatment. (F) Validation of relative DUSP10 knockdown efficiency. (G) Quantitative analysis of MAPK pathway relative phosphorylation levels. (H) Quantitative analysis of NF-κB pathway relative phosphorylation levels. (I) Quantitative analysis of relative BAX and BCL-2 protein levels. **(J, K)** Representative western blot images (J) and corresponding quantitative analysis (K) validating relative DUSP10 knockdown efficiency in HK-2 cells (shNC vs. shDUSP10). **(L, M)** Representative western blot images (L) and quantitative analysis (M) of NF-κB pathway relative phosphorylation levels (p-IKKβ, p-p65, and p-IκBα) in DUSP10-depleted HK-2 cells (shDUSP10 + H/R) treated with DMSO (vehicle), the JNK specific inhibitor SP600125, or the p38 specific inhibitor SB203580. **(N, O)** Representative western blot images (N) and quantitative analysis (O) of apoptosis-related proteins (BAX and BCL-2) under the same treatment conditions as in (L). Data are presented as mean ± SEM. Statistical significance was determined by Student's t-test for comparisons between two groups (A, F, K) and ANOVA for multiple comparisons (B–E, G–I, M, O). * P < 0.05, **P < 0.01, ***P < 0.001****P < 0.0001.

**Figure S5. Identification of the specific ubiquitin acceptor site and evaluation of DUSP10 intrinsic catalytic activity.** **(A)** A schematic diagram illustrating the construction strategy for DUSP10 single-lysine mutants within the TRIM29-binding domain (aa 168–321). In each mutant construct (listed on the left, e.g., K185, K199), only one specific lysine residue (marked in red) was retained, while all other lysine residues in this region were substituted with arginine (R). These constructs were generated to map the specific ubiquitin acceptor site targeted by TRIM29. **(B)** Representative Co-IP and western blot analysis assessing endogenous K48-linked polyubiquitination of DUSP10. HK-2 cells were transfected with HA-DUSP10 WT or HA-DUSP10 K231R, alongside Flag-TRIM29 overexpression (Flag-T29 OE) or knockdown (si-TRIM29) constructs, and subjected to H/R treatment in the presence of MG132 (10 μM). **(C)** Quantitative fluorometric phosphatase activity assay evaluating the intrinsic catalytic function of the IP-isolated DUSP10 corresponding to the groups in (B). Relative enzymatic activity was normalized to the total amount of HA-DUSP10 bait protein, demonstrating that K231 ubiquitination strictly serves as a degradation signal and does not allosterically inhibit the intrinsic catalytic activity of DUSP10. Data are presented as mean ± SEM. ns indicates not significant (P > 0.05).

**Supplementary Tables**

**Supplementary Table S1: Primer sequences for plasmid construction**

| **TRIM29** | |
| --- | --- |
| TRIM29 Full length | (Forward) gatgacgatgacaaggaattcATGGAAGCTGCAGATGCCTC |
|  | (Reverse) tgatcagcgagctcagaattcTCATGGGGCTTCGTTGGAC |
| TRIM29 1-258aa | (Forward) gatgacgatgacaaggaattcGAAGCTGCAGATGCCTCCAG |
|  | (Reverse) tgatcagcgagctcagaattcCACGGTGCTATGATTCTTGTGC |
| TRIM29 △BB | (Forward 1) gatgacgatgacaaggaattcACTCCCTGCCGAGACGCC |
|  | (Reverse 1) ggacaccgtgggctttgcctccccgccgtg |
|  | (Forward 2) aagcccacggtgtccatcatggagcccgggga |
|  | (Reverse 2) tgatcagcgagctcagaattcTGACAGACCAATGGTTTATTCCAA |
| TRIM29 △CC | (Forward 1) gatgacgatgacaaggaattcACTCCCTGCCGAGACGCC |
|  | (Reverse 1) ctatgattcttgtgctcccgggtctccccgggc |
|  | (Forward 2) gagcacaagaatcatagcaccgtgacagtggag |
|  | (Reverse 2) tgatcagcgagctcagaattcTGACAGACCAATGGTTTATTCCAA |
| TRIM29 259-588aa | (Forward) gatgacgatgacaaggaattcACAGTGGAGGAGGCCAAGGC |
|  | (Reverse) tgatcagcgagctcagaattcTGGGGCTTCGTTGGACCC |
| TRIM29 357-588aa | (Forward) gatgacgatgacaaggaattcCTGCATGAGGACAAGCAGACC |
|  | (Reverse) tgatcagcgagctcagaattcTGGGGCTTCGTTGGACCC |
| **DUSP10** | |
| DUSP10 Full length | (Forward) gatgacgatgacaaggaattcATGCCTCCGTCTCCTTTAGACG |
|  | (Reverse) tgatcagcgagctcagaattcTCACACAACCGTCTCCACGC |
| DUSP10 1-167aa | (Forward) gatgacgatgacaaggaattcATGCCTCCGTCTCCTTTAGACG |
|  | (Reverse) tgatcagcgagctcagaattcCAGGTGACTCTTGCTGCATTTG |
| DUSP10 168-321aa | (Forward) gatgacgatgacaaggaattcCCGAGTCAGGGCCCTGTC |
|  | (Reverse) tgatcagcgagctcagaattcCTCAGCGTTCTCGATGTCAGG |
| DUSP10 322-482aa | (Forward) gatgacgatgacaaggaattcCTCACCCCCATCTTGCCC |
|  | (Reverse) tgatcagcgagctcagaattcCACAACCGTCTCCACGCCC |

**Supplementary Table S2: Primer and oligonucleotide sequences for gene silencing and RT-qPCR**

| **Name** | **Species** | **Forward Sequence (5'-3')** | **Reverse Sequence (5'-3')** |
| --- | --- | --- | --- |
| si-DUSP10 | Human | GGAUCUCAACCUUUGUUUAGA | UAAACAAAGGUUGAGAUCCUG |
| shTRIM29 | Human | CCGGGGACGACCTGCTCAATGTACTCGAGTACATTGAGCAGGTCGTCCTTTTTG | AATTCAAAAAGGACGACCTGCTCAATGTACTCGAGTACATTGAGCAGGTCGTCC |
| shDUSP10 | Human | CCGGGGATCTCAACCTTTGTTTAGACTCGAGTCTAAACAAAGGTTGAGATCCTTTTTG | AATTCAAAAAGGATCTCAACCTTTGTTTAGACTCGAGTCTAAACAAAGGTTGAGATCC |
| **Name** | **Species** | **Forward Sequence (5'-3')** | **Reverse Sequence (5'-3')** |
| TRIM29 | Human | GCCATCCTGGAGCAGAACTT | GAGGGCTGGTATGATGTCCG |
| TRIM29 | Mouse | TCAGTGACTCGGTGCTGTTC | GGAGGGAGGGAAAGCCTTTC |
| DUSP10 | Human | CAGTAACAGCCACCCTCCTG | GACAGTGATCTTGCCCTGCT |
| DUSP10 | Mouse | AGGATCATCAGGCCCAGACT | ATGTGAAGTGGCTGGGATGG |
| GAPDH | Human | GATTTGGTCGTATTGGGCGC | AGTGATGGCATGGACTGTGG |
| Gapdh | Mouse | TTCACCACCATGGAGAAGGC | TGAAGTCGCAGGAGACAACC |
| CCL2 | Human | ATCAATGCCCCAGTCACCTG | TTGGGTTTGCTTGTCCAGGT |
| Ccl2 | Mouse | GTCCCTGTCATGCTTCTGGG | TGCTTGAGGTGGTTGTGGAA |
| CXCL2 | Human | CTGCTGCTCCTGCTCCTG | TGAGACAAGCTTTCTGCCCA |
| Cxcl2 | Mouse | GCTCCTCAGTGCTGCACT | GCAAACTTTTTGACCGCCCT |
| IL1B | Human | CCAGCTACGAATCTCCGACC | GGGAACTGGGCAGACTCAAA |
| Il1b | Mouse | GTGGCAGCTACCTGTGTCTT | CTCTGCTTGTGAGGTGCTGA |
| IL6 | Human | TTCGGTCCAGTTGCCTTCTC | AGCTGCGCAGAATGAGATGA |
| Il6 | Mouse | GCCTTCTTGGGACTGATGCT | GCCACTCCTTCTGTGACTCC |
| IL-8 | Human | TCTGCAGCTCTGTGTGAAGG | TTCCTTGGGGTCCAGACAGA |
| Cxcl15 | Mouse | CTAGGCATCTTCGTCCGTCC | GCTGACTTCACTGGAGTCCC |

**Supplementary Table S3: Antibodies used in this study.**

| **Name** | **Manufacturer** | **Cat number** | **Application** |
| --- | --- | --- | --- |
| TRIM29 | Abcam | #AB244380 | 1:2000 for WB 1:200 for IHC  1:200 for IF |
| TRIM29 | Thermo Fisher | #MA5-38651 | 1:200 for PLA |
| p-IKKβ | CST | #2697 | 1:1000 for WB |
| IKKβ | CST | #8943 | 1:1000 for WB |
| p-p65 | CST | #3031 | 1:1000 for WB |
| p65 | CST | #8242 | 1:1000 for WB |
| p-IκBα | CST | #2859 | 1:1000 for WB |
| p-JNK | CST | #4668 | 1:1000 for WB |
| JNK | CST | #9252 | 1:1000 for WB |
| p-p38 | CST | #4511 | 1:1000 for WB |
| p38 | CST | #9212 | 1:1000 for WB |
| p-ERK | CST | #[5726](https://www.cellsignal.cn/products/primary-antibodies/phospho-p44-42-mapk-erk1-tyr204-erk2-tyr187-d1h6g-mouse-monoclonal-antibody/5726) | 1:1000 for WB |
| ERK | CST | #4695 | 1:1000 for WB |
| DUSP10 | Abcam | #ab228987 | 1:2000 for WB 1:200 for IHC  1:200 for IF and PLA |
| BAX | Proteintech | #50599-2-Ig | 1:20000 for WB |
| BCL2 | Proteintech | #12789-1-AP | 1:5000 for WB |
| Ub | CST | #20326 | 1:1000 for WB |
| K48-Ub | CST | #4289 | 1:1000 for WB |
| PARP1 | CST | #9532 | 1:1000 for WB |
| KLF10 | MCE | HY-P82604 | 1:1000 for WB |
| MAFF | Proteintech | 12771-1-AP | 1:18000 for WB |
| STAT3 | MCE | HY-P80344 | 1:1000 for WB |
| HA-Tag | Proteintech | #66006-2-Ig | 1:20000 for WB; 2 µg for IP |
| Flag-Tag | Proteintech | #20543-1-AP | 1:20000 for WB; 2 µg for IP |
| Myc-Tag | Proteintech | #60003-2-Ig | 1:2000 for WB |
| GAPDH | Proteintech | #10494-1-AP | 1:10000 for WB |
| IgG | Proteintech | #30000-0-AP | 2 µg for IP |

IHC: Immunohistochemistry; WB: Western blotting; IP: Immunoprecipitation; IF: Immunofluorescence; PLA: Proximity Ligation Assay; Abcam (Cambridge, UK); Cell Signaling Technology (Danvers, MA, USA); Thermo Fisher Scientific (Waltham, MA, USA); Proteintech (Hubei, China); ABclonal (Hubei, China)
